# Taxonomic revision and phylogenetic study of the tribe Gobionini in China (Actinopteri, Cypriniformes, Gobionidae), with the discussion on the generic character of the genus *Mesogobio*

**DOI:** 10.3897/zookeys.1293.202134

**Published:** 2026-10-01

**Authors:** Yutian Fang, Zhixian Sun, Yahui Zhao

**Affiliations:** 1 State Key Laboratory of Animal Biodiversity Conservation and Integrated Pest Management, Institute of Zoology, Chinese Academy of Sciences, Beijing, 100101, China State Key Laboratory of Animal Biodiversity Conservation and Integrated Pest Management, Institute of Zoology, Chinese Academy of Sciences Beijing China; 2 Shanghai Universities Key Laboratory of Marine Animal Taxonomy and Evolution, Shanghai Ocean University, Shanghai, 201306, China Shanghai Universities Key Laboratory of Marine Animal Taxonomy and Evolution, Shanghai Ocean University Shanghai China

**Keywords:** Freshwater fish, morphology, phylogeny, taxonomy

## Abstract

The tribe Gobionini, comprising three genera, is widely distributed across Eurasia, with China representing one of the distribution hotspots. Despite numerous species descriptions over the past decades, a comprehensive taxonomic study of this tribe is still lacking. Both morphological comparisons and molecular phylogenetic analysis based on Cytochrome*-b* gene have been included in this study. Our results indicate that the previously reported species diversity within this tribe may have been overestimated, with 12 species from two genera identified in China: *Mesogobio lachneri*, *Gobio cynocephalus*, *G. guentheri*, *G. soldatovi*, *G. coriparoides*, *G. rivuloides*, *G. macrocephalus*, *G. tenuicorpus*, *G. acutipinnatus*, *G. huanghensis*, *G. tumenensis*, and *G. fushunensis*. The diagnostic key for separating *Gobio* and *Mesogobio* is revised based on swim bladder morphology. In the genus *Gobio*, the swim bladder is well developed, with a total length approximately two-thirds that of the coelom. In contrast, the swim bladder of the genus *Mesogobio* is reduced, with a total length of approximately half that of the coelom. A diagnostic key to the species distributed in China is also provided.

## Introduction

The tribe Gobionini is a group of freshwater fish belonging to the family Gobionidae (Yang et al. 2006; Tan and Armbruster 2018). Currently, three genera are assigned to this tribe: *Gobio* Cuvier, 1816, *Romanogobio* Bănărescu, 1961 and *Mesogobio* Bănărescu & Nalbant, 1973 (Yang et al. 2006; Fang et al. 2025). The genus *Gobio* was established by Cuvier (1816), with *G. gobio* (Linnaeus, 1758) as its type species. As the most widespread genus in the family Gobionidae, it is distributed across Eurasia and also contains the highest number of species among the Gobionidae (Fricke et al. 2026). The genus *Acanthogobio* was established by Herzenstein (1892), but recent phylogenies confirmed it as a junior synonym of *Gobio* (Tang et al. 2011; Fang et al. 2025).

Originally described as a subgenus of *Gobio* by Bănărescu (1961), *Romanogobio* was defined with *R. kesslerii* (Dybowski, 1862) as its type species. This subgenus was later elevated to genus level in subsequent studies (Naseka 1996; Bogutskaya and Naseka 1997). Most *Romanogobio* species are distributed in Europe, while three have been recorded from China, including *R. amplexilabris* Bănărescu & Nalbant, 1973, *R. johntreadwelli* Bănărescu & Nalbant, 1973 and *R. tenuicorpus* (Mori, 1934) (Bănărescu and Nalbant 1973; Bogutskaya and Naseka 1997). Among them, *R. amplexilabris* and *R. johntreadwelli* have been confirmed as junior synonyms of *G. rivuloides* (Fang et al. 2025), whereas the reassignment of *R. tenuicorpus* from the genus *Gobio* to *Romanogobio* has also been questioned (Yang et al. 2006; Tang et al. 2011; Zhang et al. 2016).

Bănărescu and Nalbant (1973) established the genus *Mesogobio* and designated *M. lachneri* Bănărescu & Nalbant, 1973 as the type species. It is distinguished from *Gobio* by two key characters: lips with tiny papillae, and the anterior chamber of the swim bladder enclosed in fibrous capsule (Bănărescu and Nalbant 1973). Chang (in Zheng et al. 1980) described *M. tumenensis* from the Tumen River and added the presence of a horny margin on jaws as a diagnostic key to distinguish *Mesogobio* from *Gobio*. Subsequent phylogenetic analyses of Gobionidae revealed that *M. tumenensis* is nested within the genus *Gobio*, and suggested its taxonomic status as *G. tumenensis* (Yang et al. 2006; Tang et al. 2011). However, these studies did not include sequence data for the type species, *M. lachneri*, and the taxonomic status of *Mesogobio* therefore remains unresolved.

Despite some current taxonomic issues, China is a species diversity hotspot for the tribe Gobionini, including three genera and 14 species (Luo et al. 1977; Bogutskaya and Naseka 1997; Yue 1998; Xie et al. 2007; Zhang et al. 2016; Fang et al. 2025):

*Mesogobio lachneri* Bănărescu & Nalbant, 1973,

*M. tumenensis* Chang, 1980,

*Gobio cynocephalus* Dybowski, 1869,

*G. guentheri* (Herzenstein, 1892),

*G. soldatovi* Berg, 1914,

*G. coriparoides* Nichols, 1925,

*G. rivuloides* Nichols, 1925,

*G. macrocephalus* Mori, 1930,

*G. lingyuanensis* Mori, 1934,

*G. acutipinnatus* Men’shikov, 1939,

*G. huanghensis* Lo, Yao & Chen, 1977,

*G. fushunensis* Xie, Li & Xie, 2007,

*G. multipunctatus* Vasil’eva, Mamilov & Sharakhmetov, 2023,

*Romanogobio tenuicorpus* (Mori, 1934).

The latest comprehensive taxonomic revision dates to Yue (1998), nearly three decades ago. Since then, some new species have been described (Xie et al. 2007; Vasil’eva et al. 2023), and several species have been confirmed as synonyms of others (Fang et al. 2025). It is therefore necessary to carry out a detailed taxonomic revision on the tribe Gobionini to resolve the existing taxonomic ambiguities and provide a reliable diagnostic key to these species.

In our study, we examined specimens of the tribe Gobionini, including several type specimens. We further performed a phylogenetic analysis based on the *cytochrome b* (*cytb*) gene. Based on morphological and molecular evidence, we revised the tribe Gobionini distributed in China, and a practical diagnostic key to these species is also provided.

## Materials and methods

### Specimen collection, examination, and preservation

Most of the specimens used in this study were collected between 2022 and 2025 from China, from the Yellow, Haihe, Luanhe, Liaohe, Yalu, Tumen, Suifen, Amur and Irtysh river drainages. Some coastal rivers in the Liaodong Peninsula are also included. Fish were collected using fish traps and hand nets. After collection, the specimens were fixed in 10% formaldehyde and stored in 70% ethanol for morphological study, or directly fixed in 95% ethanol for phylogenetic study. All of our specimens are preserved at the Institute of Zoology, Chinese Academy of Sciences, Beijing, China (**ASZIB**, abbreviation follows Sabaj 2020).

The holotype and paratypes of *Mesogobio tumenensis* is preserved at ASIZB. The syntypes of *G. huanghensis* are stored at the Institute of Hydrobiology, Chinese Academy of Sciences, Hubei, China (**IHB**). The holotypes of *G. soldatovi minulus*, *G. coriparoides*, and *G. shansiensis* are preserved at the American Museum of Natural History, New York, the United States (**AMNH**). The syntypes of *G. acutipinnatus* and the lectotypes of *G. soldatovi* are preserved at the Zoological Institute, Russian Academy of Sciences, St. Petersburg, Russia (**ZIN**). The holotype of *Mesogobio lachneri* is preserved at the National Museum of Natural History, Smithsonian Institution, Washington, the United States (**USNM**).

### Morphological study

Measurements were taken on the left side of the specimens using a digital caliper, rounded to 0.01 mm; all measurements were taken point to point. Counts were also made on the left side of the specimens. If the scales or fin rays were damaged from the left side, counts were made on the right. Methods for counts and measurements followed Zhao and Sun (2021). The lateral line scales include the scales on the caudal-fin base. The last two rays in dorsal and anal-fin based on a single pterygiophore were counted as one ray. Only adult specimens (larger than 60 mm SL) were measured; juveniles and smaller, bent, or damaged specimens were not included in the measurements (Kottelat and Persat 2005). Method for determining the position of anus followed Fang et al. (2025). The Mann–Whitney U tests (MWU tests) followed by Bonferroni correction were performed in R (v. 4.6.1) to assess differences in morphological characters between closely related species. Adjusted *p*-values < 0.05 were considered statistically significant. In order to obtain a general assessment of external morphological differences, 19 measurable traits, log_10_-standardized to eliminate the allometric effects, were subjected to principal component analysis (PCA) using Past v. 4.17 (Hammer et al. 2001).

### Molecular phylogenetic study

Molecular phylogenetic study was based on the mitochondrial Cytochrome-*b* (*cytb*)genes. DNA was extracted from the pelvic fin on the right side of the fish. The *cytb* gene was amplified using primers L14724 (5’-GACTTGAAAAACCACCGTTG-3’) and H15915 (5’-CTCCGATCTCCGGATTACAAGAC-3’) following Xiao et al. (2001). Sequencing reactions were performed according to the operating instructions of BigDye Terminator v. 3.1 (BDT), with 1.0 μL of primer (3.2 pmol/μL), 1.0 μL of template DNA, 0.4 μL of BigDye® Terminator v. 3.1, 1.8 μL of double distilled water (dd H2O), and 0.8 μL sequencing buffer for a total reaction volume of 5.0 mL. The thermo-cycling conditions were based on the protocol provided by Beijing Tianyi Huiyuan Biotechnology Co. Ltd. (China): initial denaturation for 5 min at 94 °C, denaturation for 10 s at 94 °C, annealing for 5 s at 55 °C, and extension for 90 s at 60 °C. After 25 cycles, a final extension was performed at 60 °C for 3 min and the polymerase chain reaction (PCR) products were preserved at 4 °C. Sequencing was performed on an Applied Biosystems 3730xl DNA sequencer by Beijing Tianyi Huiyuan Biotechnology Co. Ltd. (China).

The sequencing results were assembled using SeqMan (Swindell and Plasterer 1997), and other sequences were acquired from the NCBI database. The voucher ID of each individual and GenBank accession number are given in Table 1. Seventy-three *cytb* sequences were included in the molecular phylogenetic analyses. *Hemibarbus labeo* (Pallas, 1776), *Pseudorasbora parva* (Temminck & Schlegel, 1846), *Acheilognathus macropterus* (Bleeker, 1871), *Pseudogobio vaillanti* (Sauvage, 1878), *Gnathopogon polytaenia* (Nichols, 1925), and *Coreoleuciscus aeruginos* Song & Bang, 2015 were used as outgroups. Nucleotide sequence alignment was conducted using MEGA 11 (Kumar et al. 2018) with ClustalW. ModelFinder (Kalyaanamoorthy et al. 2017) was used to select the best-fit model using Bayesian information criterion (BIC). The Bayesian inference (BI) phylogenies were inferred using MrBayes v. 3.2.6 (Ronquist et al. 2012) under the GTR+F+I+G4 model (two parallel runs of 2,000,000 generations each), with the initial 25% of sampled data discarded as burn-in. The final phylogenetic trees were modified on the TVBOT (https://chiplot.online/tvbot.html, Xie et al. 2023). Each sequence was labeled with its own taxonomic nomenclature, and the evolutionary divergence of sequence pairs between and within groups (i.e., species) was estimated using the Kimura 2-parameter model (Kimura 1980).

**Table 1.** Voucher codes, sampling localities, and accession number of tribe Gobionini species and outgroup for molecular phylogenetic analyses.

**Voucher Code**	**Species**	**Locality**	**Drainage**	**Accession no**.	**Source**
FSJF 891	*Romanogobio banaticus*	Rominia	middle Nera R., Danube River drainage	AY952329	Witte (Direct Submission)
FSJF 343	*Romanogobio kesslerii*	Ukraine	middle Dniestr R.	AY952328	Witte (Direct Submission)
FSJF 889	*Romanogobio uranoscopus*	Rominia	middle Nera R., Danube River drainage	AY952331	Witte (Direct Submission)
HDBI 1292-773	*Romanogobio benacensis*	Kamenita Vrata, Croatia	Mirna R.	MG791926	Jelic et al. 2018
HDBI 1323-771	*Romanogobio benacensis*	Kamenita Vrata, Croatia	Mirna R.	MG791924	Jelic et al. 2018
ASIZB 246569	*Mesogobio lachneri*	Linjiang City, Jilin Province, China	Yalu R.	–	This study
ASIZB 246570	*Mesogobio lachneri*	Linjiang City, Jilin Province, China	Yalu R.	–	This study
ASIZB 241488	*Mesogobio lachneri*	Linjiang City, Jilin Province, China	Yalu R.	–	This study
MNCN AT4759	*Gobio gobio*	Slovenia	Sevnica R.	HM560092	Perea et al. 2010
IHB 0210043	*Gobio gobio*	Germany	Rhine R.	AY953007	Yang et al. 2006
ASIZB 241464	*Gobio acutipinnatus*	Fuyun County, Xinjiang Uygur Autonomous Region, China	Irtysh R.	–	This study
ASIZB 241484	*Gobio acutipinnatus*	Burqin County, Xinjiang Uygur Autonomous Region, China	Irtysh R.	–	This study
ASIZB 248277	*Gobio multipunctatus*	Yumin County, Xinjiang Uygur Autonomous Region, China	Emin R.	–	This study
IHB 0210045	*Gobio macrocephalus*	China	Amur R.	AY953006	Yang et al. 2006
ASIZB 248273	*Gobio macrocephalus*	Wangqing County, Jilin Province, China	Tumen R.	–	This study
ASIZB 241451	*Gobio macrocephalus*	Tonghua County, Jilin Province, China	Tumen R.	–	This study
ASIZB 248272	*Gobio lingyuanensis*	Xinglong County, Hebei Province, China	Luanhe R.	–	This study
ASIZB 248274	*Gobio lingyuanensis*	Pingquan County, Hebei Province, China	Luanhe R.	–	This study
ASIZB 248270	*Gobio soldatovi*	Fengcheng City, Liaoning Province, China	Yalu R.	–	This study
ASIZB 248275	*Gobio soldatovi*	Xunke County, Heilongjiang Province, China	Amur R.	–	This study
ASIZB 248276	*Gobio soldatovi*	Harbin City, Heilongjiang Province, China	Amur R.	–	This study
IHB 0210041	*Gobio cynocephalus*	China	Amur R.	AY953005	Yang et al. 2006
UAIC 14387.01	*Gobio cynocephalus*	–	–	JN003328	Tang et al. 2011
ASIZB 242081	*Gobio cynocephalus*	Xunke County, Heilongjiang Province, China	Amur R.	–	This study
IHB 2001901	*Gobio tumenensis*	China	Tumen R.	AY953008	Yang et al. 2006
ASIZB 249382	*Gobio tumenensis*	Wangqing County, Jilin Province, China	Tumen R.	–	This study
ASIZB 249383	*Gobio tumenensis*	Wangqing County, Jilin Province, China	Tumen R.	–	This study
–	*Gobio huanghensis*	Hainan Tibetan Autonomous Prefecture, Qinghai Province, China	Yellow R.	FJ904648	Qi and Yang 2009
ASIZB 242021	*Gobio huanghensis*	Xing County, Shanxi Province, China	Yellow R.	–	This study
ASIZB 242022	*Gobio huanghensis*	Xing County, Shanxi Province, China	Yellow R.	–	This study
ASIZB 242025	*Gobio huanghensis*	Baode County, Shanxi Province, China	Yellow R.	–	This study
ASIZB 242026	*Gobio huanghensis*	Liulin County, Shanxi Province, China	Yellow R.	–	This study
ASIZB 242030	*Gobio huanghensis*	Hangjin Banner, Inner Mongolia Autonomous Region, China	Yellow R.	–	This study
ASIZB 242040	*Gobio huanghensis*	Lin County, Shanxi Province, China	Yellow R.	–	This study
IHB 0411036	*Gobio coriparoides*	–	–	JN003326	Tang et al. 2011
–	*Gobio coriparoides*	Luonan County, Shaanxi Province, China	Luohe R., Yellow River Drainage	EU934492	Liu et al. 2010
ASIZB 241456	*Gobio coriparoides*	Jingle County, Shanxi Province, China	Fenhe R., Yellow River Drainage	–	This study
ASIZB 242015	*Gobio coriparoides*	Jingle County, Shanxi Province, China	Fenhe R., Yellow River Drainage	–	This study
ASIZB 242033	*Gobio coriparoides*	Lan County, Shanxi Province, China	Fenhe R., Yellow River Drainage	–	This study
ASIZB 242064	*Gobio fushunensis*	Fengcheng City, Liaoning Province, China	Dayanghe R.	–	This study
ASIZB 242079	*Gobio fushunensis*	Tonghua County, Jilin Province, China	Yalu R.	–	This study
ASIZB 242080	*Gobio fushunensis*	Tonghua County, Jilin Province, China	Yalu R.	–	This study
ASIZB 242038	*Gobio guentheri*	Jishishan Bonan, Dongxiang and Salar Autonomous County, Gansu Province, China	Yinchuan R., Yellow River Drainage	–	This study
ASIZB 242039	*Gobio guentheri*	Jishishan Bonan, Dongxiang and Salar Autonomous County, Gansu Province, China	Yinchuan R., Yellow River Drainage	–	This study
ASIZB 242061	*Gobio guentheri*	Liulin County, Shanxi Province, China	Yellow R.	–	This study
–	*Gobio tenuicorpus*	Nehe City, Heilongjiang Province, China	Amur R.	OP354026	Ni et al. 2023
–	*Gobio tenuicorpus*	Xunke County, Heilongjiang Province, China	Amur R.	OP354030	Ni et al. 2023
–	*Gobio tenuicorpus*	Heihe City, Heilongjiang Province, China	Amur R.	OP354033	Ni et al. 2023
–	*Gobio tenuicorpus*	Xin Barag Right Banner, Inner Mongolia Automous Region, China	Amur R.	OP354040	Ni et al. 2023
–	*Gobio tenuicorpus*	Chengde County, Hebei Province, China	Luanhe R.	OP354043	Ni et al. 2023
–	*Gobio tenuicorpus*	Chengde County, Hebei Province, China	Luanhe R.	OP354044	Ni et al. 2023
ASIZB 239619	*Gobio tenuicorpus*	Luanping County, Hebei Province, China	Luanhe R.	–	This study
ASIZB 239621	*Gobio tenuicorpus*	Luanping County, Hebei Province, China	Luanhe R.	–	This study
ASIZB 248269	*Gobio tenuicorpus*	Chengde County, Hebei Province, China	Luanhe R.	–	This study
ASIZB 248290	*Gobio tenuicorpus*	Xinmin City, Liaoning Province, China	Liaohe R.	–	This study
ASIZB 248291	*Gobio tenuicorpus*	Xinmin City, Liaoning Province, China	Liaohe R.	–	This study
–	*Gobio rivuloides*	Yushe County, Shanxi Province, China	Zhuozhanghe R., Haihe River drainage	OP354083	Ni et al. 2023
–	*Gobio rivuloides*	Yushe County, Shanxi Province, China	Zhuozhanghe R., Haihe River drainage	OP354084	Ni et al. 2023
ASIZB 219283	*Gobio rivuloides*	Huguan County, Shanxi Province, China	Xihe R., Haihe River Drainage	–	This study
ASIZB 239687	*Gobio rivuloides*	Shangyi County, Hebei Province, China	Yanghe R., Haihe River Drainage	–	This study
ASIZB 241457	*Gobio rivuloides*	Pingshan County, Hebei Province, China	Hutuohe R., Haihe River Drainage	–	This study
ASIZB 242027	*Gobio rivuloides*	Luonan County, Shaanxi Province, China	Luohe R., Yellow River Drainage	–	This study
ASIZB 242028	*Gobio rivuloides*	Luonan County, Shaanxi Province, China	Luohe R., Yellow River Drainage	–	This study
ASIZB 241459	*Gobio meridionalis*	Xing County, Shanxi Province, China	Yellow R.	–	This study
ASIZB 242016	*Gobio meridionalis*	Bohaiwan County, Inner Mongolia Autonomous Region, China	Yellow R.	–	This study
ASIZB 242018	*Gobio meridionalis*	Bohaiwan County, Inner Mongolia Autonomous Region, China	Yellow R.	–	This study
ASIZB 242062	*Gobio meridionalis*	Daning County, Shanxi Province, China	Yellow R.	–	This study
**Outgroup**					
–	*Acheilognathus macropterus*	–	–	EF483935	Hwang et al. 2014
SXAF20200512	*Hemibarbus labeo*	Shaoxing City, Zhejiang Province, China	Qiantang R.	MT478137	Sun et al. 2020
SUC-1290	*Coreoleuciscus aeruginos*	Jeollanam-do, South Korea	Deokcheon R., Nakdonggang River drainage	MW192440	Kim et al. 2021
–	*Gnathopogon polytaenia*	–	–	KM086722	Tao and Zhao, 2016
–	*Pseudorasbora parva*	–	–	JF802126	Chen and You (Direct Submission)
FDZM-PVKD20100701	*Pseudogobio vaillanti*	Kuandian Manchu Autonomous County, Liaoning Province, China	Yalu R.	KU314695	Li et al. 2018

## Results

### Molecular results

**Figure 1.**
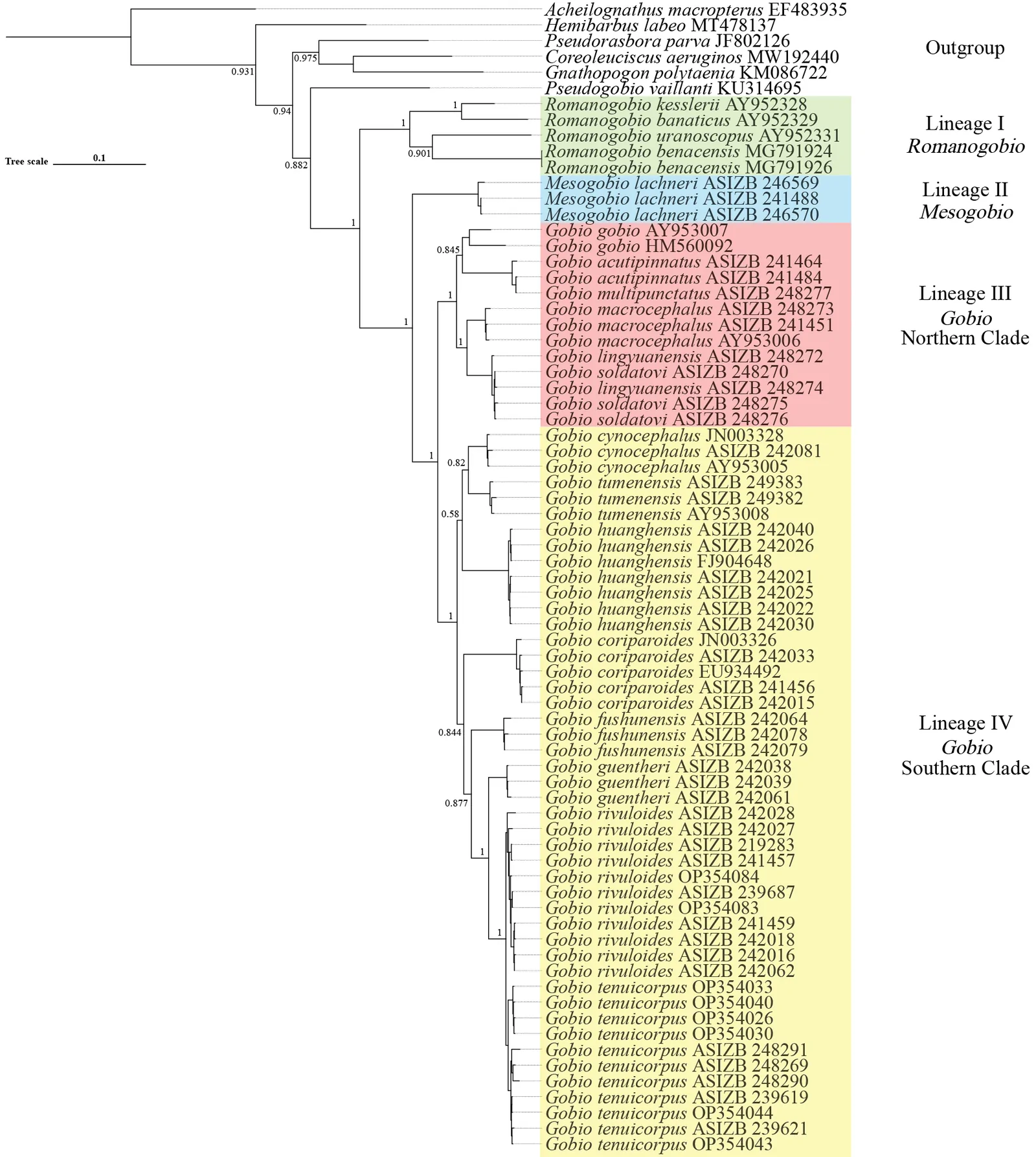
Molecular phylogenetic tree of the tribe Gobionini reconstructed by the Bayesian inference method based on the *cytb* sequence. Bayesian posterior probabilities (BPP) are given at the nodes.

The Bayesian inference tree based on 1140 bp of the *cytb* gene, comprising 67 individuals from 19 species of the tribe Gobionini, is shown in Fig. 1. Four distinct clades were recovered, representing to *Romanogobio*, *Mesogobio* and two clades within *Gobio*. *Mesogobio* and *Gobio* formed a sister-group relationship (BPP = 1), with *Romanogobio* as the sister to the clade comprising *Mesogobio* and *Gobio* (BPP = 1).

The genus *Mesogobio* is a monophyletic group containing only *M. lachneri*. Although *Mesogobio tumenensis* had been previously assigned to this genus, phylogenetic tree revealed that it is nested within the clade of the genus *Gobio* and forms a sister group with *G. cynocephalus* This result is consistent with previous studies (Yang et al. 2006; Tang et al. 2011).

Within the genus *Gobio*, two distinct clades are indicated. The northern clade includes *G. acutipinnatus*, *G. multipunctatus*, *G. macrocephalus*, *G. lingyuanensis*, *G. soldatovi*, and *G. gobio*, representing the species inhabiting northern drainages, such as the Amur, Tumen, and Irtysh river drainages, as well as some European rivers. The southern clade, including *G. cynocephalus*, *G. tumenensis*, *G. huanghensis*, *G. coriparoides*, *G. fushunensis*, *G. guentheri*, *G. rivuloides*, and *G. tenuicorpus*, represents the species mainly distributed in some southern rivers, like the Yellow, Haihe, and Luanhe river drainages.

The genetic distances of the *cytb* gene in tribe Gobionini are shown in Table 2. The maximum interspecific genetic distance (0.1162) within the genus *Gobio* was recorded between *G. acutipinnatus* and *G. coriparoides*, whereas the minimum interspecific genetic distance (0.0115) occurred between *G. rivuloides* and *G. tenuicorpus*. Certain pairwise genetic distances are notably low, for instance the 0.0049 divergence between *G. acutipinnatus* and *G. multipunctatus*, and the 0.0038 divergence between *G. soldatovi* and *G. lingyuanensis*, which are comparable to the intraspecific genetic distances within each species.

**Table 2.** Mean K2P genetic distances within and among species of the tribe Gobionini based on the *cytb* gene. *Acheilognathus macropterus* was used as the outgroup.

	**Intraspecific**	**1**	**2**	**3**	**4**	**5**	**6**	**7**	**8**	**9**	**10**	**11**	**12**	**13**	**14**	**15**	**16**	**17**	**18**	**19**
1 *Acheilognathus macropterus*	n/c																			
2 *Romanogobio kesslerii*	n/c	0.2250																		
3 *Romanogobio banaticus*	n/c	0.2250	0.0933																	
4 *Romanogobio uranoscopus*	n/c	0.2347	0.1446	0.1711																
5 *Romanogobio benacensis*	n/c	0.2592	0.1635	0.1687	0.1604															
6 *Mesogobio lachneri*	0.0065	0.2140	0.1677	0.1791	0.1925	0.1731														
7 *Gobio gobio*	0.0570	0.2320	0.1710	0.1829	0.1903	0.1856	0.1185													
8 *Gobio acutipinnatus*	0.0097	0.2422	0.1695	0.1976	0.1910	0.1841	0.1485	0.0777												
9 *Gobio multipunctatus*	n/c	0.2422	0.1695	0.1969	0.1910	0.1792	0.1448	0.0762	0.0049											
10 *Gobio macrocephalus*	0.0059	0.2145	0.1654	0.1734	0.1878	0.1731	0.1133	0.0621	0.0777	0.0761										
11 *Gobio lingyuanensis*	0.0053	0.2148	0.1616	0.1770	0.1839	0.1804	0.1186	0.0676	0.0833	0.0833	0.0492									
12 *Gobio soldatovi*	0.0023	0.2132	0.1596	0.1754	0.1852	0.1780	0.1177	0.0668	0.0818	0.0818	0.0498	0.0038								
13 *Gobio cynocephalus*	0.0047	0.2142	0.1577	0.1865	0.1939	0.1968	0.1073	0.0865	0.0978	0.0974	0.0827	0.0782	0.0781							
14 *Gobio tumenensis*	0.0065	0.2155	0.1590	0.1850	0.1937	0.1924	0.1090	0.0835	0.1082	0.1082	0.0821	0.0839	0.0834	0.0446						
15 *Gobio huanghensis*	0.0020	0.2163	0.1731	0.2006	0.1885	0.1923	0.1222	0.0981	0.1132	0.1115	0.0960	0.0976	0.0974	0.0662	0.0703					
16 *Gobio coriparoides*	0.0055	0.2278	0.1796	0.2069	0.2138	0.1931	0.1439	0.1090	0.1162	0.1151	0.0999	0.0931	0.0945	0.0779	0.0837	0.0999				
17 *Gobio fushunensis*	0.0071	0.2294	0.1623	0.1916	0.1902	0.1857	0.1208	0.0980	0.1029	0.1029	0.0897	0.0977	0.0972	0.0719	0.0710	0.0825	0.0897			
18 *Gobio guentheri*	0.0029	0.2159	0.1689	0.1836	0.2031	0.1901	0.1247	0.0967	0.1143	0.1137	0.0866	0.0992	0.0994	0.0730	0.0679	0.0949	0.0833	0.0662		
19 *Gobio rivuloides*	0.0060	0.2180	0.1689	0.1910	0.1949	0.1911	0.1219	0.0981	0.1143	0.1148	0.0833	0.0927	0.0929	0.0720	0.0765	0.0897	0.0896	0.0691	0.0421	
20 *Gobio tenuicorpus*	0.0079	0.2174	0.1644	0.1865	0.1906	0.1889	0.1232	0.0981	0.1142	0.1145	0.0826	0.0907	0.0898	0.0732	0.0758	0.0889	0.0912	0.0711	0.0429	0.0115

### Taxonomy

Based on morphological comparisons and molecular phylogenetic analysis, we conclude that there are two genera and 12 valid species distributed in China. The genus *Mesogobio* Bănărescu & Nalbant, 1973 is valid and monotypic. The genus *Gobio* Cuvier, 1816 comprises the most species within the tribe Gobionini in China, with a total of 11 species occurring here: *Gobio cynocephalus* Dybowski, 1869, *G. guentheri* (Herzenstein, 1892), *G. soldatovi* Berg, 1914, *G. coriparoides* Nichols, 1925, *G. rivuloides* Nichols, 1925, *G. macrocephalus* Mori, 1930, *G. tenuicorpus* Mori, 1934, *G. acutipinnatus* Men’shikov, 1939, *G. huanghensis* Lo, Yao & Chen, 1977, *G. tumenensis* (Chang, 1980), and *G. fushunensis* Xie, Li & Xie, 2007. Furthermore, the genus *Romanogobio* Bănărescu, 1961 is not distributed in China. The diagnostic keys to the tribe Gobionini in China and the species within the genus *Gobio* are given herein.

### Key to the genera of tribe Gobionini in China

**Table:** 

1	Swim bladder reduced, anterior chamber rounded, posterior chamber small and narrow, total length ~ ^1^/_2_ that of coelom	***Mesogobio* Bănărescu & Nalbant, 1973**
–	Swim bladder developed, both chambers oblong, posterior larger than anterior, total length over two-thirds that of coelom	***Gobio* Cuvier, 1816**

#### Mesogobio

Taxon classificationAnimaliaCypriniformesCyprinidae

Bănărescu & Nalbant, 1973

D9AA63FD-74F2-59F9-91AF-48CD70AF75E9

Mesogobio Bănărescu & Nalbant, 1973: 198. Type species: Mesogobio lachneri Bănărescu & Nalbant, 1973.

##### Generic characters.

Body elongated; thoracic region flattened; abdomen rounded; caudal peduncle robust, compressed laterally. Mouth inferior, horseshoe-shaped; one pair of maxillary barbels rooted at upper lip extremity. Lips thick, with tiny papillae. Eyes large, positioned in dorsal half of head; interorbital region flattened, narrow. Body covered with moderately large cycloid scales; lateral line complete, almost straight. Ventral scaleless region only before pectoral-fin base end. Anus centrally located between the posterior end of pelvic-fin base and anal-fin origin. Dorsal fin with three unbranched and seven branched rays, distal margin deeply concave; anal fin with three unbranched and six branched rays; caudal fin deeply forked, with nine branched rays in upper lobe and eight branched rays in lower lobe, lobes pointed, upper lobe slightly more pointed than lower lobe. Gill rakers rudimentary. Pharyngeal teeth "3, 5–5, 3". Swim bladder small, two chambers; anterior rounded, posterior small, narrow; total length ~ ^1^/_2_ that of coelom.

The genus *Mesogobio* is monotypic, with *M. lachneri* as its type species.

##### Distribution.

This genus is endemic to the Yalu River, between North Korea and China, occurring in both mainstream and tributaries.

#### Mesogobio lachneri

Taxon classificationAnimaliaCypriniformesCyprinidae

Bănărescu & Nalbant, 1973

875E3FF8-8869-5BA5-B0E3-403753C38E5A

Fig. 2; Table 3

Mesogobio lachneri Bănărescu & Nalbant, 1973: 199; Yue 1998: 283; Xie et al. 2007: 170; Zhang et al. 2016: 74.

##### Material examined.

ASIZB 202636–202640, • 5 specimens, 67.2–74.6 mm SL; Fengcheng City, Dandong City, Liaoning Province, from the Caohe River (40.492°N, 124.086°E, ca 90 m a.s.l.); 23 September 2014. ASIZB 202886, • 1 specimen, 69.7 mm SL; Fengcheng City, Dandong City, Liaoning Province, from the Caohe River (40.492°N, 124.086°E, ca 90 m a.s.l.); 23 September 2014. ASIZB 241488, • 1 specimen, 91.3 mm SL; Linjiang City, Baishan City, Jilin Province, from the Yalu River (41.748°N, 127.001°E, ca 360 m a.s.l.); 20 August 2023. ASIZB 246552–246554, • 3 specimens, 88.8–103.4 mm SL; Linjiang City, Baishan City, Jilin Province, from the Wudaogouhe River (41.806°N, 126.910°E, ca 340 m a.s.l.); 21 August 2023.

**Figure 2.**
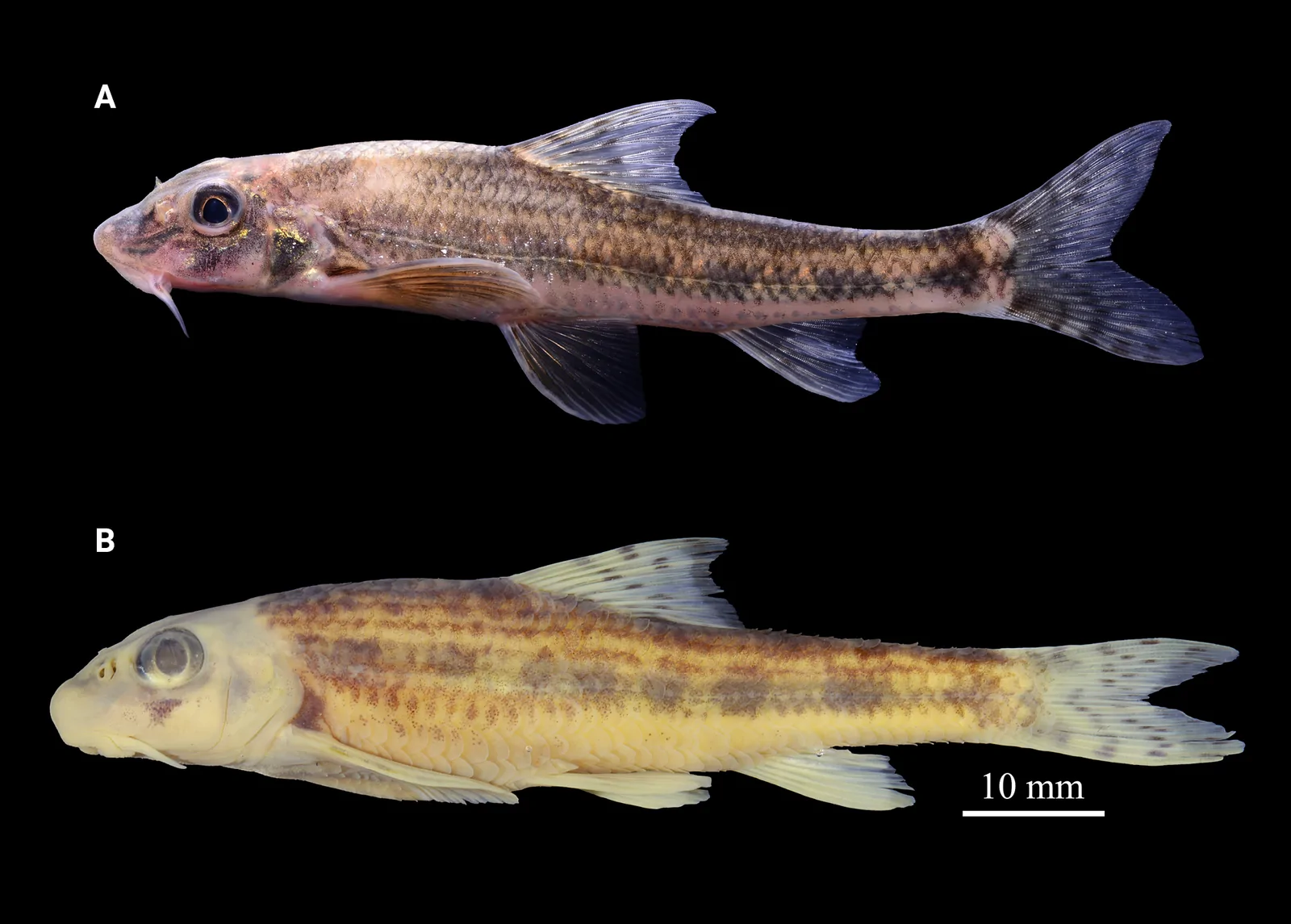
General view of *Mesogobio lachneri*. **A**. Living individual, ASIZB 241488, Linjiang City, Baishan City, Jilin Province, from the main stream of the Yalu River; **B**. ASIZB 202640, 67.2 mm SL.

**Table 3.** Morphometric measurements of *Mesogobio lachneri*.

	***Mesogobio lachneri* (*n* = 10)**		
	**Range**	**Mean**	**SD**
Dorsal-fin rays	7–8	7	
Anal-fin rays	6	6	
Pectoral-fin rays	12–14	13	
Pelvic-fin rays	7	7	
lateral line scales	40–42	42	
Scales above lateral line	5–6.5	5.5	
Scales below lateral line	3	3	
Circumpeduncular scales	12	12	
SL/mm	67.2–103.4		
**In percent of SL**			
Body depth	18.2–21.5	20.5	1.1
Head length	23.5–26.4	24.8	1.0
Dorsal-fin base length	13.6–15.9	14.9	0.7
Pectoral-fin base length	5.8–7.1	6.3	0.3
Pelvic-fin base length	4.4–5.6	5.1	0.4
Anal-fin base length	8.0–9.8	9.2	0.6
Pre-dorsal length	45.5–49.7	47.0	1.2
Pre-pectoral length	23.1–25.7	23.9	0.8
Pre-pelvic length	45.6–49.2	46.9	1.1
Pre-anal length	67.9–71.5	69.8	1.2
Post-dorsal length	55.0–58.3	56.5	1.0
Caudal peduncle length	21.2–24.6	21.9	1.1
Caudal peduncle depth	8.8–10.1	9.2	0.4
Head length/mm	16.8–24.6		
**In percent of HL**			
Head depth	56.5–65.5	62.1	3.3
Head width	60.5–72.6	68.2	3.9
Eye diameter	28.2–36.8	32.4	2.7
Interorbital width	15.3–17.9	16.8	1.0
Snout length	36.9–46.4	42.4	3.0
Barbel length	27.2–33.3	30.4	1.9

##### Diagnosis.

This species can be distinguished from other tribe Gobionini species in China by the following combination of characters: eyes large, interorbital region narrow; distal margin of dorsal fin deeply concave (vs moderate concave in other Chinese *Gobio* species); lateral-line scales 40–42 (vs > 43 in *G. tumenensis* and *G. tenuicorpus*); circumpeduncular scales 12 (vs > 12 in *G. guentheri*, *G. coriparoides*, *G. tumenensis*, *G. huanghensis*, and *G. macrocephalus*); anus centrally located between the posterior end of pelvic-fin base and anal-fin origin (vs positioned at posterior one-third in *G. guentheri*, *G. coriparoides*, and *G. soldatovi*); swim bladder small, total length ~ ^1^/_2_ that of the coelom (vs swim bladder developed, total length approximately equal to coelom in other Chinese *Gobio* species).

##### Redescription.

Body elongated, dorsal body profile rising from nostrils to dorsal-fin origin, dropping along dorsal-fin base, then gradually sloping to caudal-fin base. Maximum body depth at dorsal-fin origin, body depth 18.2%–21.5% SL. Head relatively elongated, length larger than body depth, HL 23.5%–26.4% SL; snout obtuse, with moderate concavity on top before nostrils. Mouth horseshoe-shaped, inferior, one pair of maxillary barbels rooted at extremity of upper lip; barbel relatively short, barbel length 27.2%–33.3% HL, reaching vertical through the center of the orbit. Upper lip thick, lower lip fused with throat, lips connected at mouth corners, tiny papillae present on lips, lateral lobes, and chin. Eyes large, eye diameter 28.2%–36.8% HL, positioned in dorsal half of head; interorbital region flattened and narrow, width smaller than eye diameter, interorbital width 15.3%–17.9% HL. Thoracic region flattened, abdomen rounded. Caudal peduncle robust, laterally compressed. Anus centrally located between the posterior end of pelvic-fin base and anal-fin origin.

Body covered with moderately large cycloid scales. Lateral line complete, almost straight in lateral center. Lateral line scales 40 (1 specimen), 41 (4), 42 (5); scales above lateral line 5 (1), 5.5 (8), 6.5 (1); scales below lateral line 3 (10); circumpeduncular scales 12 (10). Ventral scaleless region only before pectoral-fin base end.

Dorsal fin with three unbranched and seven (8 specimens), eight (2) branched rays; distal margin deeply concave; origin closer to snout than caudal-fin base. Pectoral fin with one unbranched and 12 (1), 13 (6), 14 (3) branched rays; adpressed pectoral-fin tip reaching anterior margin of pelvic-fin insertion. Pelvic fin with one unbranched and seven (10) branched rays; adpressed pelvic-fin tip reaching posterior to anus. Anal fin with three unbranched and six (10) branched rays; origin closer to pelvic-fin insertion than to caudal-fin base. Caudal fin deeply forked, with nine branched rays in upper lobe and eight branched rays in lower lobe, lobes pointed, upper lobe slightly more pointed than lower lobe.

Gill rakers rudimentary. Pharyngeal teeth “3, 5–5, 3". Swim bladder small, two chambers; anterior chamber rounded, posterior small and narrow; total length approximately half that of coelom.

##### Coloration in life.

Dorsal side of head and body greyish brown, mid-lateral side pale greyish brown, and ventral side grayish white. Lateral side with 7–9 black blotches. Posterior margins of scales on dorsal and lateral sides pigmented black, forming several longitudinal black stripes on upper lateral region of the body. A yellowish-green stripe with slight fluorescence present above the lateral line. One distinct black stripe between anterior orbit and snout. Fins translucent, with scattered black spots.

##### Coloration in preservation.

Dorsal side of head and body yellowish brown, mid-lateral side pale yellowish brown, and ventral side grayish white. Lateral side with 7–9 black blotches. Posterior margins of scales on dorsal and lateral sides pigmented black, forming several longitudinal black stripes on upper lateral region of the body. The fluorescent yellowish green stripe faded. One distinct black stripe between anterior orbit and snout. Fins pale, with scattered black spots.

##### Sexual dimorphism.

No significant sexual dimorphism observed.

##### Distribution.

**Figure 3.**
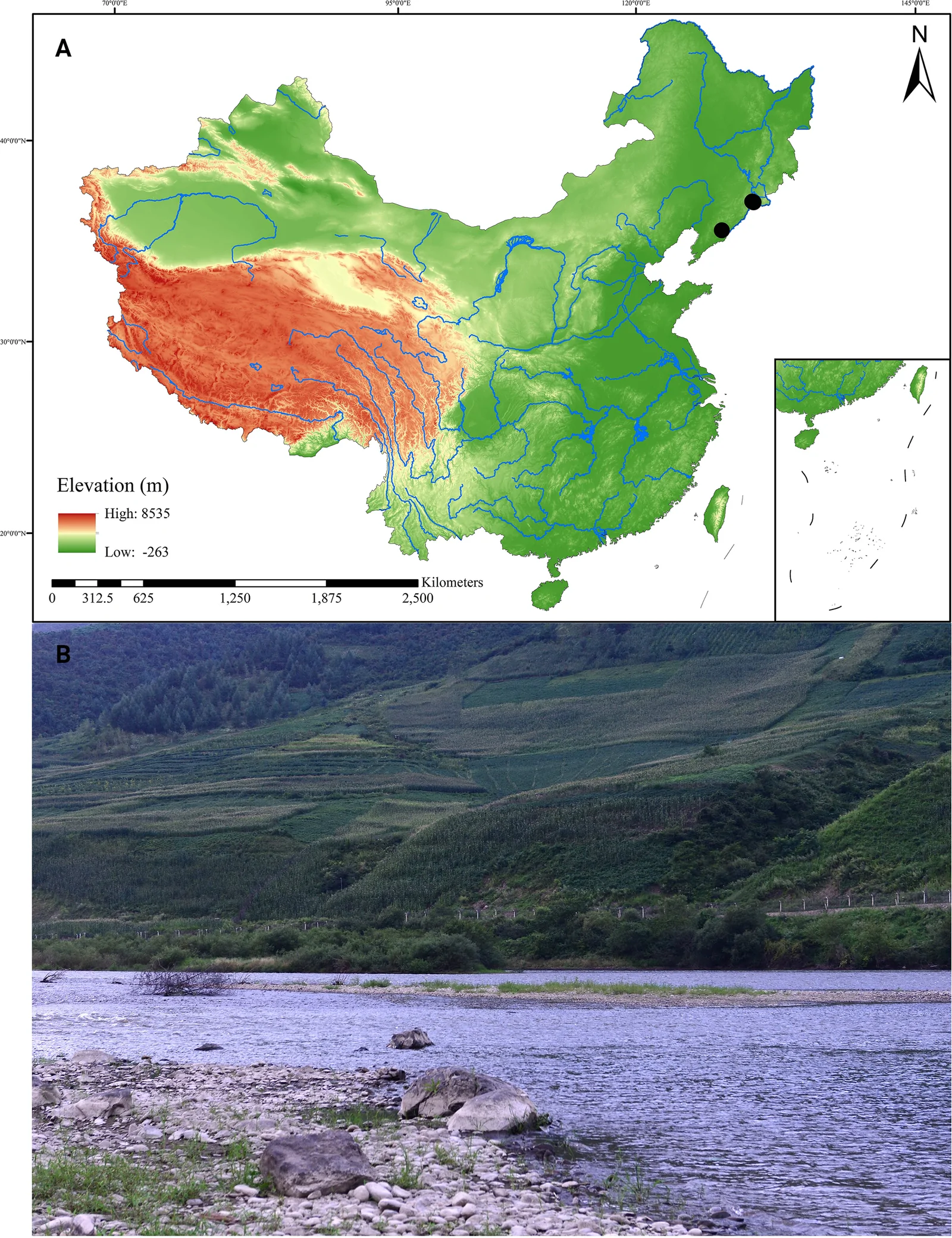
Distribution and habitat of *Mesogobio lachneri*. **A**. Geographic distribution of *M. lachneri*; **B**. The Yalu River, habitat of *M. lachneri*, photographed in Linjiang City, Baishan City, Jilin Province.

*Mesogobio lachneri* is endemic to the Yalu River drainage, occurring in both the main stream and its tributaries (Fig. 3A).

##### Habitat.

*Mesogobio lachneri* usually appears in fast-flowing water with pebble bottoms (Fig. 3B).

##### Etymology.

The name lachneri was named after Dr. Ernest A. Lachner who worked at the United States National Museum. The Chinese common name for this species is “中鮈” (Pinyin: zhōng jū).

#### Gobio

Taxon classificationAnimaliaCypriniformesCyprinidae

Cuvier, 1816

D1FDD676-09B4-5878-8F74-45E053593C35

Gobio Cuvier, 1816: 193. Type species: Cyprinus gobio Linnaeus, 1758.

##### Generic characters.

Body elongated; thoracic region flattened; abdomen rounded; caudal peduncle robust, compressed laterally. Mouth inferior, horseshoe-shaped; one pair of maxillary barbels rooted at upper lip extremity; lips thin. Eyes moderately large, positioned in dorsal half of head; interorbital region flattened. Body covered with moderately large cycloid scales; lateral line complete, almost straight. Ventral scaleless region usually before pectoral-fin base end. Dorsal fin with three unbranched and seven branched rays; anal fin with three unbranched and six branched rays; caudal fin deeply forked, with nine branched rays in upper lobe and eight branched rays in lower lobe, lobes pointed, upper lobe slightly more pointed than lower lobe. Gill rakers rudimentary. Pharyngeal teeth “3, 5–5, 3". Swim bladder developed, two chambers; both chambers oblong, posterior larger than anterior; total length approximately equal to coelom.

The genus includes 11 valid species in China.

##### Distribution.

This genus is widely distributed across Eurasia. Its westernmost limit is the Iberian Peninsula, the easternmost limit is the Russian Far East, and its southernmost limit is the Luohe River in Shaanxi Province, a tributary of the Yellow River drainage. In China, species of the genus *Gobio* are distributed across several major drainages, including the Amur, Suifen, Tumen, Yalu, Haihe, Yellow, Irtysh, and Emin river drainages, as well as some coastal rivers in northeastern China.

### Key to the species of the genus *Gobio* in China

**Table:** 

1a	Anus positioned at posterior one-third of distance from pelvic-fin base end to anal-fin origin	**2**
–	Anus centrally located between posterior end of pelvic-fin base and anal-fin origin or closer to pelvic-fin base end than to anal-fin origin	**4**
2a	Second unbranched dorsal-fin ray stiff, robust; no scales on pre-dorsal region; ventral scaleless region extends to posterior pelvic fin	***Gobio guentheri* (Herzenstein, 1892) (the Yellow River drainage)**
–	Second unbranched dorsal-fin ray soft, thin; pre-dorsal region scaled, ventral scaleless region not reaching pelvic fin base	**3**
3a	Barbel elongated, reaching from vertical position of posterior orbit to posterior preopercle margin, barbel length 30.4%–41.2% HL; circumpeduncular scales 16–20 (mean 18), fins without black spots	***Gobio coriparoides* Nichols, 1925 (the Yellow River drainage)**
–	Barbel relatively short, reaching vertical through the center of the orbit, barbel length 21.8%–32.2% HL; circumpeduncular scales 12–16 (mean 14), fins with black spots	***Gobio soldatovi* Berg, 1914 (the Haihe, Luanhe, Dalinghe, Liaohe, Yalu, and Amur river drainages)**
4a	Upper and lower jaws with horny edges	***Gobio tumenensis* (Chang, 1980) (the Tumen River drainage)**
–	Horny edges absent on upper and lower jaws	**5**
5a	Barbel elongated, reaching beyond posterior margin of preopercle, length 41.3%–58.2% HL	***Gobio huanghensis* Lo, Yao & Chen, 1977 (the Yellow River drainage)**
–	Barbel short, not reaching beyond preopercle, length 20.2%–42.1% HL	**6**
6a	Head elongated, HL 26.0%–28.8% SL (mean 27.3%), circumpeduncular scales 16–20 (mean 16)	***Gobio macrocephalus* Mori, 1930 (the Yalu, Tumen, Suifenhe and Amur river drainages)**
–	Head relatively short, HL 21.4%–27.5% SL (mean < 25.6%), circumpeduncular scales 12–16 (mean 12 or 14)	**7**
7a	Anus closer to pelvic-fin base end than to anal-fin origin, positioned at anterior ^1^/_3_ of distance from pelvic-fin base end to anal-fin origin, lateral-line scales 43–46 (mean 44)	***Gobio tenuicorpus* Mori, 1934 (the Luanhe, Liaohe and Amur river drainages)**
–	Anus centrally located between posterior end of pelvic-fin base and anal-fin origin, lateral-line scales 39–43 (except *Gobio cynocephalus*)	**8**
8a	Ventral scaleless region extends to middle of pectoral-fin base and to pelvic-fin base	***Gobio rivuloides* Nichols, 1925 (the Yellow and Haihe river drainages)**
–	Ventral scaleless region only before pectoral-fin base end	**9**
9a	Lateral line scales 39–41 (mean 40), scales above lateral line 5–6 (mean 5.5), circumpeduncular scales 12–16 (mean 12)	***Gobio fushunensis* Xie, Li & Xie, 2007 (the Yalu River drainage, and some coastal rivers in the Liaodong Peninsula e.g., the Dayanghe and Hunhe rivers)**
–	Lateral line scales 40–45 (mean 43 in *G. cynocephalus* and 41 in *G. acutipinnatus*), scales above lateral line 5.5–6.5 (mean 6.5), circumpeduncular scales 12–16 (mean 14)	**10**
10a	Eye diameter 28.6%–34.0% HL, snout length 34.5%–38.9% HL, barbel length 31.2%–40.5% HL, flank with 7–10 distinct large black blotches	***Gobio cynocephalus* Dybowski, 1869 (the Amur River drainage)**
–	Eye diameter 20.9%–29.0% HL, snout length 38.3%–44.3% HL, barbel length 25.0%–32.8% HL, flank with 9–12 small black blotches	***Gobio acutipinnatus* Men’shikov, 1939 (the Irtysh and Emin river drainages)**

#### Gobio guentheri

Taxon classificationAnimaliaCypriniformesCyprinidae

(Herzenstein, 1892)

8F677F06-FFF1-50A8-AA5F-BED1255D5EE1

Fig. 4

Acanthogobio guentheri Herzenstein, 1892: 228; Luo et al. 1977: 454; Chen et al. 1987: 105; Yue 1998: 254; Li 2015: 150; Zhang et al. 2016: 70.
Gobio guentheri : Tang et al. 2011: 103; Fang et al. 2025: 675.

##### Diagnosis.

This species can be distinguished from other *Gobio* species in China by the following combination of characters: barbel elongated, reaching beyond posterior preopercle margin (vs not extending over posterior preopercle margin in *G. coriparoides*, *G. soldatovi*, *G. macrocephalus*, *G. tenuicorpus*, *G. rivuloides*, *G. fushunensis*, *G. cynocephalus*, and *G. acutipinnatus*); circumpeduncular scales 20–24 (vs < 20 in *G. coriparoides*, *G. soldatovi*, *G. huanghensis*, *G. tenuicorpus*, *G. rivuloides*, *G. fushunensis*, *G. cynocephalus*, and *G. acutipinnatus*); anus positioned at posterior one-third of distance from pelvic-fin base end to anal-fin origin (vs centrally located or closer to pelvic-fin base end in *G. tumenensis*, *G. huanghensis*, *G. macrocephalus*, *G. tenuicorpus*, *G. rivuloides*, *G. fushunensis*, *G. cynocephalus*, and *G. acutipinnatus*). The second unbranched ray of the dorsal fin forms a stiff spine (vs soft in other Chinese *Gobio* species in); no scales on pre-dorsal region (vs scaled in other Chinese *Gobio* species); ventral scaleless region extends beyond pelvic-fin base (vs ventral scaleless region not reaching pelvic fin base in other Chinese *Gobio* species). For a detailed description see Fang et al. (2025).

**Figure 4.**
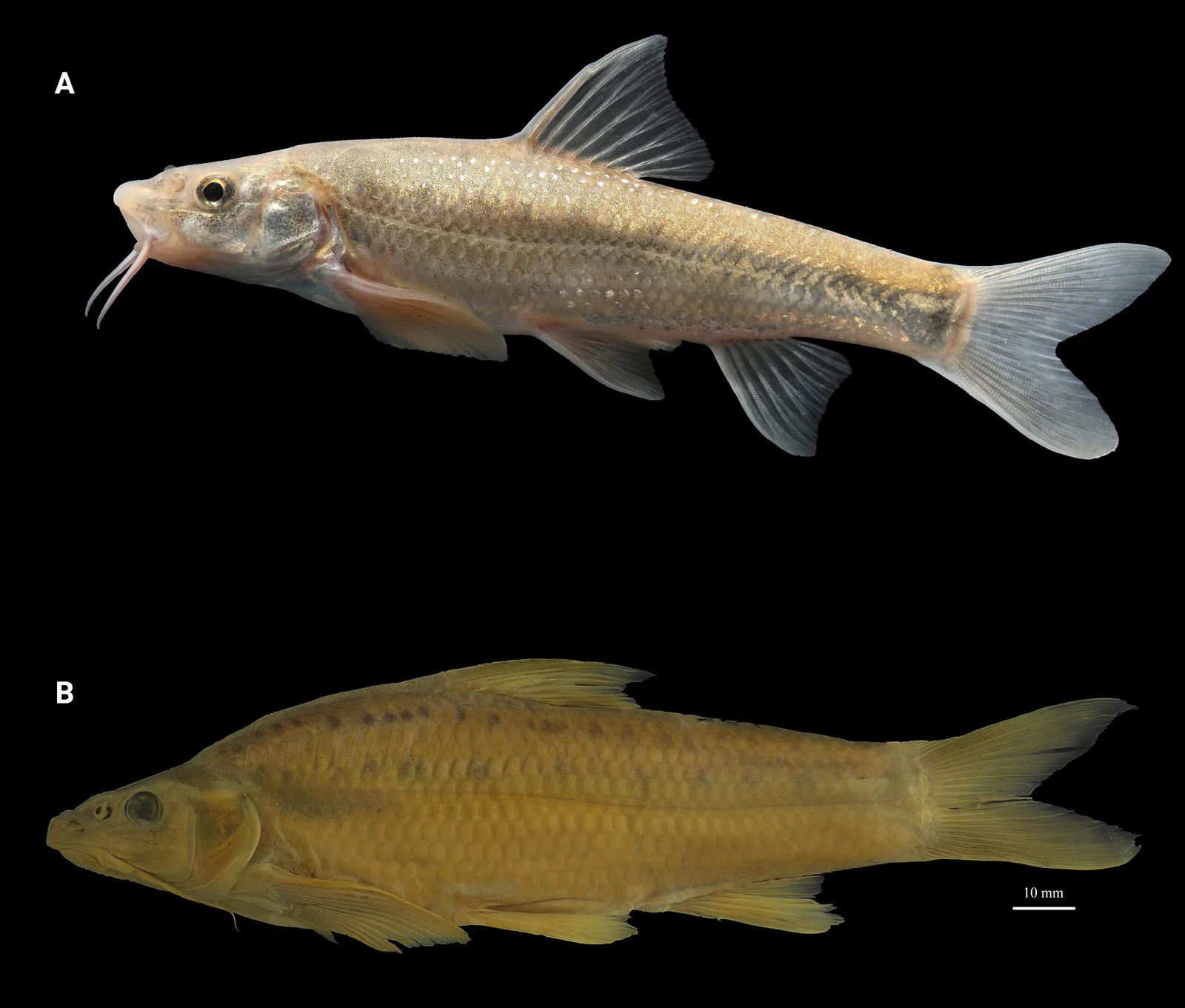
General view of *Gobio guentheri*. **A**. Living individual, ASIZB 242039, Jishishan Bonan, Dongxiang and Salar Autonomous County, Linxia Hui Autonomous Prefecture, Gansu Province, from the Yinchuanhe River; **B**. ASIZB 78482, 136.9 mm SL (Fang et al. 2025).

##### Distribution.

**Figure 5.**
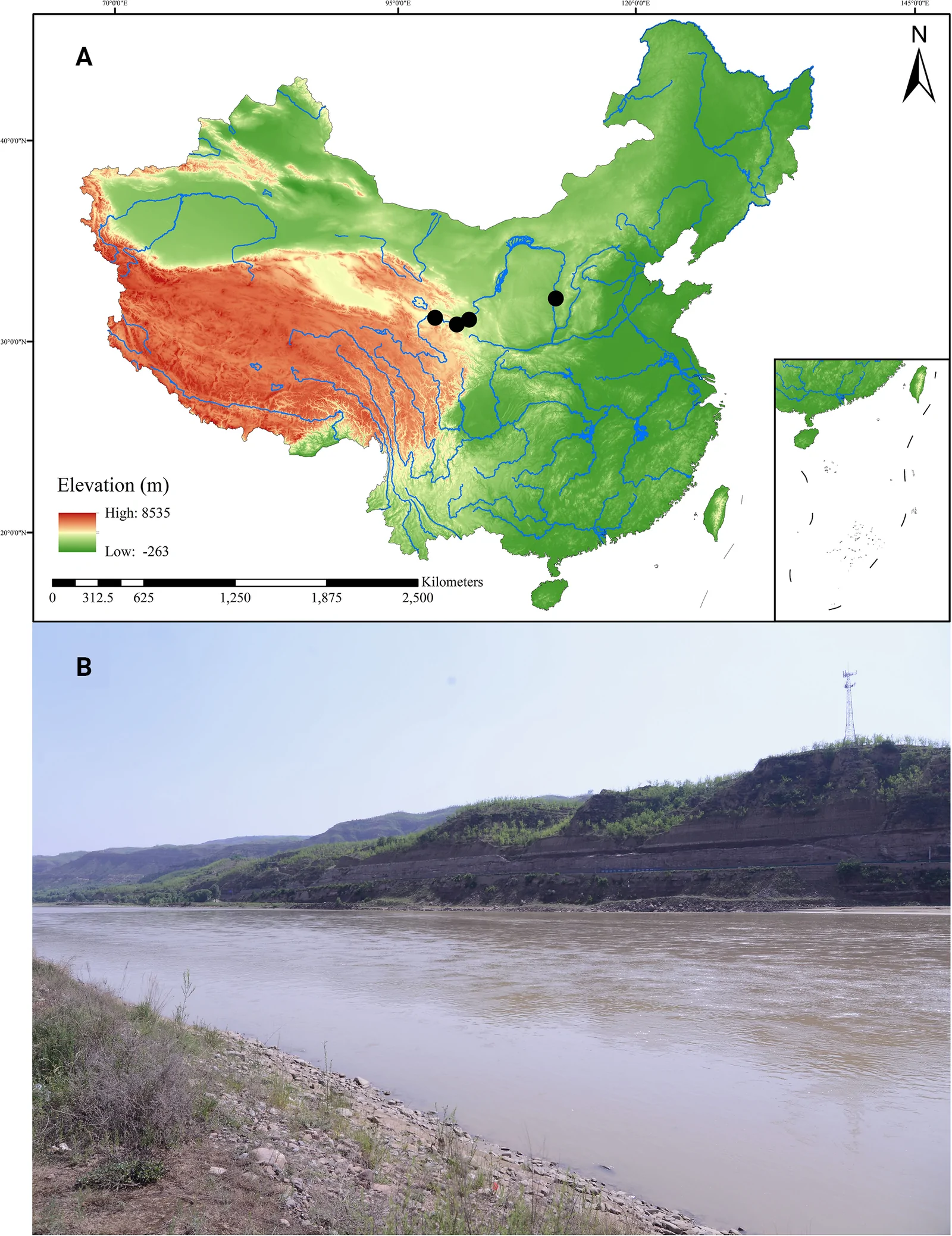
Distribution and habitat of *Gobio guentheri*. **A**. Geographic distribution of *G. guentheri*; **B**. The Yellow River, habitat of *G. guentheri*, photographed in Liulin County, Lvliang City, Shanxi Province.

*Gobio guentheri* distributed in the upper reaches of the Yellow River drainage (Fig. 5A).

##### Habitat.

*Gobio guentheri* usually appears in fast-flowing water with sandy bottoms (Fig. 5B).

##### Etymology.

The name *guentheri* was named after Dr. Albert Günther who worked at the British Museum. The Chinese common name for this species is “刺鮈” (Pinyin: cì jū).

#### Gobio coriparoides

Taxon classificationAnimaliaCypriniformesCyprinidae

Nichols, 1925

366F727E-96A2-5DED-B3AE-785DB698AEA4

Fig. 6

Gobio coriparoides Nichols, 1925: 4; Luo et al. 1977: 496; Chen et al. 1987: 116; Yue 1998: 287; Li 2015: 173; Zhang et al. 2016: 71.

##### Diagnosis.

This species can be distinguished from other *Gobio* species in China by the following combination of characters: lateral-line scales 39–41 (vs > 41 in *G. tumenensis* and *G. tenuicorpus*); circumpeduncular scales 16–20 (vs < 16 in *G. cynocephalus*); anus positioned at posterior one-third of distance from pelvic-fin base end to anal-fin origin (vs centrally located or closer to pelvic-fin base end in *G. tumenensis*, *G. huanghensis*, *G. macrocephalus*, *G. tenuicorpus*, *G. rivuloides*, *G. fushunensis*, *G. cynocephalus*, and *G. acutipinnatus*); fins without black spots (vs with black spots in *G. soldatovi*). For a detailed description see Fang et al. (2025).

**Figure 6.**
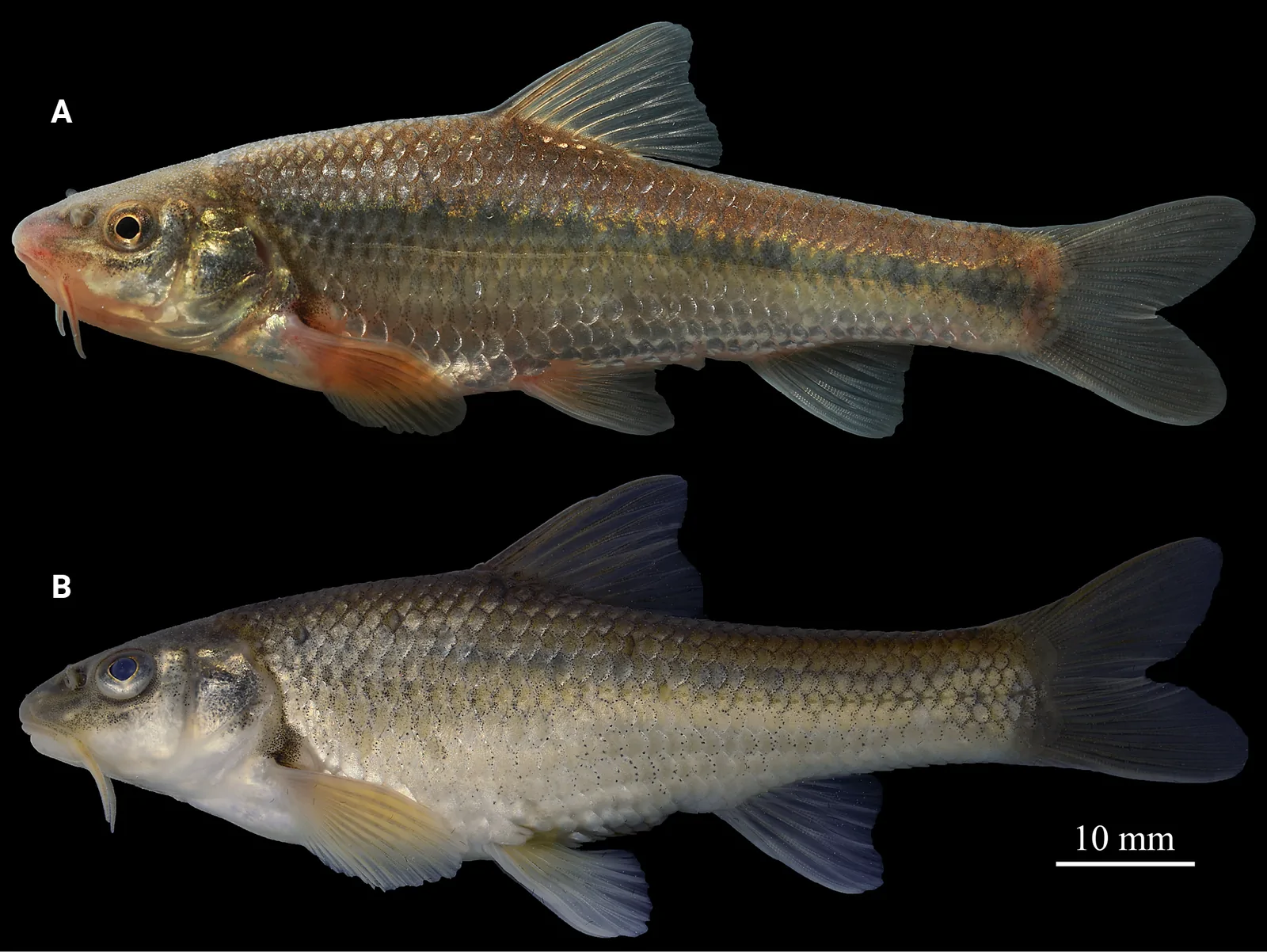
General view of *Gobio coriparoides*. **A**. Living individual, uncatalogued, Duanjiazhai Township, Jingle County, Xinzhou City, Shanxi Province, from the Fenhe River; **B**. ASIZB 242041, 68.3 mm SL (Fang et al. 2025).

##### Distribution.

**Figure 7.**
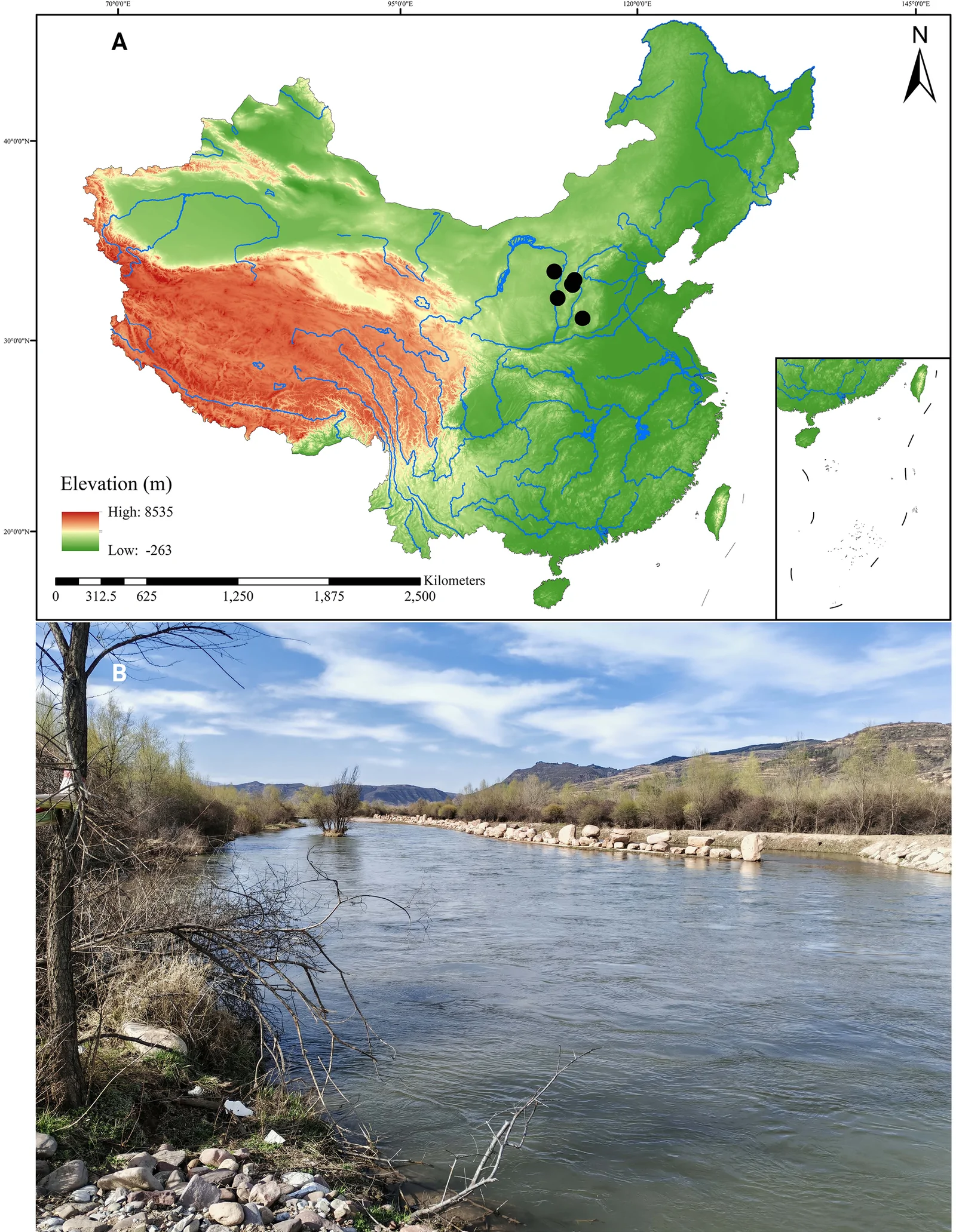
Distribution and habitat of *Gobio coriparoides*. **A**. Geographic distribution of *G. coriparoides*; **B**. The Fenhe River, a tributary of the Yellow River drainage, habitat of *G. coriparoides*, photographed in Duanjiazhai Township, Jingle County, Xinzhou City, Shanxi Province.

*Gobio coriparoides* inhabits the tributaries of the Yellow River drainage (e.g., Fenhe, Qinhe, and Kuyehe Rivers). It is rarely found in the main stream of the Yellow River (Fig. 7A).

##### Habitat.

*Gobio coriparoides* usually appears in fast-flowing water with sandy bottoms mixed with gravel and pebbles (Fig. 7B).

##### Etymology.

The name coriparoides was derived from *Coripareius*, a synonym of the genus *Coreius*, which is a genus of Gobionidae. Nichols was tempted to place this species in *Coripareius* since their teeth were not typical of *Gobio*. The Chinese common name for this species is “似铜鮈” (Pinyin: sì tóng jū).

#### Gobio soldatovi

Taxon classificationAnimaliaCypriniformesCyprinidae

Berg, 1914

8502094C-182F-5C05-868C-927A7364D8EA

Fig. 8; Table 4

Gobio gobio var. soldatovi Berg, 1914: 461.
Gobio soldatovi minulus : Nichols 1925: 3.
Gobio lingyuanensis : Mori 1934: 14; Luo et al. 1977: 495; Yue 1998: 290; Xie et al. 2007: 175; Zhang et al. 2016: 72.
Gobio soldatovi : Luo et al. 1977: 494; Yue 1998: 288; Xie et al. 2007: 175; Zhang et al. 2016: 72; Antonov et al. 2019: 92.

##### Material examined.

ASIZB 197165–197168, • 4 specimens, 62.7–76.2 mm SL; Miyun District, Beijing, from the Andamuhe River (40.650°N, 117.429°E, ca 500 m a.s.l.); 7 July 2013. ASIZB 219151–219159, • 9 specimens, 66.7–87.6 mm SL; Xinglong County, Chengde City, Hebei Province, from the Liuhe River (40.475°N, 117.567°E, ca 530 m a.s.l.); 27 September 2020. ASIZB 240001–240002, 240004, • 3 specimens, 81.0–85.2 mm SL; Chengde County, Chengde City, Hebei Province, from the Laoniuhe River (40.859°N, 118.229°E, ca 320 m a.s.l.); 9 October 2023. ASIZB 242098–242099, 248242, • 3 specimens, 62.0–84.7 mm SL; Xunke County, Heihe City, Heilongjiang Province, from the Xunhe River (49.378°N, 128.626°E, ca 130 m a.s.l.); 5 April 2023. ASIZB 248243–248244, • 2 specimens, 90.0–102.7 mm SL; Pingquan County, Chengde City, Hebei Province, from the Baohe River (40.983°N, 118.670°E, ca 490 m a.s.l.); 21 July 2023. ASIZB 248245–248251, • 7 specimens, 61.7–74.5 mm SL; Acheng District, Harbin City, Heilongjiang Province, from the Toudaohe River (45.470°N, 127.360°E, ca 300 m a.s.l.); 22 March 2024.

**Figure 8.**
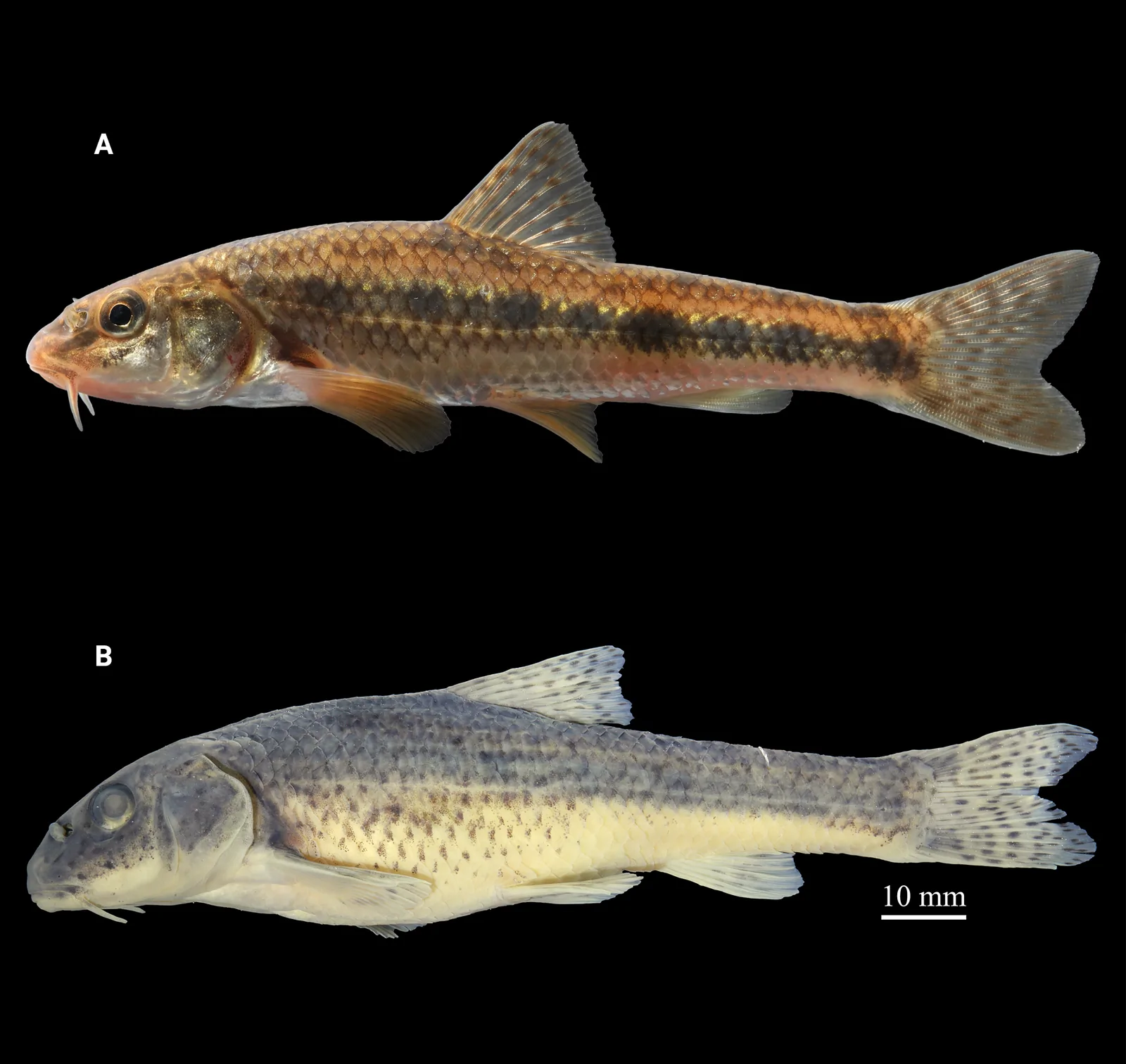
General view of *Gobio soldatovi*. **A**. Living individual, uncatalogued, Songfengshan Township, Acheng County, Harbin City, Heilongjiang Province, from the Toudaohe River; **B**. ASIZB 248243, 102.7 mm SL.

**Table 4.** Morphometric measurements of *Gobio soldatovi* and *G. lingyuanensis*.

	***Gobio soldatovi* (*n* = 10)**			***Gobio lingyuanensis* (*n* = 18)**		
	**Range**	**Mean**	**SD**	**Range**	**Mean**	**SD**
Dorsal-fin rays	7	7		7	7	
Anal-fin rays	6	6		6	6	
Pectoral-fin rays	12–14	13		13–15	14	
Pelvic-fin rays	7	7		7	7	
lateral line scales	38–40	39		38–41	40	
Scales above lateral line	5–6.5	6.5		5.5–7.5	6.5	
Scales below lateral line	3–4	4		4	4	
Circumpeduncular scales	12–16	14		14–16	14	
SL/mm	61.7–84.7			62.7–102.7		
**In percent of SL**						
Body depth	19.2–22.7	20.7	1.1	21.8–26.0	23.7	1.1
Head length	26.0–28.3	27.2	0.7	25.7–30.1	28.0	1.3
Dorsal-fin base length	12.0–15.1	13.5	1.0	12.6–15.7	14.0	0.7
Pectoral-fin base length	5.2–6.5	5.7	0.5	5.1–7.0	5.8	0.5
Pelvic-fin base length	4.2–4.7	4.5	0.2	3.9–5.1	4.3	0.3
Anal-fin base length	8.6–9.8	9.2	0.4	8.2–10.2	9.2	0.5
Pre-dorsal length	47.1–50.8	48.9	1.3	45.6–52.4	49.8	1.8
Pre-pectoral length	25.2–28.7	27.1	1.1	26.8–31.8	28.8	1.3
Pre-pelvic length	47.7–52.1	50.4	1.2	49.3–54.4	51.9	1.5
Pre-anal length	68.0–71.9	70.1	1.2	67.9–72.0	69.9	1.2
Post-dorsal length	53.7–57.8	55.1	1.3	52.4–57.8	54.5	1.4
Caudal peduncle length	20.7–23.3	21.3	0.8	18.6–23.6	22.0	1.2
Caudal peduncle depth	8.6–10.5	9.7	0.7	9.0–10.8	10.1	0.5
Head length/mm	16.6–24.0			16.6–28.8		
**In percent of HL**						
Head depth	51.5–61.8	58.5	3.4	55.4–62.5	59.1	2.0
Head width	581.–68.9	64.2	3.3	54.4–67.7	63.2	3.2
Eye diameter	24.3–30.4	27.3	1.8	24.1–27.3	25.6	1
Interorbital width	21.2–24.8	22.6	1.1	21.2–26.9	24.4	1.7
Snout length	31.9–35.3	33.5	1.1	29.4–39.6	35.8	2.4
Barbel length	21.8–32.2	26.2	3.0	22.4–30.4	25.8	2.8

##### Diagnosis.

This species can be distinguished from other *Gobio* species in China by the following combination of characters: lateral-line scales 38–41 (vs > 41 in *G. tumenensis* and *G. tenuicorpus*); circumpeduncular scales 12–16 (vs > 16 in *G. guentheri*); anus positioned at posterior one-third of distance from pelvic-fin base end to anal-fin origin (vs centrally located or closer to pelvic-fin base end in *G. tumenensis*, *G. huanghensis*, *G. macrocephalus*, *G. tenuicorpus*, *G. rivuloides*, *G. fushunensis*, *G. cynocephalus*, and *G. acutipinnatus*); fins with black spots (vs absent in *G. coriparoides*).

##### Redescription.

Body relatively abbreviated, dorsal body profile rising from nostrils to dorsal-fin origin, dropping along dorsal-fin base, then gradually sloping to caudal-fin base. Maximum body depth at dorsal-fin origin, body depth 19.2%–26.0% SL. Head elongated, length larger than body depth, HL 25.7%–30.1% SL; snout elongated, with moderate concavity on top before nostrils. Mouth horseshoe-shaped, inferior, with one pair of maxillary barbels rooted at extremity of upper lip, barbel relatively short, reaching vertical through the center of the orbit, barbel length 21.8%–32.2% HL. Upper lip thin, lower lip fused with throat, lips connected at mouth corners. Eyes positioned in dorsal half of head, eye diameter 24.1%–30.4% HL; interorbital region flattened, width smaller than eye diameter, interorbital width 21.2%–26.9% HL. Thoracic region flattened, abdomen rounded. Caudal peduncle robust, laterally compressed. Anus positioned at posterior one-third of distance from pelvic-fin base end to anal-fin origin.

Body covered with moderately large cycloid scales. Lateral line complete, almost straight in lateral center. Lateral line scales 38 (8 specimens), 39 (10), 40 (8), 41 (2); scales above lateral line 5 (1), 5.5 (3), 6 (3), 6.5 (20), 7.5 (1); scales below lateral line 3 (2), 4 (26); circumpeduncular scales 12 (1), 14 (16), 16 (11). Ventral scaleless region only before pectoral-fin base end.

Dorsal fin with three unbranched and seven (28 specimens) branched rays; distal margin concave; origin closer to snout than caudal-fin base. Pectoral fin with one unbranched and 12 (2), 13 (9), 14 (14), 15 (3) branched rays; adpressed pectoral-fin tip not reaching anterior margin of pelvic-fin insertion. Pelvic fin with one unbranched and seven (28) branched rays; adpressed pelvic-fin tip reaching posterior to anus. Anal fin with three unbranched and six (28) branched rays; origin closer to pelvic-fin insertion than to caudal-fin base. Caudal fin deeply forked, with nine branched rays in upper lobe and eight branched rays in lower lobe, lobes pointed, upper lobe slightly more pointed than lower lobe.

Gill rakers rudimentary. Pharyngeal teeth “2, 5–5, 2". Swim bladder developed, two chambers; both chambers oblong, posterior larger than anterior; total length approximately equal to coelom.

##### Coloration in life.

Dorsal side of head and body greyish green, mid-lateral side pale greyish green, and ventral side grayish white. Lateral side with 8–11 black blotches. Posterior margins of scales on dorsal and lateral sides pigmented black, forming several longitudinal black stripes on upper lateral region of the body. A yellowish-green stripe with slight fluorescence present above the lateral line. One distinct black stripe between anterior orbit and snout. Fins translucent, with scattered black spots.

##### Coloration in preservation.

Dorsal side of head and body black, mid-lateral side dark grey, and ventral side grayish white. Blotches on flank vague, merging into a dark stripe. Posterior margins of scales on dorsal and lateral sides pigmented black, forming several longitudinal black stripes on upper lateral region of the body. The fluorescent yellowish green stripe faded. One distinct black stripe between anterior orbit and snout. Fins pale, with scattered black spots.

##### Sexual dimorphism.

No significant sexual dimorphism observed.

**Figure 9.**
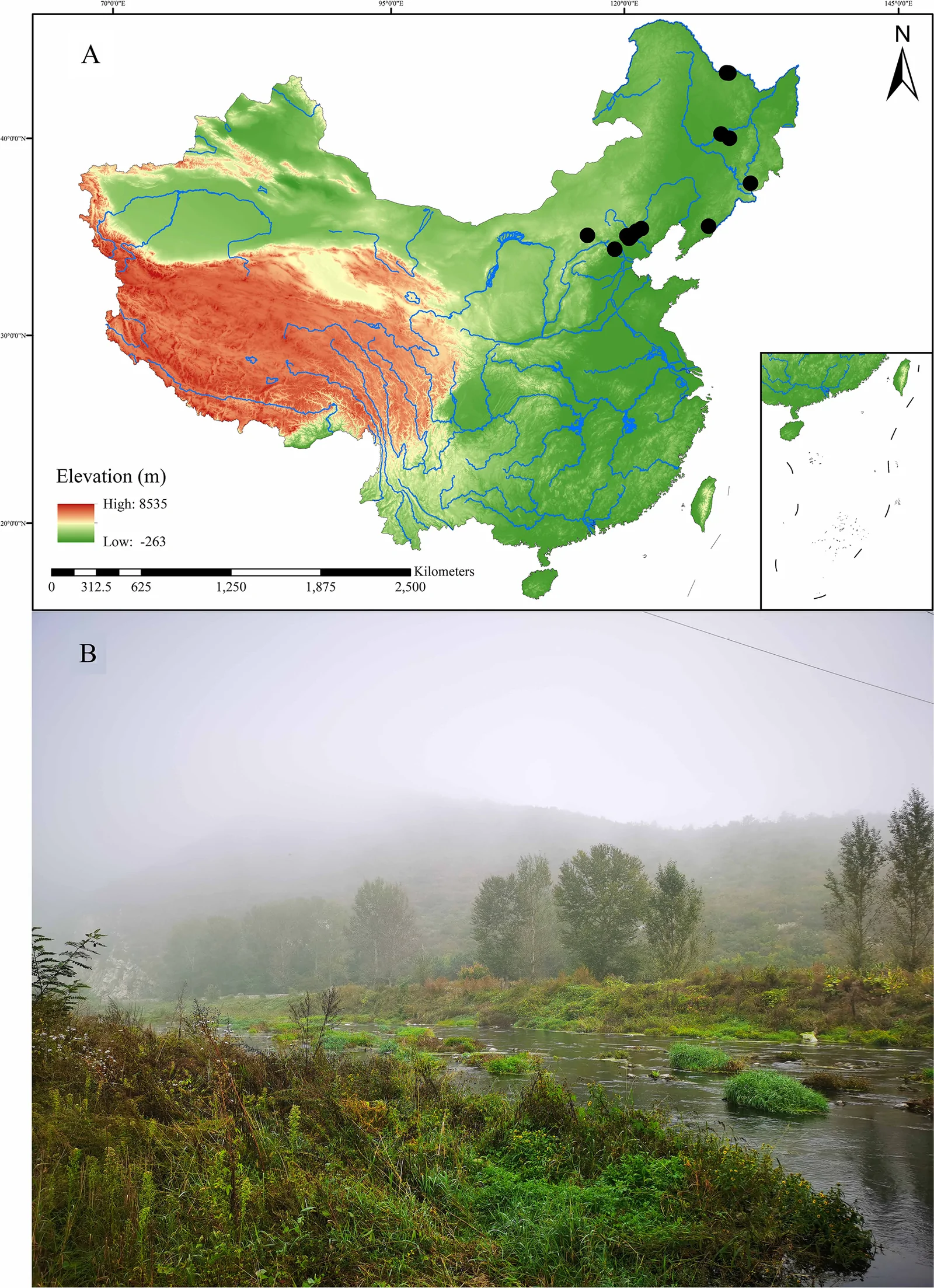
Distribution and habitat of *Gobio soldatovi*. **A**. Geographic distribution of *G. soldatovi*; **B**. The Liuhe River, a tributary of the Luanhe River drainage, habitat of *G. soldatovi*, photographed in Pinganpu Township, Xinglong County, Chengde City, Hebei Province.

**Distribution**. *Gobio soldatovi* is widely distributed in the Haihe, Luanhe, Dalinghe, Liaohe, Yalu, and Amur river drainages (Fig. 9A).

##### Habitat.

*Gobio soldatovi* usually appears in fast-flowing water with sandy bottoms mixed with gravel and pebbles (Fig. 9B).

##### Etymology.

The name soldatovi was named after B. K. Soldatov, the collector of the type specimens. The Chinese common name for this species is “高体鮈” (Pinyin: gāo tǐ jū).

##### Remarks.

*Gobio soldatovi minulus* was originally described by Nichols (1925) as a new subspecies under *G. soldatovi*. Its type locality is Kweihwa, Shansi (now Hohhot City, Inner Mongolia Autonomous Region). Yue (1998) listed *G. soldatovi minulus* as a junior synonym of *G. lingyuanensis*, but did not provide any specimen comparison information. We examined the holotype AMNH 8417 and paratypes AMNH 10573 (10 specimens) of *G. soldatovi minulus*. These type materials have 38–40 lateral line scales; 5.5–6.5 scales above lateral line; 3–4 scales below lateral line; 14–16 circumpeduncular scales; barbel relatively short, reaching vertical through the center of the orbit; anus closer to anal-fin origin than pelvic-fin base end. Since all of the characters described above agree with those of *G. soldatovi*, *G. soldatovi minulus* should be considered a junior synonym of *G. soldatovi*.

**Figure 10.**
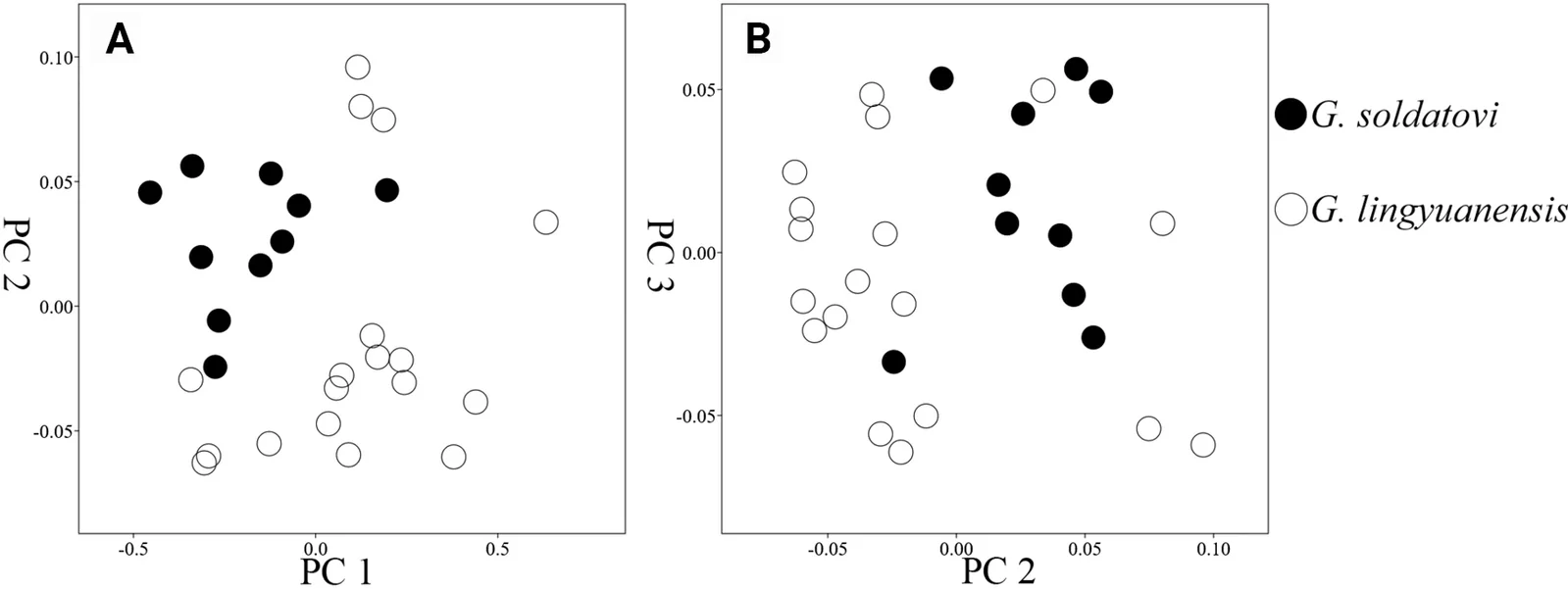
**A**. Scatterplot of PC1 against PC2; **B**. PC2 against PC3 extracted from 19 morphometric data of *Gobio soldatovi* and *G. lingyuanensis*.

Mori (1934) originally described *Gobio lingyuanensis* based on the specimens collected from Te-ling-ho, Ling-yuan (Lingyuan City, Chaoyang City, Liaoning Province, the Dalinghe River drainage) and Hsing-lung-shan (Xinglong County, Chengde City, Hebei Province, the Luanhe River drainage). According to the original description, *G. lingyuanensis* is similar to *G. soldatovi*, but the former has a longer head and a shorter caudal peduncle; these characters, however, cannot clearly distinguish these two species. Some taxonomic keys proposed in previous studies (e.g., Luo et al. 1977 and Yue 1998) were also not able to clearly distinguish these two species from each other. Based on the distribution stated in the original descriptions, we identified the specimens that were collected from the Luanhe and Haihe river drainages as *G. lingyuanensis*, while those from the Amur River drainage as *G. soldatovi*. We measured these newly collected specimens, and the results are presented in Table 4. Mann–Whitney U tests with Bonferroni correction were performed to assess differences in morphometric characters between the two species. Only body depth showed a significant difference after correction (adjusted *p* = 0.000768), whereas the remaining characters showed no significant differences. We further conducted principal component analysis (PCA) to evaluate whether the two species could be separated based on overall morphology. The PCA results are presented in Fig. 10 and the loadings are shown in Table 5. The first three components contributed 94.5% of the variance. Neither principal component 1 (PC 1) versus PC 2 nor PC 2 versus PC 3 showed clear separation between *G. soldatovi* and *G. lingyuanensis*. These results suggest that the two species are not clearly differentiated based on the morphometric characters examined.

**Table 5.** Loadings on the first three principal components extracted from morphometric data of *Gobio soldatovi* and *G. lingyuanensis*. Bold values indicate relatively high loadings.

	**PC 1**	**PC 2**	**PC 3**
Standard length	0.206	0.218	0.067
Body depth	0.266	–0.270	–0.088
Head length	0.236	0.008	0.133
Head depth	0.232	–0.088	0.010
Head width	0.223	–0.244	0.253
Dorsal-fin base length	0.232	–0.010	–0.053
Pectoral-fin base length	0.216	0.197	**–0.844**
Pelvic-fin base length	0.226	**0.500**	0.232
Anal-fin base length	0.211	–0.013	0.060
Caudal peduncle length	0.197	0.226	–0.030
Caudal peduncle width	0.242	–0.038	–0.096
Eye diameter	0.175	0.153	0.123
Interorbital width	0.268	**–0.386**	–0.180
Snout length	**0.290**	**–0.423**	0.165
Pre-dorsal length	0.224	0.004	0.099
Pre-pectoral length	0.248	–0.028	–0.104
Pre-pelvic length	0.216	0.095	0.079
Pre-anal length	0.212	0.214	0.081
Post-dorsal length	0.214	0.253	0.122

The phylogenetic tree shows that *Gobio lingyuanensis* is nested within *G. soldatovi* (Fig. 1). The genetic distance (Table 2) between *G. lingyuanensis* and *G. soldatovi* is 0.0038, which is comparable to the intraspecific distances of *G. soldatovi* (0.0023) and *G. lingyuanensis* (0.0053). This result further supports *G. lingyuanensis* to be a junior synonym of *G. soldatovi*.

#### Gobio tumenensis

Taxon classificationAnimaliaCypriniformesCyprinidae

(Chang, 1980)

0C1C0776-F481-55A6-8EFD-82998ADD6D52

Fig. 11; Table 6

Mesogobio tumenensis Chang (in Zheng et al.), 1980: 43; Yue 1998: 284; Xie et al. 2007: 171; Zhang et al. 2016: 74.
Gobio tumenensis : Tang et al. 2011: 121.

##### Material examined.

ASIZB 62908, ***holotype***, 153.9 mm SL; Helong County, Yanbian Chaoxianzu Autonomous Prefecture, Jilin Province, from the Tumen River; 4 June 1977. ASIZB 62912, 62914, 62916, 62918, 62924, ***paratypes***, • 5 specimens, 84.2–112.2 mm SL; Wangqing County, Yanbian Chaoxianzu Autonomous Prefecture, Jilin Province, from the Gayahe River; 25 June 1977. ASIZB 61666, • 1 specimen, 132.7 mm SL; Hunchun County, Yanbian Chaoxianzu Autonomous Prefecture, Jilin Province, from the Hunchunhe River (42.895°N, 130.781°E, ca 200 m a.s.l.). ASIZB 238333, • 1 specimen, 85.5 mm SL; Hunchun County, Yanbian Chaoxianzu Autonomous Prefecture, Jilin Province, from the Mijianghe River (42.995°N, 130.144°E, ca 60 m a.s.l.); 2 September 2022. ASIZB 238481, 238486, • 2 specimens, 74.6–109.6 mm SL; Tumen County, Yanbian Chaoxianzu Autonomous Prefecture, Jilin Province, from the Gayahe River (43.100°N, 129.788°E, ca 90 m a.s.l.); 3 September 2022. ASIZB 248201, 248900, • 2 specimens, 70.2–96.1 mm SL; Antu County, Yanbian Chaoxianzu Autonomous Prefecture, Jilin Province, from the Buerhatonghe River (43.019°N, 129.039°E, ca 300 m a.s.l.); 24 August 2023.

**Figure 11.**
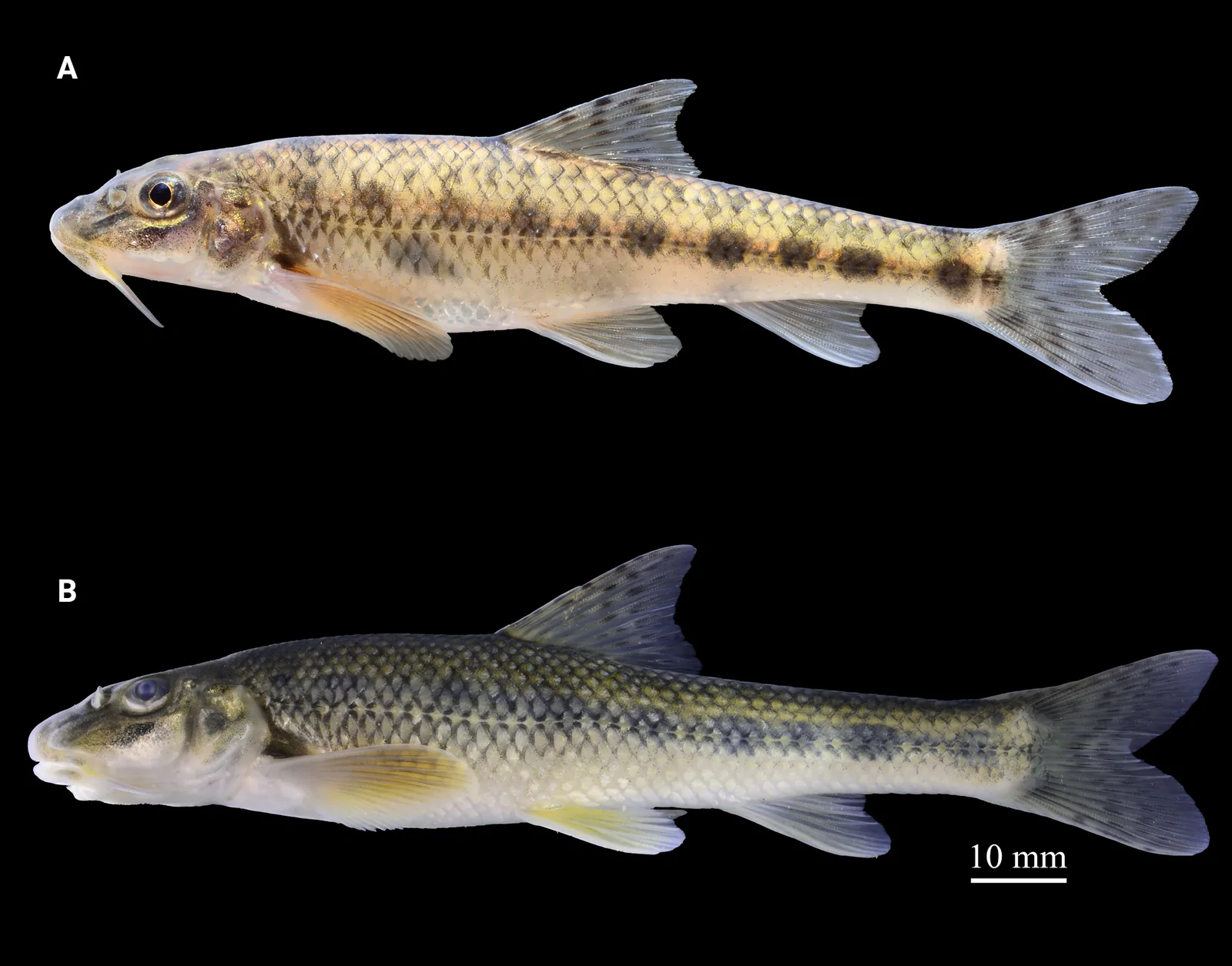
General view of *Gobio tumenensis*. **A**. Living individual, uncatalogued, Antu County, Yanbian Chaoxianzu Autonomous Prefecture, Jilin Province, from the Bu’erhatonghe River; **B**. ASIZB 248900, 96.1 mm SL.

**Table 6.** Morphometric measurements of *Gobio tumenensis*.

	***Gobio tumenensis* (*n* = 12)**		
	**Range**	**Mean**	**SD**
Dorsal-fin rays	7	7	
Anal-fin rays	6	6	
Pectoral-fin rays	13–16	14	
Pelvic-fin rays	7	7	
lateral line scales	43–46	44	
Scales above lateral line	7.5–8.5	7.5	
Scales below lateral line	4–5	5	
Circumpeduncular scales	16–20	18	
SL/mm	70.2–153.9		
**In percent of SL**			
Body depth	18.5–22.6	19.8	1.1
Head length	22.7–25.0	23.9	0.6
Dorsal-fin base length	12.6–15.3	13.7	0.7
Pectoral-fin base length	4.8–6.5	5.6	0.5
Pelvic-fin base length	3.9–5.1	4.5	0.4
Anal-fin base length	7.9–9.9	8.7	0.5
Pre-dorsal length	46.1–50.5	48.5	1.0
Pre-pectoral length	22.7–25.6	23.9	0.8
Pre-pelvic length	46.5–52.2	49.5	1.4
Pre-anal length	69.4–73.4	71.4	1.3
Post-dorsal length	53.8–57.5	55.2	1.2
Caudal peduncle length	19.9–22.5	21.1	0.9
Caudal peduncle depth	8.1–9.7	9.0	0.4
Head length/mm	17.3–35.7		
**In percent of HL**			
Head depth	55.5–61.0	57.7	1.6
Head width	57.4–69.1	62.1	3.5
Eye diameter	23.4–28.9	26.8	1.5
Interorbital width	17.5–21.8	19.2	1.3
Snout length	38.6–48.8	44.0	2.7
Barbel length	27.8–47.3	39.2	5.3

##### Diagnosis.

This species can be distinguished from other *Gobio* species in China by the following combination of characters: barbel elongated, extending over posterior preopercle margin (vs not extending over posterior preopercle margin in *G. coriparoides*, *G. soldatovi*, *G. macrocephalus*, *G. tenuicorpus*, *G. rivuloides*, *G. fushunensis*, *G. cynocephalus*, and *G. acutipinnatus*); lips thick, upper and lower jaws with horny edges (vs without horny jaws in other Chinese *Gobio* species); tiny papillae on lips, lateral lobes, and chin (vs absent in other *Gobio* species except *G. huanghensis*); lateral-line scales 43–46 (vs < 43 in *G. coriparoides*, *G. soldatovi*, and *G. fushunensis*); circumpeduncular scales 16–20 (vs < 16 in *G. cynocephalus*); anus centrally located between the posterior end of pelvic-fin base and anal-fin origin (vs positioned at posterior one-third in *G. guentheri*, *G. coriparoides*, and *G. soldatovi*).

##### Redescription.

Body elongated, dorsal body profile rising from nostrils to dorsal-fin origin, dropping along dorsal-fin base, then gradually sloping to caudal-fin base. Maximum body depth at dorsal-fin origin, body depth 18.5%–22.6% SL. Head elongated, length larger than body depth, HL 22.7%–25.0% SL; snout elongated, with moderate concavity on top before nostrils. Mouth horseshoe-shaped, inferior; upper and lower jaws with horny edges; one pair of maxillary barbels rooted at extremity of upper lip, barbel elongated, barbel length 27.8%–47.3% HL, extending over posterior preopercle margin. Upper lip thick, lower lip fused with throat, lips connected at mouth corners, tiny papillae on lips, lateral lobes, and chin. Eyes positioned in dorsal half of head, eye diameter 23.4%–28.9% HL; interorbital region flattened, width smaller than eye diameter, interorbital width 17.5%–21.8% HL. Thoracic region flattened, abdomen rounded. Caudal peduncle robust, laterally compressed. Anus centrally located between the posterior end of pelvic-fin base and anal-fin origin.

Body covered with moderately large cycloid scales. Lateral line complete, almost straight in lateral center. Lateral line scales 43 (3 specimens), 44 (6), 45 (1), 46 (2); scales above lateral line 7.5 (10), 8 (1), 8.5 (1); scales below lateral line 4 (3), 5 (9); circumpeduncular scales 16 (4), 18 (7), 20 (1). Ventral scaleless region only before pectoral-fin base end.

Dorsal fin with three unbranched and seven (12 specimens) branched rays; distal margin concave; origin closer to snout than caudal-fin base. Pectoral fin with one unbranched and 13 (4), 14 (5), 15 (2), 16 (1) branched rays; adpressed pectoral-fin tip not reaching anterior margin of pelvic-fin insertion. Pelvic fin with one unbranched and seven (12) branched rays; adpressed pelvic-fin tip reaching posterior to anus. Anal fin with three unbranched and six (12) branched rays; origin closer to pelvic-fin insertion than to caudal-fin base. Caudal fin deeply forked, with nine branched rays in upper lobe and eight branched rays in lower lobe, lobes pointed, upper lobe slightly more pointed than lower lobe.

Gill rakers rudimentary. Pharyngeal teeth “3, 5–5, 3". Swim bladder relatively developed, two chambers; anterior chamber oblong, posterior narrow, posterior larger than anterior; total length ~ ^2^/_3_ of coelom.

##### Coloration in life.

Dorsal side of head and body yellowish brown, mid-lateral side pale yellowish brown, and ventral side grayish white. Lateral side with 9–11 black blotches. Posterior margins of scales on dorsal and lateral sides pigmented black. A yellowish-green stripe with slight fluorescence present above the lateral line. One distinct black stripe between anterior orbit and snout. Fins translucent, dorsal fin, pectoral fin, and caudal fin with black spots; pelvic fin and anal fin with slightly black pigment.

##### Coloration in preservation.

Dorsal side of head and body yellowish brown, mid-lateral side pale yellowish brown, and ventral side grayish white. Lateral side with 9–11 black blotches. Posterior margins of scales on dorsal and lateral sides pigmented black. The fluorescent yellowish green stripe faded. One distinct black stripe between anterior orbit and snout. Fins pale, dorsal fin, pectoral fin, and caudal fin with black spots; pelvic fin and anal fin with slightly black pigment.

##### Sexual dimorphism.

No significant sexual dimorphism observed.

##### Distribution.

**Figure 12.**
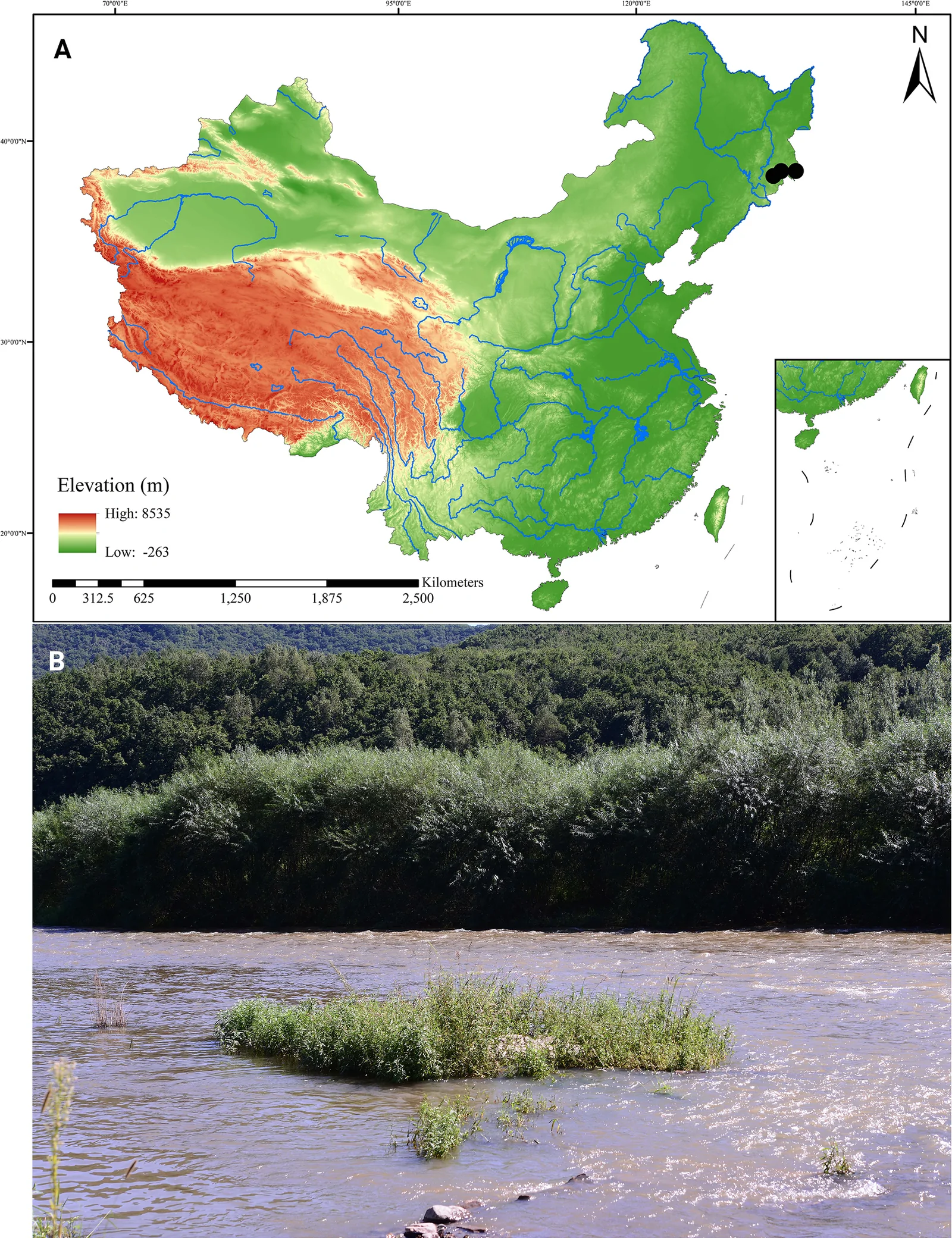
Distribution and habitat of *Gobio tumenensis*. **A**. Geographic distribution of *G. tumenensis*; **B**. The Bu’erhatonghe River, a tributary of the Tumen River drainage, habitat of *G. tumenensis*, photographed in Antu County, Yanbian Chaoxianzu Autonomous Prefecture, Jilin Province.

*Gobio tumenensis* is endemic to the Tumen River drainage, occurring in both the main stream and its tributaries (Fig. 12A).

##### Habitat.

*Gobio tumenensis* usually appears in fast-flowing water with sandy bottoms mixed with gravel and pebbles (Fig. 12B).

##### Etymology.

The name *tumenensis* refers to the Tumen River, the habitat of this species. The Chinese common name for this species is “图们鮈” (Pinyin: tú mén jū).

##### Remarks.

We found that one of the paratype specimens of *Gobio tumenensis* (ASIZB 62922) exhibits some morphological differences from other specimens. This specimen has a more elongated head (length 25.0% of body length) and shorter barbel which reaches the vertical through the center of the orbit, with the length 26.2% of head length. After a detailed morphological comparison, we confirmed that this specimen is actually *G. macrocephalus*.

*Gobio tumenensis* was placed in the genus *Mesogobio* in its original description (Zheng et al. 1980). Yang et al. (2006) constructed a phylogenetic tree using *cytb* sequences and found that the *M. tumenensis* was nested within the genus *Gobio*. Tang et al. (2011) further explored the phylogeny of Gobioninae, and their result also supported the placement of *M. tumenensis* within *Gobio*. To maintain the monophyly of the genus *Gobio*, they recommended reclassifying *M. tumenensis* as a member of *Gobio*, with the valid combination being *Gobio tumenensis*. Our phylogenetic tree also supports this result: *G. tumenensis* is nested within the genus *Gobio* and forms a sister group with *G. cynocephalus* (Fig. 1).

**Figure 13.**
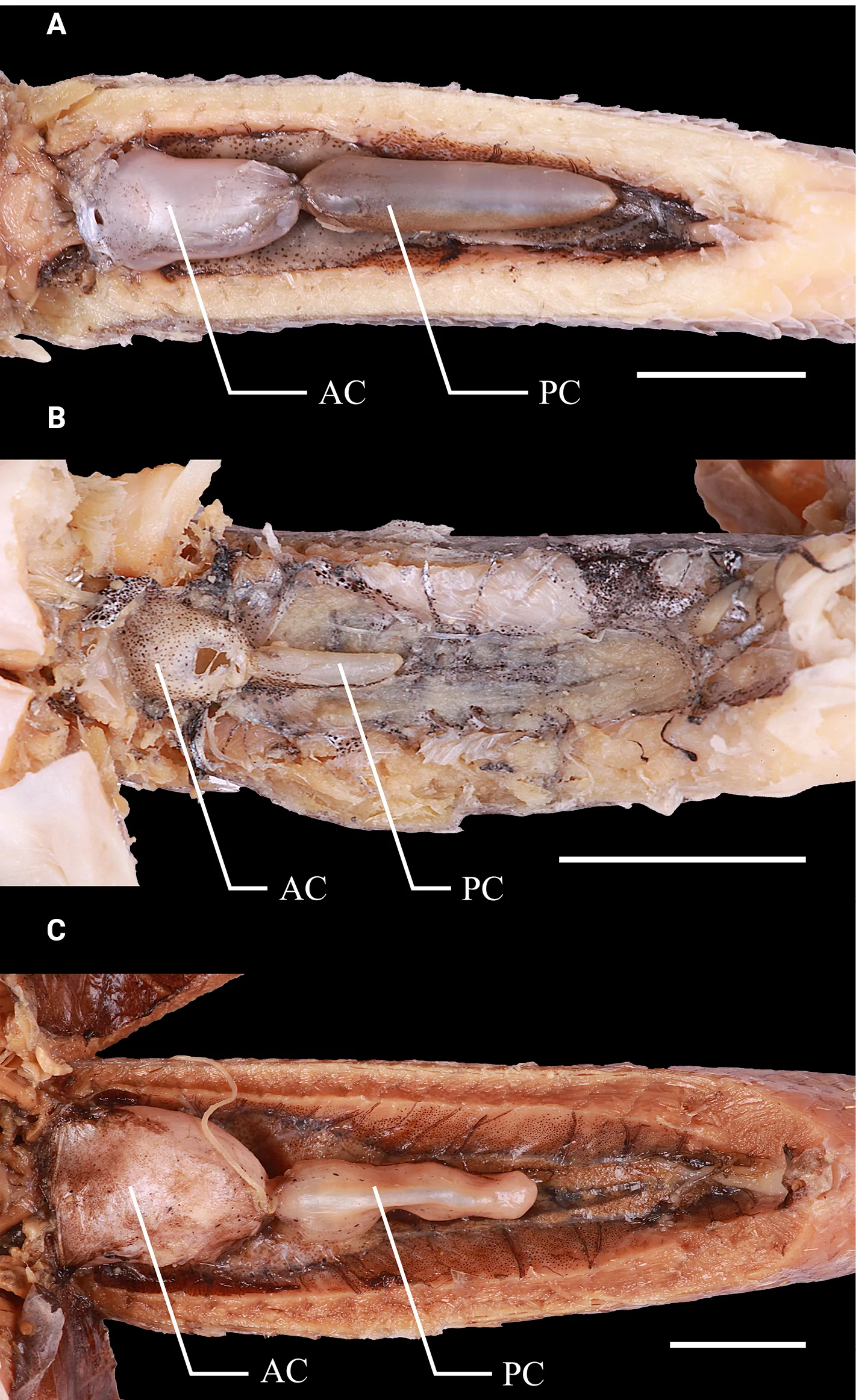
Swim bladder of species in the tribe Gobionini. **A**. *Gobio acutipinnatus*; **B**. *Mesogobio lachneri*; **C**. *G. tumenensis*. AC = anterior chamber; PC = posterior chamber. Scale bar: 10 mm.

We further explored the morphological differences between the genera *Mesogobio* and *Gobio*. We found that the swim bladder of *Mesogobio* is reduced, with its total length approximately half that of the coelom; in contrast, the swim bladder of *Gobio* is developed, with its total length roughly equal to that of the coelom. The swim bladder of *G. tumenensis* is relatively developed, with its total length approximately two-thirds that of the coelom (Fig. 13). This morphological character further supports that *G. tumenensis* should belong to the genus *Gobio*.

#### Gobio huanghensis

Taxon classificationAnimaliaCypriniformesCyprinidae

Lo, Yao & Chen, 1977

7ADC14DC-6B8A-5BD7-96EA-90BB5EB70093

Fig. 14

Gobio huanghensis Lo, Yao & Chen, 1977: 496; Chen et al. 1987: 117; Yue 1998: 299; Li 2015: 180; Zhang et al. 2016: 72.
Gobio tchangi : Li 2015: 178.

##### Diagnosis.

This species can be distinguished from other *Gobio* species in China by the following combination of characters: barbel elongated, reaching beyond posterior preopercle margin (vs not extending over posterior preopercle margin in *G. coriparoides*, *G. soldatovi*, *G. macrocephalus*, *G. tenuicorpus*, *G. rivuloides*, *G. fushunensis*, *G. cynocephalus*, and *G. acutipinnatus*); tiny papillae on lips, lateral lobes, and chin (vs absent in other *Gobio* species except *G. tumenensis*); circumpeduncular scales 14–16 (vs 20–24 in *G. guentheri*); anus centrally located between pelvic-fin base end and anal-fin origin (vs positioned at posterior one-third in *G. guentheri*, *G. coriparoides*, and *G. soldatovi*). For a detailed description see Fang et al. (2025).

**Figure 14.**
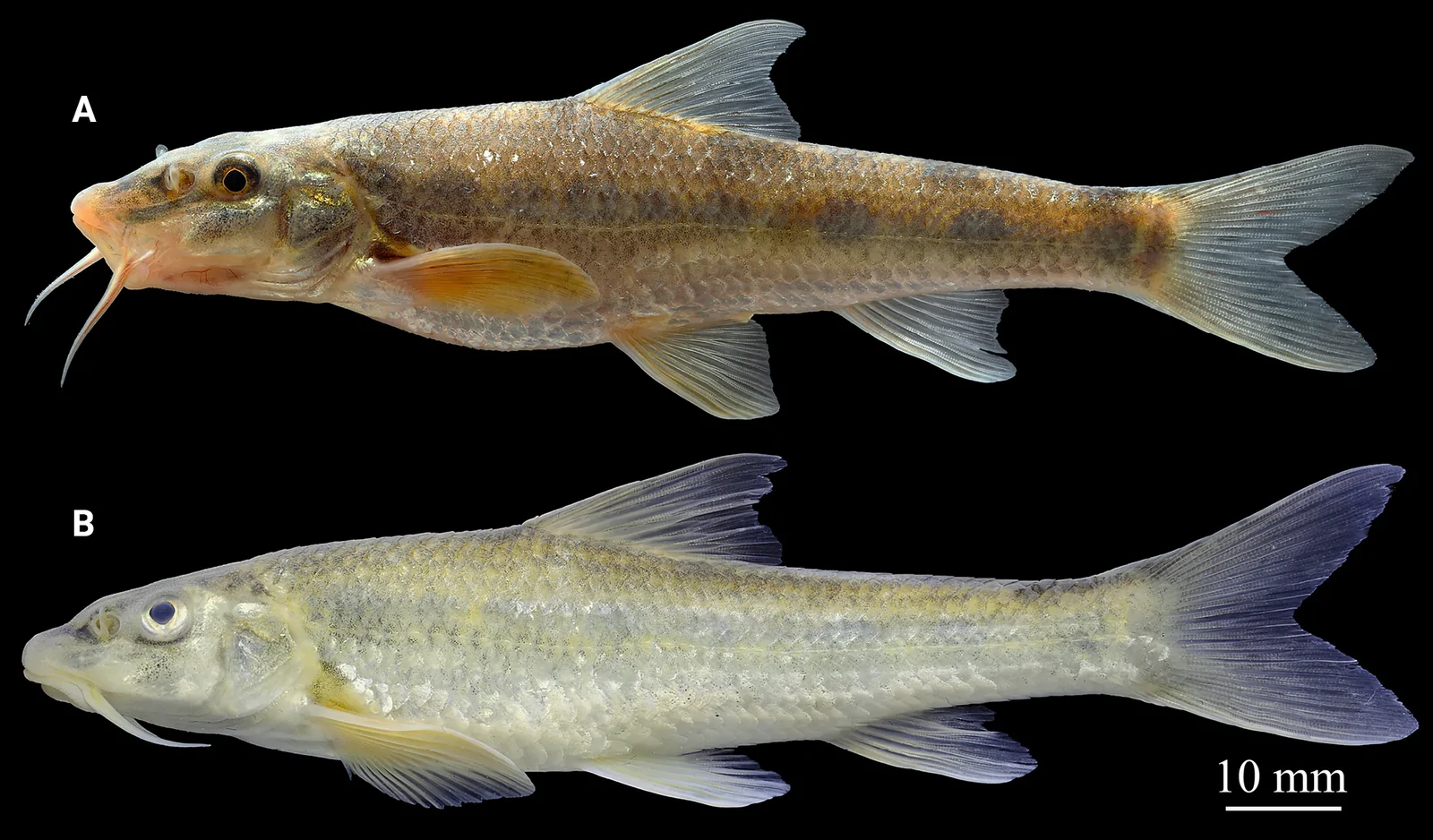
General view of *Gobio huanghensis*. **A**. Living individual, uncatalogued, Fengjiachuan Township, Baode County, Xinzhou City, Shanxi Province, from the Yellow River; **B**. ASIZB 242040, 88.6 mm SL (Fang et al. 2025).

##### Distribution.

**Figure 15.**
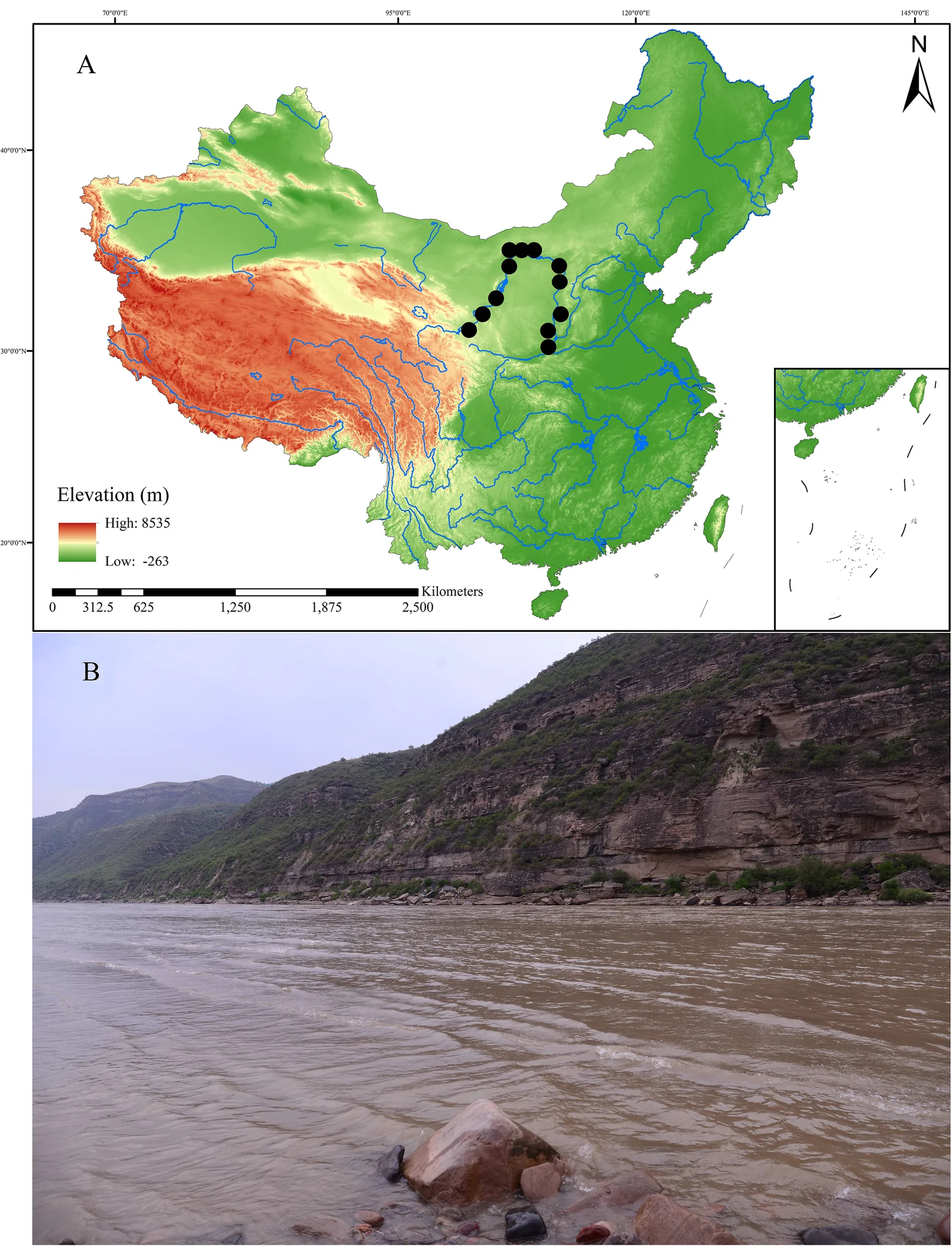
Distribution and habitat of *Gobio huanghensis*. **A**. Geographic distribution of *G. huanghensis*; **B**. The Yellow River, habitat of *G. huanghensis*, photographed in Daning County, Linfen City, Shanxi Province.

*Gobio huanghensis* inhabits the main stream of the Yellow River drainage (Fig. 15A).

##### Habitat.

*Gobio huanghensis* usually appears in fast-flowing water with sandy bottoms and high turbidity, which is caused by fine sediments (Fig. 15B).

##### Etymology.

The name huanghensis refers to the Yellow River, the habitat of this species. The Chinese common name for this species is “黄河鮈” (Pinyin: huáng hé jū).

#### Gobio macrocephalus

Taxon classificationAnimaliaCypriniformesCyprinidae

Mori, 1930

86F51CC9-5838-5289-85E1-8424A7181713

Fig. 16; Table 7

Gobio gobio macrocephalus Mori, 1930: 8; Luo et al. 1977: 498.
Gobio macrocephalus : Zheng et al. 1980: 47; Yue 1998: 293; Xie et al. 2007: 176; Zhang et al. 2016: 72.

##### Material examined.

ASIZB 241451–241455, 241495–241496, • 7 specimens, 88.2–119.7 mm SL; Tonghua County, Tonghua City, Jilin Province, from the Hunjiang River (41.573°N, 125.863°E, ca 350 m a.s.l.). ASIZB 241491–241493, • 3 specimens, 69.0–96.7 mm SL; Antu County, Yanbian Chaoxianzu Autonomous Prefecture, Jilin Province, from the Buerhatonghe River (43.019°N, 129.039°E, ca 300 m a.s.l.); 24 August 2023. ASIZB 241494, • 1 specimen, 69.3 mm SL; Dongning County, Mudanjiang City, Heilongjiang Province, from the Suifenhe River (44.071°N, 131.130°E, ca 100 m a.s.l.); 27 August 2023. ASIZB 249411–249412, • 2 specimens, 93.3–98.9 mm SL; Wangqing County, Yanbian Chaoxianzu Autonomous Prefecture, Jilin Province, from the Gayahe River (44.365°N, 129.643°E, ca 300 m a.s.l.); 26 August 2023.

**Figure 16.**
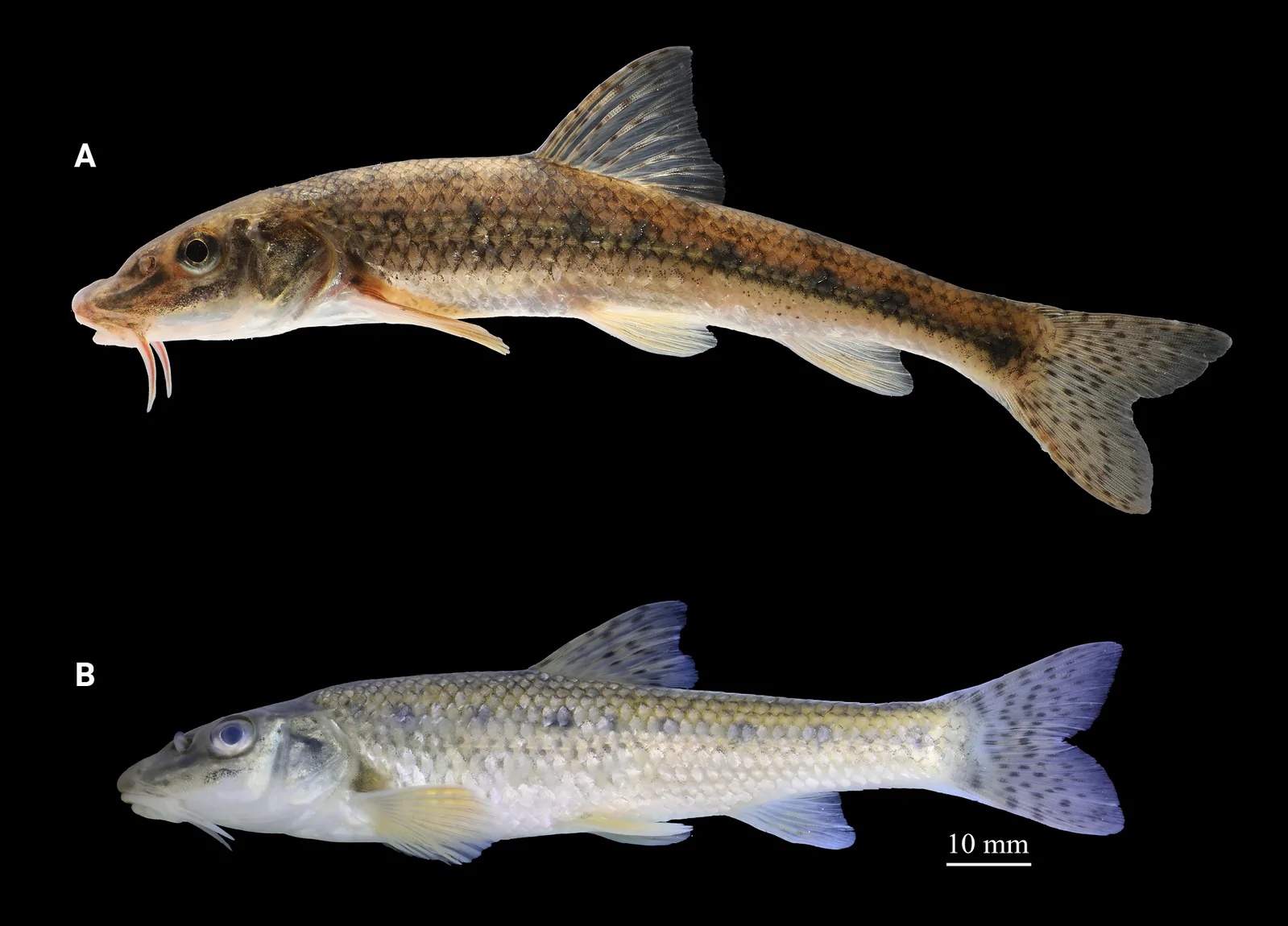
General view of *Gobio macrocephalus*. **A**. Living individual, uncatalogued, Kuaidamao Township, Tonghua County, Tonghua City, Jilin Province, from the Hunjiang River; **B**. ASIZB 249412, 93.3 mm SL.

**Table 7.** Morphometric measurements of *Gobio macrocephalus*.

	***Gobio macrocephalus* (*n* = 13)**		
	**Range**	**Mean**	**SD**
Dorsal-fin rays	7	7	
Anal-fin rays	6	6	
Pectoral-fin rays	14–15	14	
Pelvic-fin rays	7–8	7	
lateral line scales	40–43	42	
Scales above lateral line	7–8.5	7.5	
Scales below lateral line	4–5	4	
Circumpeduncular scales	16–20	16	
SL/mm	69.0–119.7		
**In percent of SL**			
Body depth	16.4–21.7	18.9	1.5
Head length	26.0–28.8	27.3	0.8
Dorsal-fin base length	12.2–14.1	13.1	0.6
Pectoral-fin base length	4.4–6.2	5.3	0.6
Pelvic-fin base length	3.5–4.7	4.0	0.3
Anal-fin base length	7.5–9.1	8.4	0.5
Pre-dorsal length	48.5–50.7	49.4	0.8
Pre-pectoral length	26.0–28.8	27.0	0.9
Pre-pelvic length	48.8–53.4	50.5	1.3
Pre-anal length	69.7–75.5	71.8	1.7
Post-dorsal length	51.4–56.6	54.0	1.6
Caudal peduncle length	17.8–22.3	20.2	1.3
Caudal peduncle depth	7.9–9.7	9.1	0.5
Head length/mm	18.9–32.2		
**In percent of HL**			
Head depth	52.4–57.0	54.2	1.5
Head width	48.6–59.0	52.8	3.1
Eye diameter	22.2–27.7	25.2	1.6
Interorbital width	17.5–21.7	19.9	1.2
Snout length	35.0–41.9	38.7	2.4
Barbel length	20.5–34.8	26.8	3.8

##### Diagnosis.

This species can be distinguished from other *Gobio* species in China by the following combination of characters: head elongated, head length 26.0%–28.8% (mean 27.3%) of standard length (vs mean HL < 26.0% SL in *G. guentheri*, *G. coriparoides*, *G. tumenensis*, *G. huanghensis*, *G. tenuicorpus*, *G. rivuloides*, *G. fushunensis*, *G. cynocephalus*, and *G. acutipinnatus*); circumpeduncular scales 16–20 (vs < 16 in *G. cynocephalus*); anus centrally located between the posterior end of pelvic-fin base and anal-fin origin (vs positioned at posterior one-third in *G. guentheri*, *G. coriparoides*, and *G. soldatovi*).

##### Redescription.

Body elongated, dorsal body profile rising from nostrils to dorsal-fin origin, dropping along dorsal-fin base, then gradually sloping to caudal-fin base. Maximum body depth at dorsal-fin origin, body depth 16.4%–21.7% SL. Head elongated, length larger than body depth, HL 26.0%–28.8% SL; snout elongated, with moderate concavity on top before nostrils. Mouth horseshoe-shaped, inferior, with one pair of maxillary barbels rooted at extremity of upper lip, barbel elongated, reaching vertical through the center of the orbit, barbel length 20.5%–34.8% HL. Upper lip thin, lower lip fused with throat, lips connected at mouth corners. Eyes positioned in dorsal half of head, eye diameter 22.2%–27.7% HL; interorbital region flattened, width smaller than eye diameter, interorbital width 17.5%–21.7% HL. Thoracic region flattened, abdomen rounded. Caudal peduncle robust, laterally compressed. Anus centrally located between the posterior end of pelvic-fin base and anal-fin origin.

Body covered with moderately large cycloid scales. Lateral line complete, almost straight in lateral center. Lateral line scales 40 (1 specimen), 41 (4), 42 (6), 43 (2); scales above lateral line 7 (1), 7.5 (11), 8.5 (1); scales below lateral line 4 (11), 5 (2); circumpeduncular scales 16 (11), 18 (1), 20 (1). Ventral scaleless region only before pectoral-fin base end.

Dorsal fin with three unbranched and seven (13 specimens) branched rays; distal margin concave; origin closer to snout than caudal-fin base. Pectoral fin with one unbranched and 14 (8), 15 (5) branched rays; adpressed pectoral-fin tip not reaching anterior margin of pelvic-fin insertion. Pelvic fin with one unbranched and seven (12), 8(1) branched rays; adpressed pelvic-fin tip reaching posterior to anus. Anal fin with three unbranched and six (13) branched rays; origin closer to pelvic-fin insertion than to caudal-fin base. Caudal fin deeply forked, with nine branched rays in upper lobe and eight branched rays in lower lobe, lobes pointed, upper lobe slightly more pointed than lower lobe.

Gill rakers rudimentary. Pharyngeal teeth “3, 5–5, 3". Swim bladder developed, two chambers; both chambers oblong, posterior larger than anterior; total length approximately equal to coelom.

##### Coloration in life.

Dorsal side of head and body yellowish brown, mid-lateral side greyish yellow, and ventral side grayish white. Lateral side with 7–11 black blotches. Posterior margins of scales on dorsal and lateral sides pigmented black. A yellowish-green stripe with slight fluorescence present above the lateral line. One distinct black stripe between anterior orbit and snout. Fins translucent, dorsal fin, pectoral fin, and caudal fin with black spots; pelvic fin and anal fin with slightly black pigment.

##### Coloration in preservation.

Dorsal side of head and body yellowish brown, mid-lateral side pale yellowish brown, and ventral side grayish white. Lateral side with 7–11 black blotches. Posterior margins of scales on dorsal and lateral sides pigmented black. The fluorescent yellowish green stripe faded. One distinct black stripe between anterior orbit and snout. Fins pale, dorsal fin, pectoral fin, and caudal fin with black spots; pelvic fin and anal fin with slightly black pigment.

##### Sexual dimorphism.

No significant sexual dimorphism observed.

##### Distribution.

**Figure 17.**
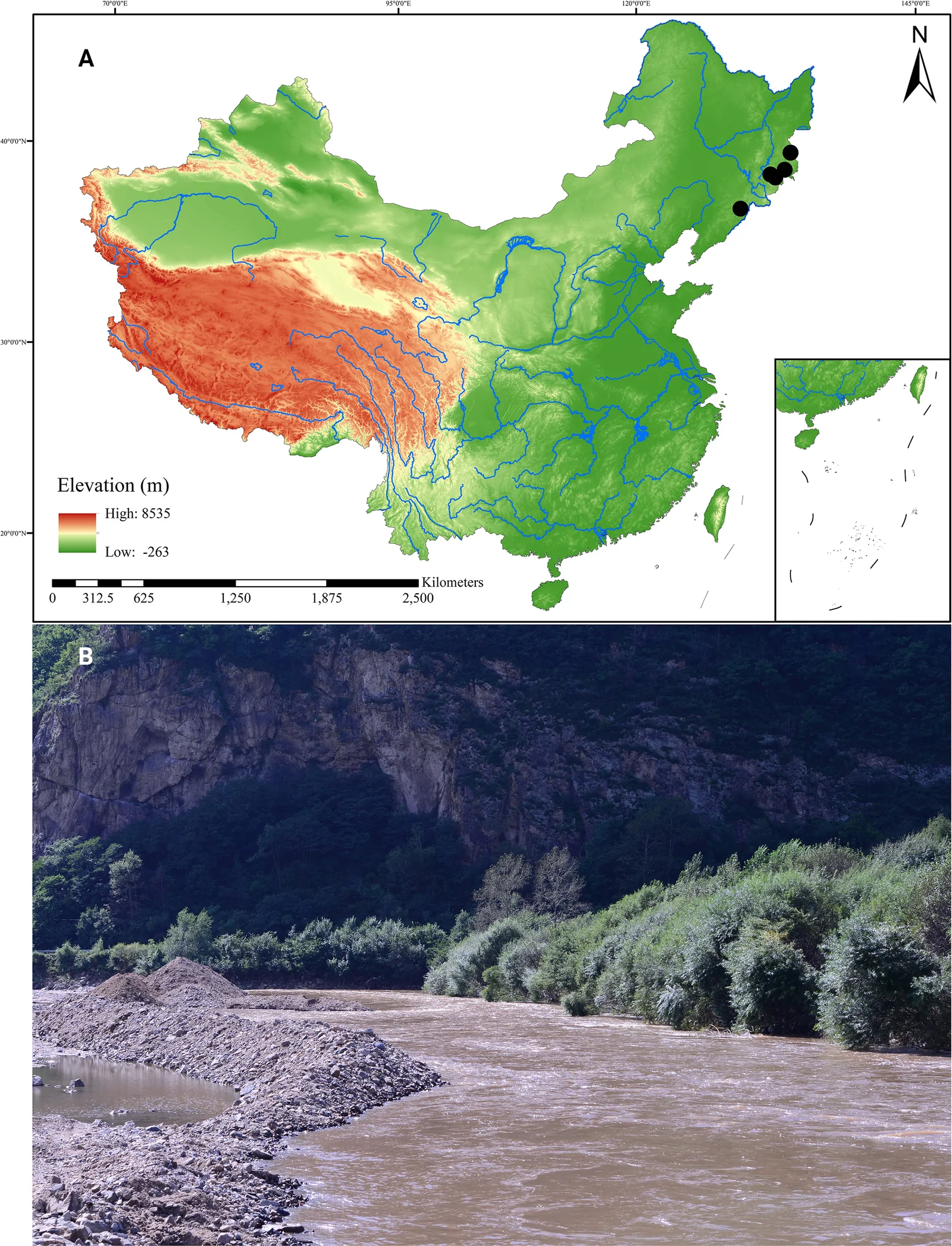
Distribution and habitat of *Gobio macrocephalus*. **A**. Geographic distribution of *G. macrocephalus*; **B**. The Gayahe River, a tributary of the Tumen River drainage, habitat of *G. macrocephalus*, photographed in Wangqing County, Yanbian Chaoxianzu Autonomous Prefecture, Jilin Province.

*Gobio macrocephalus* is distributed in the Yalu, Tumen, Suifenhe, and Amur river drainages. It is rare in the Yalu and Amur river drainages, where it is found only in the Hunjiang River (a tributary of the Yalu River drainage) and the Erdaobaihe River (a tributary of the Amur River drainage). In contrast, it is widespread in both the main streams and tributaries of the Tumen and Suifenhe river drainages. (Fig. 17A).

##### Habitat.

*Gobio macrocephalus* usually appears in fast-flowing water with sandy bottoms mixed with gravel and pebbles (Fig. 17B).

##### Etymology.

The name macrocephalus is derived from Latin elements *macro*- and -*cephalus*. The prefix *macro*- means large, and the suffix -*cephalus* means head. This name refers to the species having a relatively elongated head. The Chinese common name for this species is “大头鮈” (Pinyin: dà tóu jū).

#### Gobio tenuicorpus

Taxon classificationAnimaliaCypriniformesCyprinidae

Mori, 1934

D92C1279-D064-5874-BA41-41414F28B87C

Fig. 18; Table 8

Gobio gobio tenuicorpus Mori, 1934: 13.
Gobio tenuicorpus : Luo et al. 1977: 501; Yue 1998: 300; Xie et al. 2007: 181; Zhang et al. 2016: 72.
Romanogobio tenuicorpus : Bogutskaya and Naseka 1997: 39; Antonov et al. 2019: 110.

##### Material examined.

ASIZB 241499–241500, 242000–242002, • 5 specimens, 89.8–98.5 mm SL; Xunke County, Heihe City, Heilongjiang Province, from the Amur River (49.564°N, 128.368°E, ca 80 m a.s.l.); 12 May 2022. ASIZB 239619, 239621, • 2 specimens, 72.0–97.4 mm SL; Luanping County, Chengde City, Hebei Province, from the Luanhe River (40.988°N, 117.499°E, ca 420 m a.s.l.); 6 October 2023. ASIZB 248252–248266, • 15 specimens, 104.6–124.0 mm SL; Shuangluan Distinct, Chengde City, Hebei Province, from the Luanhe River (40.947°N, 117.791°E, ca 360 m a.s.l.); 20 November 2023. ASIZB 248288–248289, • 2 specimens, 70.5–89.9 mm SL; Sujiatun Distinct, Shenyang City, Liaoning Province, from the Beishahe River, a tributary of the Liaohe River (41.593°N, 123.487°E, ca 50 m a.s.l.); 2 June 2025.

**Figure 18.**
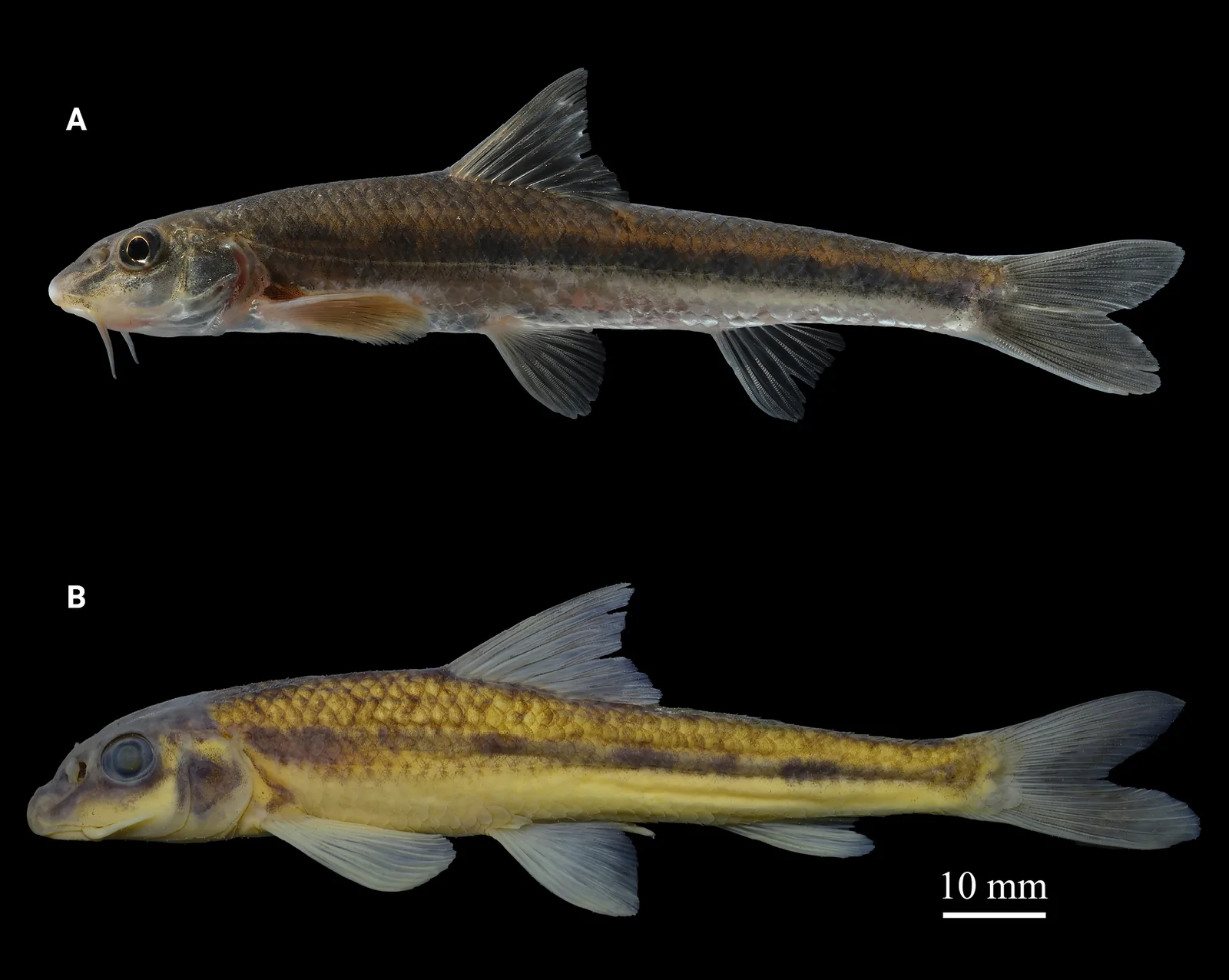
General view of *Gobio tenuicorpus*. **A**. Living individual, uncatalogued, Sujiatun Distinct, Shenyang City, Liaoning Province, from the Hunjiang River; **B**. ASIZB 242000, 92.5 mm SL.

**Table 8.** Morphometric measurements of *Gobio tenuicorpus*.

	***Gobio tenuicorpus* (*n* = 24)**		
	**Range**	**Mean**	**SD**
Dorsal-fin rays	7	7	
Anal-fin rays	6	6	
Pectoral-fin rays	13–14	13	
Pelvic-fin rays	7	7	
lateral line scales	43–46	45	
Scales above lateral line	5.5–6.5	6.5	
Scales below lateral line	3–4	3	
Circumpeduncular scales	12–16	14	
SL/mm	70.5–124.0		
**In percent of SL**			
Body depth	14.8–21.8	16.6	1.5
Head length	21.4–24.2	22.6	0.7
Dorsal-fin base length	12.2–15.0	14.2	0.6
Pectoral-fin base length	4.7–6.1	5.4	0.4
Pelvic-fin base length	3.7–5.4	4.6	0.4
Anal-fin base length	8.0–9.9	8.9	0.5
Pre-dorsal length	41.1–44.8	43.1	0.9
Pre-pectoral length	21.2–26.1	22.8	0.9
Pre-pelvic length	44.9–48.5	46.9	1.0
Pre-anal length	69.3–73.4	71.6	1.0
Post-dorsal length	57.1–61.1	59.3	1.1
Caudal peduncle length	19.0–22.2	20.1	0.9
Caudal peduncle depth	6.9–9.3	7.6	0.6
Head length/mm	15.6–27.3		
**In percent of HL**			
Head depth	51.5–59.4	54.8	2.3
Head width	49.4–65.8	61.1	3.7
Eye diameter	26.8–32.3	29.2	1.5
Interorbital width	19.0–24.5	21.6	1.5
Snout length	34.8–41.9	38.9	1.8
Barbel length	29.0–42.1	34.5	3.3

##### Diagnosis.

This species can be distinguished from other *Gobio* species in China by the following combination of characters: lateral-line scales 43–46 (vs < 43 in *G. coriparoides*, *G. soldatovi*, and *G. fushunensis*); circumpeduncular scales 12–16 (vs > 16 in *G. guentheri*); ventral scaleless region extends to middle of pectoral-fin base and to pelvic-fin base (vs not extending beyond pectoral-fin base in *G. soldatovi*, *G. tumenensis*, *G. huanghensis*, *G. macrocephalus*, *G. fushunensis*, *G. cynocephalus*, and *G. acutipinnatus*); anus positioned at anterior one-third of distance from pelvic-fin base end to anal-fin origin (vs centrally located or in posterior one-third in other Chinese *Gobio* species).

##### Redescription.

Body elongated, dorsal body profile rising from nostrils to dorsal-fin origin, dropping along dorsal-fin base, then gradually sloping to caudal-fin base. Maximum body depth at dorsal-fin origin, body depth 14.8%–21.8% SL. Head elongated, length larger than body depth, HL 21.4%–24.2% SL; snout elongated, with moderate concavity on top before nostrils. Mouth horseshoe-shaped, inferior, with one pair of maxillary barbels rooted at extremity of upper lip, barbel elongated, reaching vertical through the posterior of the orbit, barbel length 29.0%–42.1% HL. Upper lip thin, lower lip fused with throat, lips connected at mouth corners. Eyes positioned in dorsal half of head, eye diameter 26.8%–32.3% HL; interorbital region flattened, width smaller than eye diameter, interorbital width 19.0%–24.5% HL. Thoracic region flattened, abdomen rounded. Caudal peduncle elongated, laterally compressed. Anus positioned at anterior one-third of distance from pelvic-fin base end to anal-fin origin.

Body covered with moderately large cycloid scales. Lateral line complete, almost straight in lateral center. Lateral line scales 43 (3 specimens), 44 (11), 45 (7), 46 (3); scales above lateral line 5.5 (4), 6 (3), 6.5 (17); scales below lateral line 3 (21), 4 (3); circumpeduncular scales 12 (3), 14 (20), 16 (1). Ventral scaleless region extends to middle of pectoral-fin base and to pelvic-fin base.

Dorsal fin with three unbranched and seven (24 specimens) branched rays; distal margin concave; origin closer to snout than caudal-fin base. Pectoral fin with one unbranched and 13 (15), 14 (9) branched rays; adpressed pectoral-fin tip not reaching anterior margin of pelvic-fin insertion. Pelvic fin with one unbranched and seven (24) branched rays; adpressed pelvic-fin tip reaching posterior to anus. Anal fin with three unbranched and six (24) branched rays; origin closer to pelvic-fin insertion than to caudal-fin base. Caudal fin deeply forked, with nine branched rays in upper lobe and eight branched rays in lower lobe, lobes pointed, upper lobe slightly more pointed than lower lobe.

Gill rakers rudimentary. Pharyngeal teeth “3, 5–5, 3". Swim bladder developed, two chambers; both chambers oblong, posterior larger than anterior; total length approximately equal to coelom.

##### Coloration in life.

Dorsal side of head and body yellowish brown, mid-lateral side pale yellowish brown, and ventral side grayish white. Lateral side with 8–10 black blotches. Posterior margins of scales on dorsal and lateral sides pigmented black. A yellowish-green stripe with slight fluorescence present above the lateral line. One distinct black stripe between anterior orbit and snout. Fins translucent, with scattered black spots.

##### Coloration in preservation.

Dorsal side of head and body greyish yellow, mid-lateral side pale greyish yellow, and ventral side grayish white. Lateral side with 8–10 black blotches. Posterior margins of scales on dorsal and lateral sides pigmented black. The fluorescent yellowish green stripe faded. One distinct black stripe between anterior orbit and snout. Fins pale, with scattered black spots.

##### Sexual dimorphism.

No significant sexual dimorphism observed.

##### Distribution.

**Figure 19.**
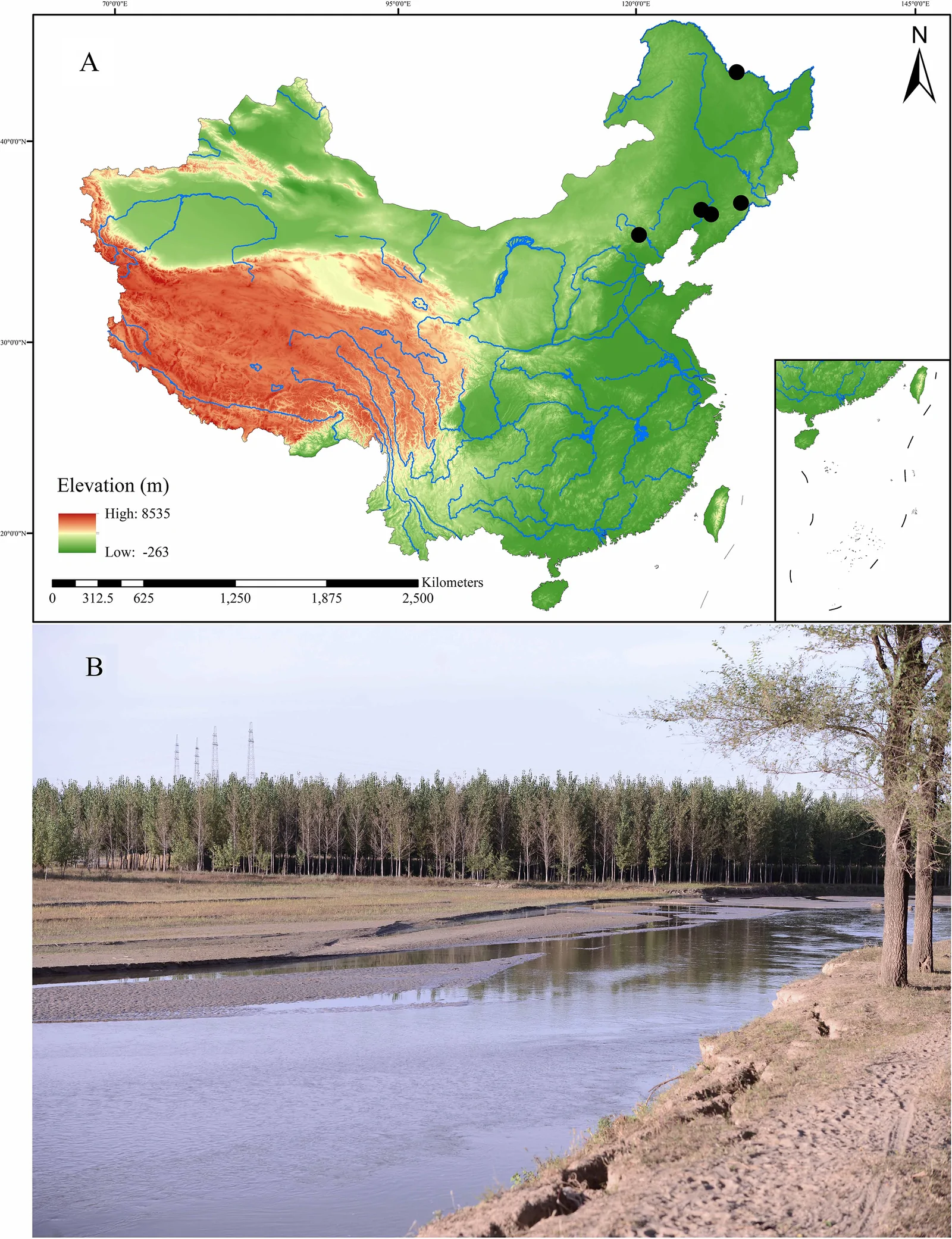
Distribution and habitat of *Gobio tenuicorpus*. **A**. Geographic distribution of *G. tenuicorpus*; **B**. The Liuhe River, a tributary of the Liaohe River drainage, habitat of *G. tenuicorpus*, photographed in Xinmin City, Shenyang City, Liaoning Province.

*Gobio tenuicorpus* is distributed in the main stream or major tributaries of the Luanhe, Liaohe, and Amur river drainages. (Fig. 19A).

##### Habitat.

*Gobio tenuicorpus* usually appears in fast flowing water with sandy bottoms (Fig. 19B).

##### Etymology.

The name *tenuicorpus* is derived from Latin words *tenuis* and *corpus*. The prefix *tenui*- means slender, and the suffix -*corpus* means body. This name refers to the species having a slender body. The Chinese common name for this species is “细体鮈” (Pinyin: xì tǐ jū).

##### Remarks.

Bogutskaya and Naseka (1997) placed *Gobio tenuicorpus* in the genus *Romanogobio* and referred to it as *Romanogobio tenuicorpus*. The phylogenetic tree of GobionidaeYang et al. (2006) constructed based on the *cytb* gene showed that *R. tenuicorpus* was nested within the genus *Gobio*. Thus, they reassigned *R. tenuicorpus* to *G. tenuicorpus* (Yang et al. 2006). Subsequently, Tang et al. (2011) supported this taxonomic reassignment. Our study (Fig. 1) confirms that *G. tenuicorpus* is a monophyletic group, nests within the genus *Gobio*, and forms a sister group with *G. rivuloides*.

**Figure 20.**
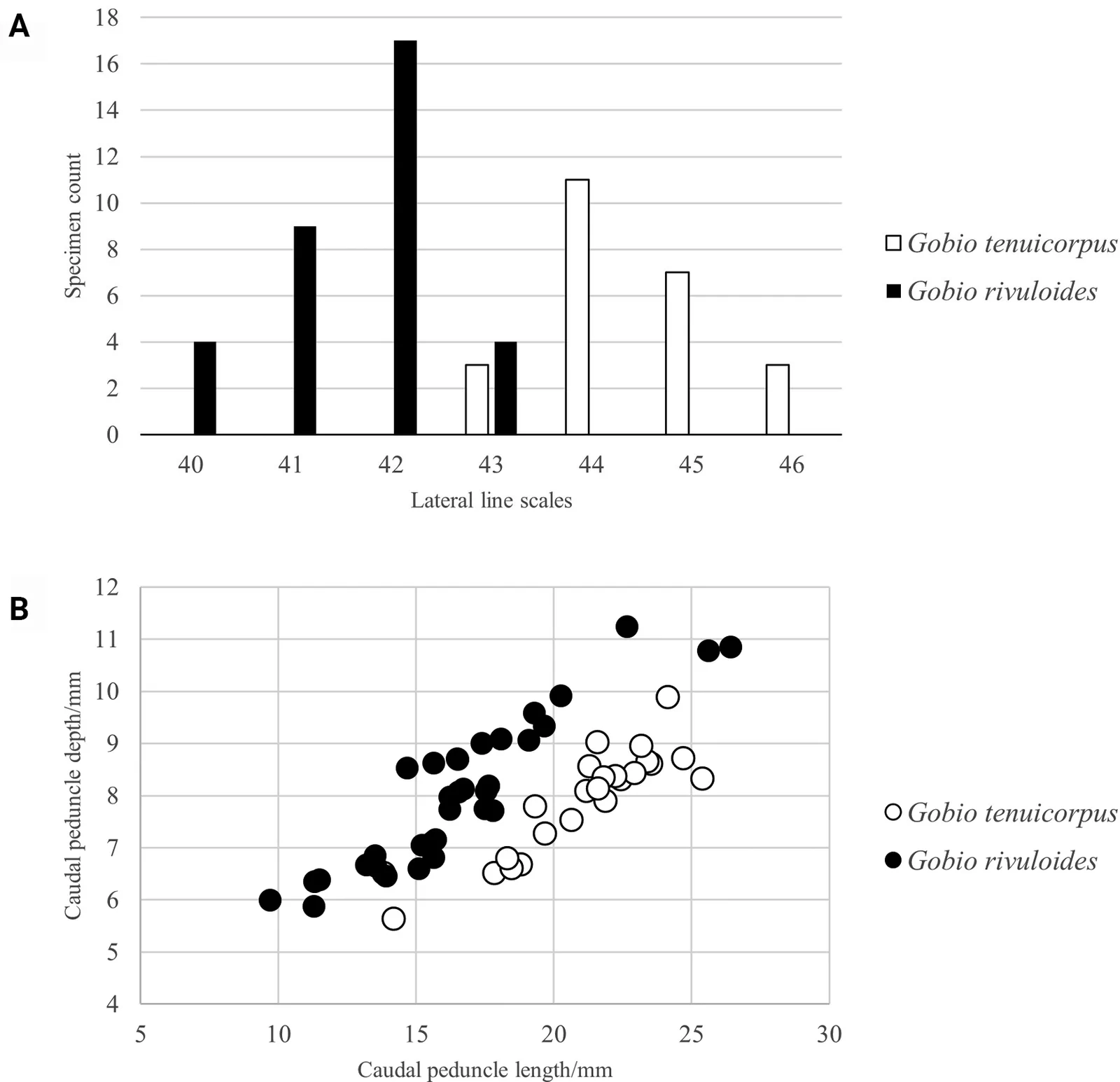
Morphological comparisons between *Gobio tenuicorpus* and *G. rivuloides*. **A**. Frequency distribution of lateral line scale counts; **B**. Caudal peduncle length (mm) plotted against caudal peduncle depth (mm).

**Figure 21.**
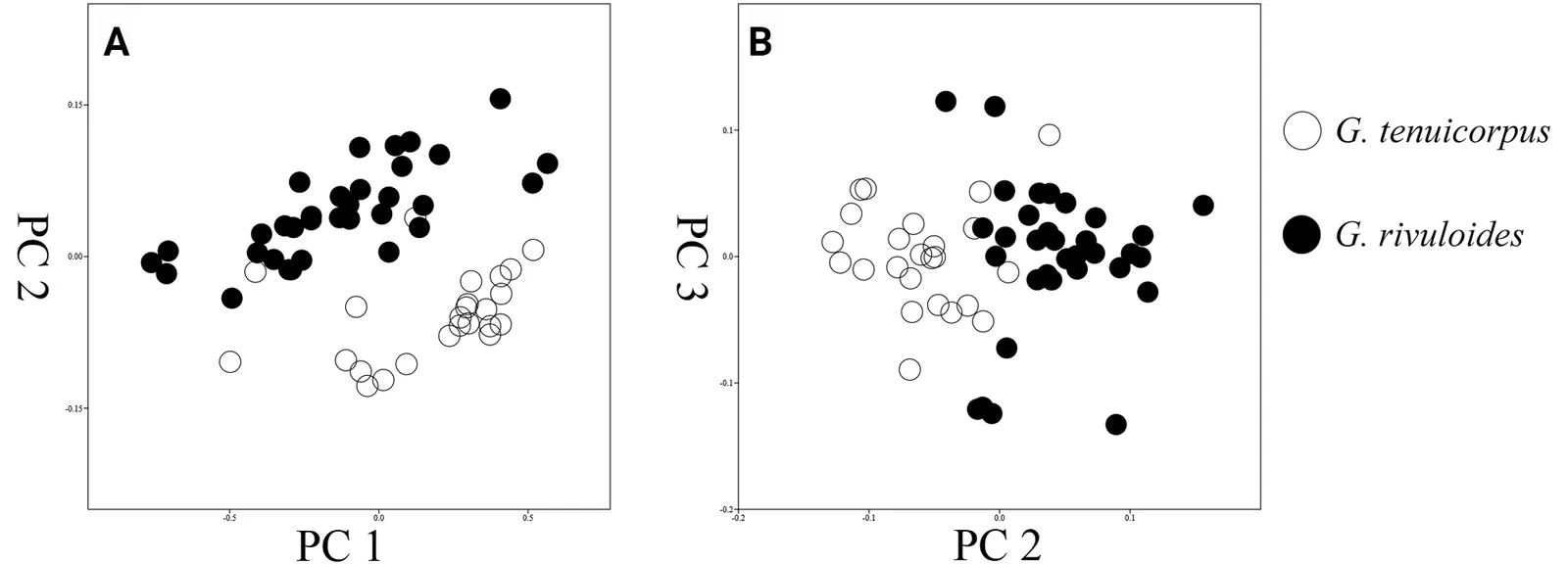
**A**. Scatterplot of PC1 against PC2; **B**. PC2 against PC3 extracted from 19 morphometric data of *Gobio tenuicorpus* and *G. rivuloides*.

The genetic distance between *Gobio tenuicorpus* and *G. rivuloides* is 0.0115, which represents the lowest interspecific distance within the genus *Gobio* (Table 5). Some studies have considered *G. tenuicorpus* a distinct lineage from *G. rivuloides* (Ni et al. 2023). We measured the morphological characters and compared *G. tenuicorpus* with *G. rivuloides*. We found that *G. tenuicorpus* differs from *G. rivuloides* in having more lateral line scales (Fig. 20A) and a more slender caudal peduncle (Fig. 20B). The Mann-Whitney U tests with Bonferroni correction confirmed these differences, with significant differences detected in lateral-line scale counts (adjusted *p* = 0.000016) and caudal peduncle depth (adjusted *p* = 0.000241). We also performed a PCA to further compare the morphological difference between these two species. The PCA results are presented in Fig. 21 and the loadings are given in Table 9. The first three components contributed 94.3% of the variance. Both PC 1 versus PC 2 plot and PC 2 versus PC 3 plot can separate these two species. Those results showed a clear morphological differentiation trend between *G. tenuicorpus* and *G. rivuloides*. Given the reason that *G. tenuicorpus* forms a monophyletic group itself, the stable and distinguishable morphological differences from *G. rivuloides*, and the allopatrical distribution from latter, this study suggests *G. tenuicorpus* as a valid species.

**Table 9.** Loadings on the first three principal components extracted from morphometric data of *Gobio tenuicorpus* and *G. rivuloides*. Bold values indicate relatively high loadings.

	**PC 1**	**PC 2**	**PC 3**
Standard length	0.246	-0.138	-0.007
Body depth	0.184	**0.522**	0.297
Head length	0.207	-0.062	-0.020
Head depth	0.188	0.133	0.167
Head width	0.239	0.048	**0.428**
Dorsal-fin base length	0.254	-0.098	-0.007
Pectoral-fin base length	0.240	0.098	**-0.434**
Pelvic-fin base length	0.263	0.203	-0.098
Anal-fin base length	0.255	0.052	-0.030
Caudal peduncle length	0.280	-0.113	0.193
Caudal peduncle width	0.161	**0.557**	0.063
Eye diameter	0.211	-0.332	0.310
Interorbital width	0.184	0.220	**-0.540**
Snout length	0.249	-0.289	-0.246
Pre-dorsal length	0.226	-0.081	0.064
Pre-pectoral length	0.199	-0.001	-0.069
Pre-pelvic length	0.227	-0.054	-0.027
Pre-anal length	0.242	-0.140	-0.047
Post-dorsal length	0.262	-0.166	0.018

#### Gobio rivuloides

Taxon classificationAnimaliaCypriniformesCyprinidae

Nichols, 1925

BCA2BAA8-36BF-5628-9DFD-85966A359733

Fig. 22

Gobio rivuloides Nichols, 1925: 5; Chen et al. 1987: 118; Yue 1998: 297; Li 2015: 179; Zhang et al. 2016: 72.
Saurogobio drakei brevicaudus : Tchang and Shaw 1931: 286.
Gobio shansiensis : Bănărescu and Nalbant 1966: 1.
Gobio (Romanogobio) johntreadwelli : Bănărescu and Nalbant 1973: 145.
Gobio (Romanogobio) amplexilabris : Bănărescu and Nalbant 1973: 147.
Gobio gobio rivuloides : Luo et al. 1977: 500.
Gobio meridionalis : Chen et al. 1987: 119; Yue 1998: 291; Li 2015: 175; Zhang et al. 2016: 72.
Romanogobio johntreadwelli : Naseka 1996: 160.
Romanogobio amplexilabris : Naseka 1996: 160.
Romanogobio rivuloides : Naseka 1996: 160.

##### Diagnosis.

This species can be distinguished from other *Gobio* species in China by the following combination of characters: ventral scaleless region extends to middle of pectoral-fin base and to pelvic-fin base (vs not extending beyond pectoral-fin base in *G. soldatovi*, *G. tumenensis*, *G. huanghensis*, *G. macrocephalus*, *G. fushunensis*, *G. cynocephalus*, and *G. acutipinnatus*); circumpeduncular scales 12–16 (vs > 16 in *G. guentheri*); anus centrally located between pelvic-fin base end and anal-fin origin (vs positioned at posterior one-third in *G. guentheri*, *G. coriparoides*, and *G. soldatovi*, and at anterior one-third in *G. tenuicorpus*). For detailed description see Fang et al. (2025).

**Figure 22.**
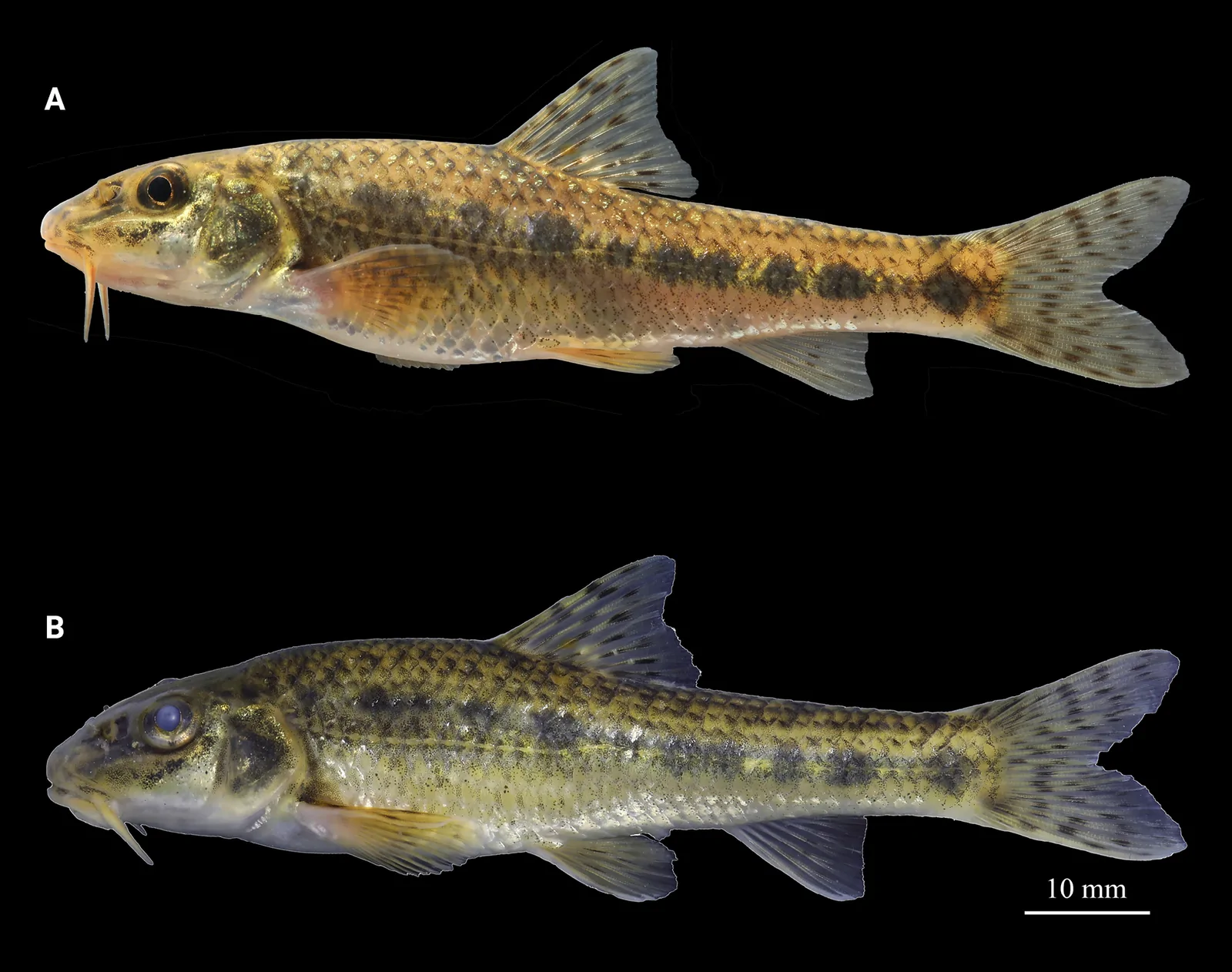
General view of *Gobio rivuloides*. **A**. Living individual, uncatalogued, Maping Township, Luonan County, Shangluo City, Shaanxi Province, from the Luohe River; **B**. ASIZB 242042, 66.9 mm SL (Fang et al. 2025).

##### Distribution.

**Figure 23.**
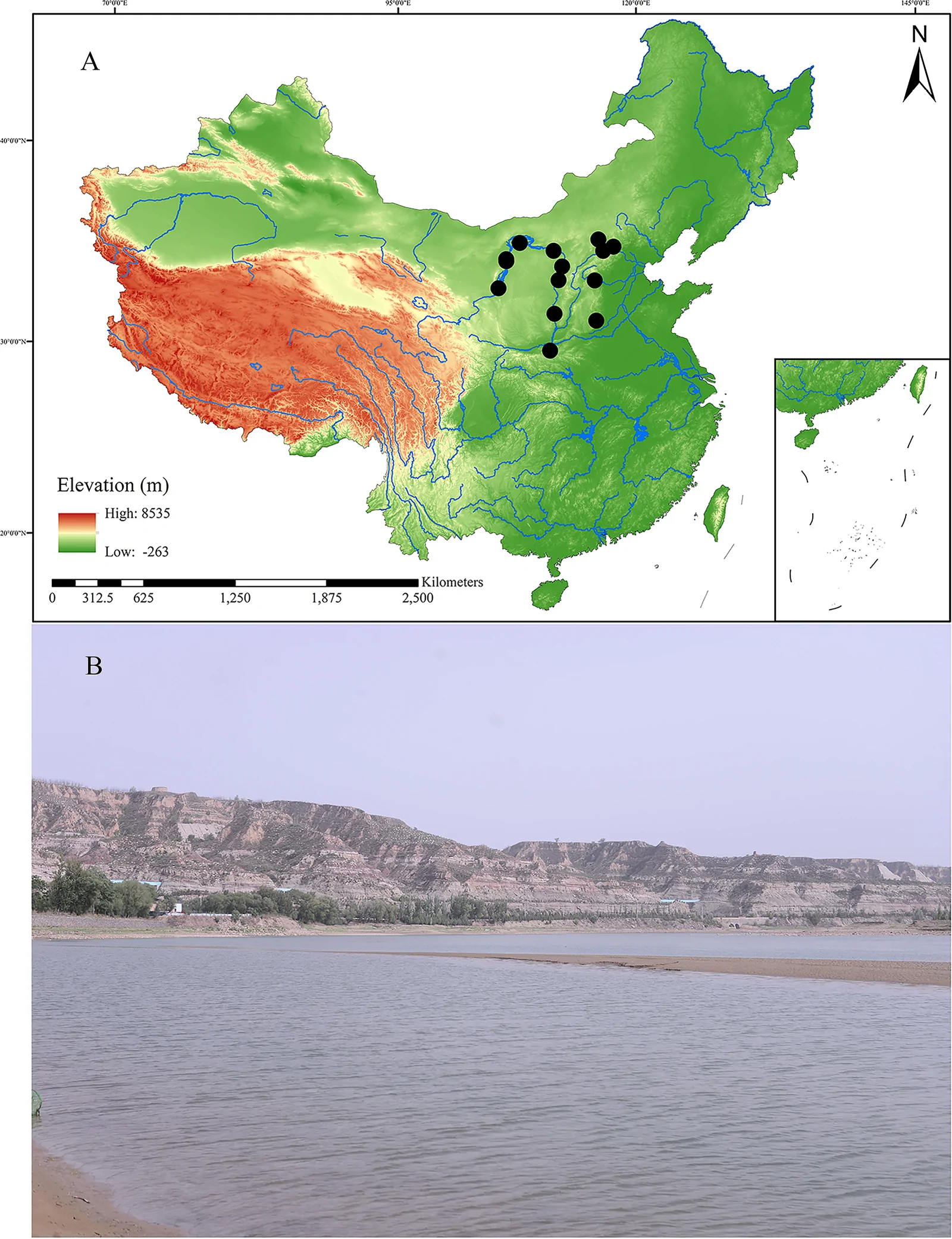
Distribution and habitat of *Gobio rivuloides*. **A**. Geographic distribution of *G. rivuloides*; **B**. The Yellow River, habitat of *G. rivuloides*, photographed in Hequ County, Xinzhou City, Shanxi Province.

*Gobio rivuloides* is distributed in the Yellow and Haihe river drainages (Fig. 23A).

##### Habitat.

*Gobio rivuloides* usually appears in fast-flowing water with sandy bottoms mixed with gravel and pebbles (Fig. 23B).

##### Etymology.

The name *rivuloides* is derived from Latin elements *rivul*- and -*oides*. The prefix *rivul*- means stream, and here refers to the species *Abbottina rivularis*. The suffix -*oides* means similar to. This name indicates that the species is similar to *A. rivularis*. The Chinese common name for this species is “棒花鮈” (Pinyin: bàng huā jū).

##### Remarks.

*Gobio shansiensis* was described by Bănărescu and Nalbant (1966) based on the specimens from Niang-tze-kwan, Shansi Province (=Niangziguan Township, Pingding County, Yangquan City, Shanxi Province), from the Taohe River, a tributary of the Haihe River drainage (not the Hwang-ho drainage, which is synonymous with the Yellow River drainage as referenced in the original description). This species was synonymized with *G. rivuloides* by Luo et al. (1977). We examined specimens of *G. shansiensis*, including the holotype AMNH 20493 and additional voucher specimens: AMNH 10558 (1 specimen), AMNH 29711 (8), AMNH 29714 (1), AMNH 29716 (5), AMNH 29717 (12), and AMNH 29718 (5). These specimens show little morphological differences from *G. rivuloides*. Furthermore, the type locality of *G. shansiensis* is the same as that of *G. rivuloides*. Thus, we agree that *G. shansiensis* should be treated as a junior synonym of *G. rivuloides*.

#### Gobio fushunensis

Taxon classificationAnimaliaCypriniformesCyprinidae

Xie, Li & Xie, 2007

1F0C8C32-5644-5EE4-B4A1-09B47DB94C33

Fig. 24; Table 10

Gobio fushunensis Xie, Li & Xie, 2007: 182.

##### Material examined.

ASIZB 242058–242060, 242063–242077, • 18 specimens, 61.9–80.1 mm SL; Fengcheng City, Dandong City, Liaoning Province, from the Dayanghe River (40.073°N, 123.700°E, ca 10 m a.s.l.); 27 March 2024. ASIZB 242048–242049, • 2 specimens, 78.9–87.0 mm SL; Tonghua County, Tonghua City, Jilin Province, from the Daluoquanhe River (41.625°N, 126.255°E, ca 480 m a.s.l.); 10 October 2024.

**Figure 24.**
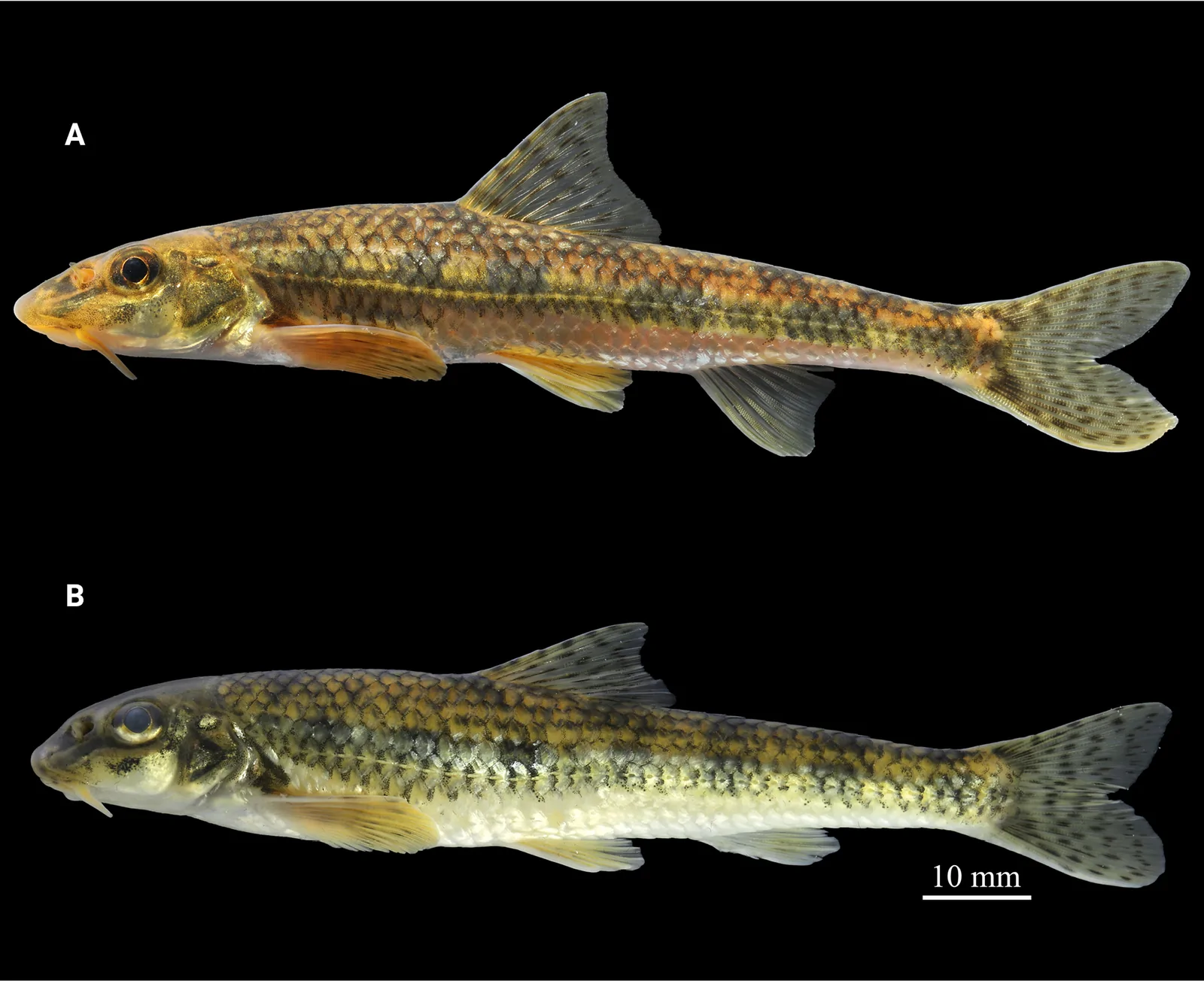
General view of *Gobio fushunensis*. **A**. Living individual, uncatalogued, Lanqi Township, Fengcheng City, Dandong City, Liaoning Province, from the Dayanghe River; **B**. ASIZB 242078, 87.0 mm SL.

**Table 10.** Morphometric measurements of *Gobio fushunensis*.

	***Gobio fushunensis* (*n* = 20)**		
	**Range**	**Mean**	**SD**
Dorsal-fin rays	6–7	7	
Anal-fin rays	6	6	
Pectoral-fin rays	12–14	13	
Pelvic-fin rays	7	7	
lateral line scales	39–41	40	
Scales above lateral line	5–6	5.5	
Scales below lateral line	3	3	
Circumpeduncular scales	12–16	12	
SL/mm	61.9–87.0		
**In percent of SL**			
Body depth	16.4–20.1	18.0	1.0
Head length	22.3–26.2	24.0	0.9
Dorsal-fin base length	12.4–15.0	13.6	0.8
Pectoral-fin base length	4.7–6.5	5.7	0.5
Pelvic-fin base length	3.7–4.4	4.0	0.2
Anal-fin base length	8.4–10.5	9.4	0.5
Pre-dorsal length	46.0–50.5	47.7	1.4
Pre-pectoral length	22.8–26.5	24.3	0.9
Pre-pelvic length	47.3–52.3	48.7	1.3
Pre-anal length	67.1–73.5	69.4	1.6
Post-dorsal length	53.0–59.3	56.1	1.7
Caudal peduncle length	17.7–24.1	21.8	1.6
Caudal peduncle depth	6.9–8.1	7.5	0.4
Head length/mm	14.8–19.4		
**In percent of HL**			
Head depth	53.0–60.8	57.2	2.3
Head width	60.0–70.7	64.3	2.8
Eye diameter	23.9–32.3	28.6	1.8
Interorbital width	15.7–23.2	20.4	2.0
Snout length	35.5–40.9	37.8	1.4
Barbel length	20.2–36.3	30.0	3.9

##### Diagnosis.

This species can be distinguished from other *Gobio* species in China by the following combination of characters: ventral scaleless region not extending beyond pectoral-fin base (vs extending beyond in *G. guentheri*, *G. coriparoides*, *G. tenuicorpus*, and *G. rivuloides*); lateral-line scales 39–41 (vs > 41 in *G. tumenensis* and *G. tenuicorpus*); circumpeduncular scales 12–16 (vs > 16 in *G. guentheri*); scales above lateral-line 5.5 (vs > 5.5 in *G. guentheri*, *G. coriparoides*, *G. soldatovi*, *G. tumenensis*, *G. huanghensis*, *G. macrocephalus*, *G. cynocephalus*, and *G. acutipinnatus*); anus centrally located between the posterior end of pelvic-fin base and anal-fin origin (vs positioned at posterior one-third in *G. guentheri*, *G. coriparoides*, and *G. soldatovi*).

##### Redescription.

Body elongated, dorsal body profile rising from nostrils to dorsal-fin origin, dropping along dorsal-fin base, then gradually sloping to caudal-fin base. Maximum body depth at dorsal-fin origin, body depth 16.4%–20.1% SL. Head elongated, length larger than body depth, HL 22.3%–26.2 SL; snout elongated, with moderate concavity on top before nostrils. Mouth horseshoe-shaped, inferior, with one pair of maxillary barbels rooted at extremity of upper lip, barbel relatively short, barbel length 20.2%–36.3% HL, reaching vertical through the center of the orbit. Upper lip thin, lower lip fused with throat, lips connected at mouth corners. Eyes positioned in dorsal half of head, eye diameter 23.9%–32.3% HL; interorbital region flattened, width smaller than eye diameter, interorbital width 15.7%–23.2% HL. Thoracic region flattened, abdomen rounded. Caudal peduncle elongated, laterally compressed. Anus centrally located between the posterior end of pelvic-fin base and anal-fin origin.

Body covered with moderately large cycloid scales. Lateral line complete, almost straight in lateral center. Lateral line scales 39 (1 specimen), 40 (13), 41 (6); scales above lateral line 5 (8), 5.5 (9), 6 (3); scales below lateral line 3 (20); circumpeduncular scales 12 (16), 14 (3), 16 (1). Ventral scaleless region only before pectoral-fin base end.

Dorsal fin with three unbranched and six (1 specimen), seven (19) branched rays; distal margin concave; origin closer to snout than caudal-fin base. Pectoral fin with one unbranched and 12 (10), 13 (9), 14 (1) branched rays; adpressed pectoral-fin tip not reaching anterior margin of pelvic-fin insertion. Pelvic fin with one unbranched and seven (20) branched rays; adpressed pelvic-fin tip reaching posterior to anus. Anal fin with three unbranched and six (20) branched rays; origin closer to pelvic-fin insertion than to caudal-fin base. Caudal fin deeply forked, with nine branched rays in upper lobe and eight branched rays in lower lobe, lobes pointed, upper lobe slightly more pointed than lower lobe.

Gill rakers rudimentary. Pharyngeal teeth “3, 5–5, 3". Swim bladder developed, two chambers; both chambers oblong, posterior larger than anterior; total length approximately equal to coelom.

##### Coloration in life.

Dorsal side of head and body yellowish brown, mid-lateral side greyish brown, and ventral side grayish white. Lateral side with 6–9 black blotches. Posterior margins of scales on dorsal and lateral sides pigmented black, forming grid pattern on lateral region of the body. A yellowish-green stripe with slight fluorescence present above the lateral line. One distinct black stripe between anterior orbit and snout. Fins translucent, dorsal fin, pectoral fin, and caudal fin with black spots; pelvic fin and anal fin with slightly black pigment.

##### Coloration in preservation.

Dorsal side of head and body yellowish brown, mid-lateral side pale yellowish brown, and ventral side grayish white. Lateral side with 6–9 black blotches. Posterior margins of scales on dorsal and lateral sides pigmented black, forming grid pattern on lateral region of the body. The fluorescent yellowish green stripe faded. One distinct black stripe between anterior orbit and snout. Fins pale, dorsal fin, pectoral fin, and caudal fin with black spots; pelvic fin and anal fin with slightly black pigment.

##### Sexual dimorphism.

No significant sexual dimorphism observed.

##### Distribution.

**Figure 25.**
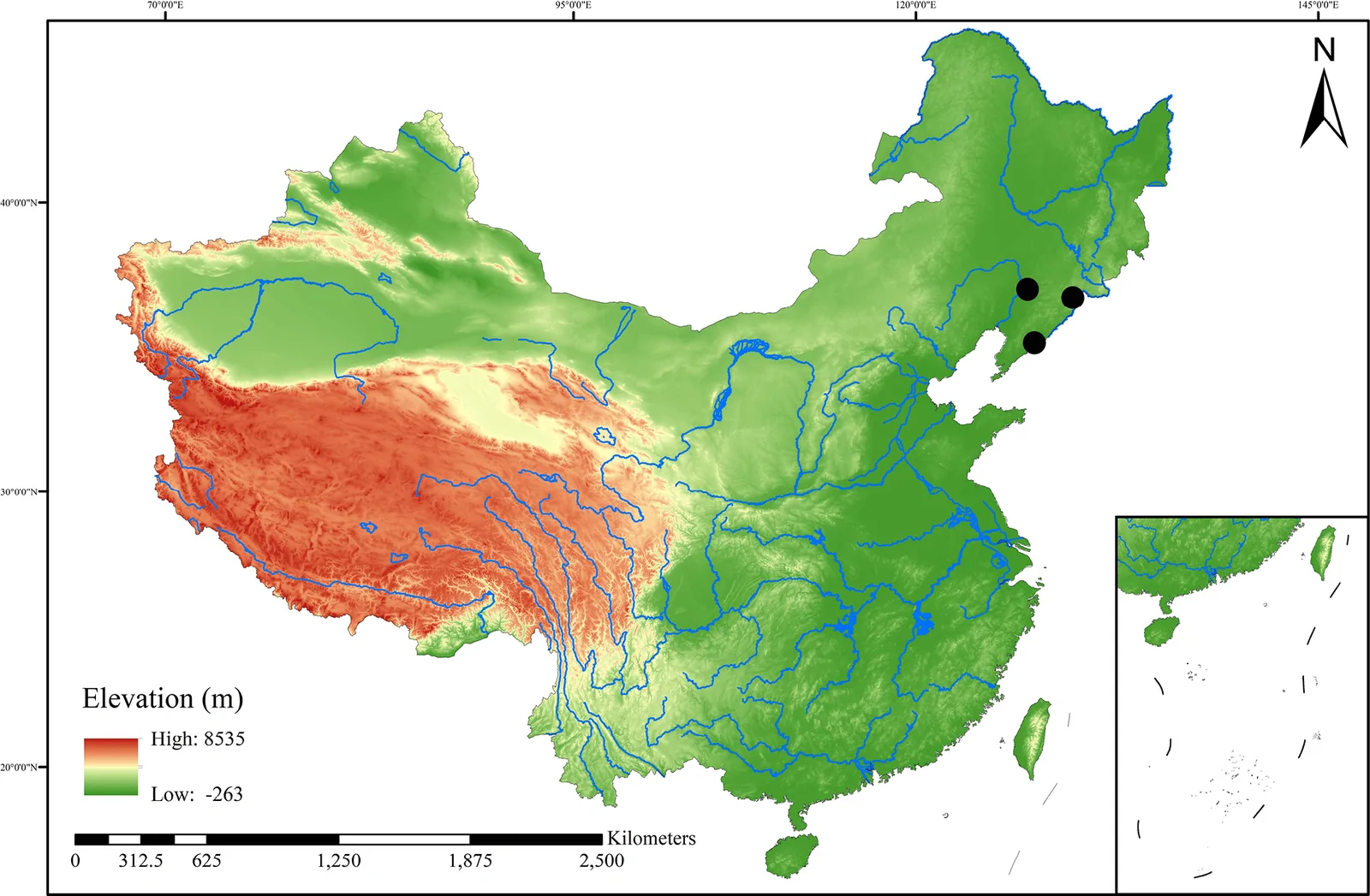
Geographic distribution of *Gobio fushunensis*.

*Gobio fushunensis* is distributed in the Liaohe, Dayanghe, and Yalu river drainages. (Fig. 25).

##### Habitat.

*Gobio fushunensis* usually appears in fast-flowing water with sandy bottoms mixed with gravel and pebbles.

##### Etymology.

The name fushunensis refers to Fushun City, Liaoning Province, the type locality of this species. The Chinese common name for this species is “抚顺鮈” (Pinyin: fǔ shùn jū).

#### Gobio cynocephalus

Taxon classificationAnimaliaCypriniformesCyprinidae

Dybowski, 1869

91893537-A212-50AB-8D04-95300F4E6254

Fig. 26; Table 11

Gobio fluviatilis var. cynocephalus Dybowski, 1869: 951.
Gobio gobio cynocephalus : Luo et al. 1977: 497.
Gobio cynocephalus : Yue 1998: 295; Xie et al. 2007: 178; Zhang et al. 2016: 71; Antonov 2019: 90.

##### Material examined.

ASIZB 242003–242005, 242090, • 4 specimens, 92.4–101.1 mm SL; Acheng Dictinct, Harbin City, Heilongjiang Province, from the Toudaohe River (45.420°N, 127.364°E, ca 260 m a.s.l.); 28 September 2023. ASIZB 242080–242089, • 10 specimens, 62.2–86.3 mm SL; Xunke County, Heihe City, Heilongjiang Province, from the Wudihe River (49.338°N, 128.737°E, ca 120 m a.s.l.). ASIZB 242091–242097, • 7 specimens, 72.8–93.7 mm SL; Xunke County, Heihe City, Heilongjiang Province, from the Xunhe River (49.378°N, 128.626°E, ca 130 m a.s.l.), 5 April 2023. ASIZB 248267–248268, • 2 specimens, 89.4–92.8 mm SL; Xunke County, Heihe City, Heilongjiang Province, from the Amur River (49.564°N, 128.368°E, ca 80 m a.s.l.), 12 May 2022.

**Figure 26.**
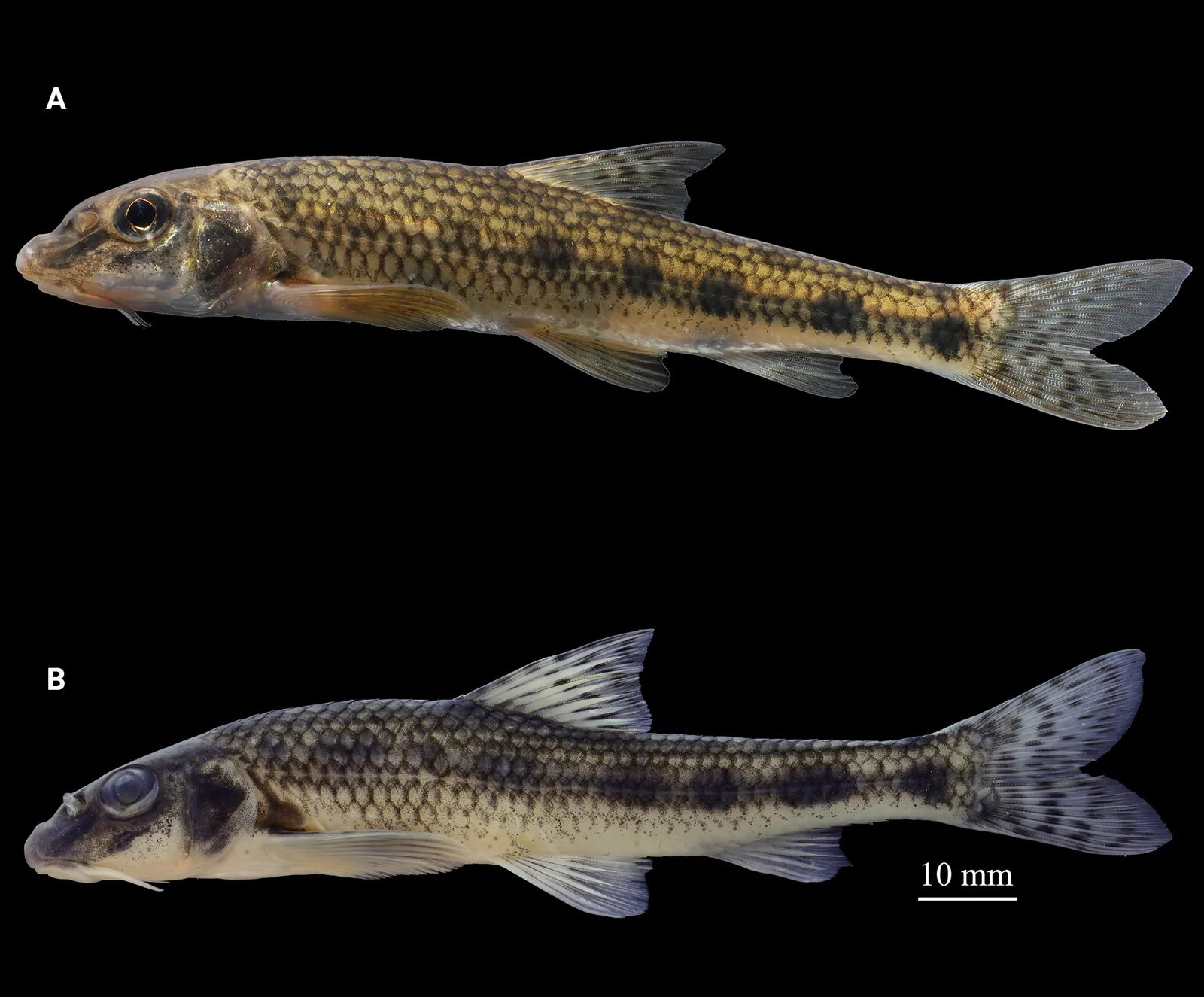
General view of *Gobio cynocephalus*. **A**. Living individual, uncatalogued, Songfengshan Township, Acheng Distinct, Harbin City, Heilongjiang Province, from the Toudaohe River; **B**. ASIZB 242090, 92.4 mm SL.

**Table 11.** Morphometric measurements of *Gobio cynocephalus*.

	***Gobio cynocephalus* (*n* = 23)**		
	**Range**	**Mean**	**SD**
Dorsal-fin rays	7	7	
Anal-fin rays	6	6	
Pectoral-fin rays	13–15	14	
Pelvic-fin rays	7	7	
lateral line scales	41–45	43	
Scales above lateral line	6.5	6.5	
Scales below lateral line	3–4	3	
Circumpeduncular scales	12–14	14	
SL/mm	62.2–101.1		
**In percent of SL**			
Body depth	15.7–20.4	18.1	1.4
Head length	23.1–26.3	25.0	0.8
Dorsal-fin base length	12.0–14.5	13.3	0.7
Pectoral-fin base length	4.4–6.4	5.3	0.5
Pelvic-fin base length	3.8–5.1	4.6	0.3
Anal-fin base length	7.4–8.9	8.3	0.4
Pre-dorsal length	43.7–49.8	47.3	1.6
Pre-pectoral length	23.6–26.1	24.7	0.7
Pre-pelvic length	47.5–51.3	49.3	1.3
Pre-anal length	70.6–74.9	72.6	1.1
Post-dorsal length	51.5–56.8	54.8	1.4
Caudal peduncle length	18.7–21.4	19.9	0.9
Caudal peduncle depth	6.7–8.8	7.7	0.4
Head length/mm	16.0–25.3		
**In percent of HL**			
Head depth	52.4–60.5	55.8	2.2
Head width	48.9–64.9	55.7	4.9
Eye diameter	28.6–34.0	31.5	1.3
Interorbital width	16.3–22.5	19.6	1.6
Snout length	34.5–38.9	36.9	1.3
Barbel length	31.2–40.5	36.9	2.8

##### Diagnosis.

This species can be distinguished from other *Gobio* species in China by the following combination of characters: barbel relatively elongated, reaching vertical through the posterior of the orbit (vs extending over posterior preopercle margin in *G. guentheri*, *G. tumenensis*, and *G. huanghensis*); ventral scaleless region not extending beyond pectoral-fin base (vs extending beyond in *G. guentheri*, *G. coriparoides*, *G. tenuicorpus*, and *G. rivuloides*); circumpeduncular scales 12–14 (vs > 14 in *G. guentheri*, *G. coriparoides*, *G. tumenensis*, and *G. macrocephalus*); scales above lateral-line 6.5 (vs 5.5 in *G. fushunensis*); anus centrally located between the posterior end of pelvic-fin base and anal-fin origin (vs positioned at posterior one-third in *G. guentheri*, *G. coriparoides*, and *G. soldatovi*, and at anterior one-third in *G. tenuicorpus*); flank with 7–10 distinct large black blotches (vs with 9–12 small black blotches in *G. acutipinnatus*).

##### Redescription.

Body elongated, dorsal body profile rising from nostrils to dorsal-fin origin, dropping along dorsal-fin base, then gradually sloping to caudal-fin base. Maximum body depth at dorsal-fin origin, body depth 15.7%–20.4% SL. Head elongated, length larger than body depth, HL 23.1%–26.3% SL; snout elongated, with moderate concavity on top before nostrils. Mouth horseshoe-shaped, inferior, with one pair of maxillary barbels rooted at extremity of upper lip, barbel elongated, barbel length 31.2%–40.5% HL, reaching vertical through the posterior of the orbit. Upper lip thin, lower lip fused with throat, lips connected at mouth corners. Eyes positioned in dorsal half of head, eye diameter 28.6%–34.0% HL; interorbital region flattened, width smaller than eye diameter, interorbital width 16.3%–22.5% HL. Thoracic region flattened, abdomen rounded. Caudal peduncle elongated, laterally compressed. Anus centrally located between the posterior end of pelvic-fin base and anal-fin origin.

Body covered with moderately large cycloid scales. Lateral line complete, almost straight in lateral center. Lateral line scales 41 (1 specimen), 42 (5), 43 (7), 44 (8), 45 (2); scales above lateral line 6.5 (23); scales below lateral line 3 (18), 4 (5); circumpeduncular scales 12 (3), 14 (20). Ventral scaleless region only before pectoral-fin base end.

Dorsal fin with three unbranched and seven (23) branched rays; distal margin concave; origin closer to snout than caudal-fin base. Pectoral fin with one unbranched and 13 (1), 14 (21), 15 (1) branched rays; adpressed pectoral-fin tip not reaching anterior margin of pelvic-fin insertion. Pelvic fin with one unbranched and seven (23) branched rays; adpressed pelvic-fin tip reaching posterior to anus. Anal fin with three unbranched and six (23) branched rays; origin closer to pelvic-fin insertion than to caudal-fin base. Caudal fin deeply forked, with nine branched rays in upper lobe and eight branched rays in lower lobe, lobes pointed, upper lobe slightly more pointed than lower lobe.

Gill rakers rudimentary. Pharyngeal teeth “3, 5–5, 3". Swim bladder developed, two chambers; both chambers oblong, posterior larger than anterior; total length approximately equal to coelom.

##### Coloration in life.

Dorsal side of head and body yellowish brown, mid-lateral side greyish yellow, and ventral side grayish white. Lateral side with 7–10 black blotches. Posterior margins of scales on dorsal and lateral sides pigmented black, forming grid pattern on lateral region of the body. A yellowish-green stripe with slight fluorescence present above the lateral line. One distinct black stripe between anterior orbit and snout. Fins translucent, with scattered black spots.

##### Coloration in preservation.

Dorsal side of head and body brownish grey, mid-lateral side pale brownish grey, and ventral side grayish white. Lateral side with 7–10 large black blotches. Posterior margins of scales on dorsal and lateral sides pigmented black, forming grid pattern on lateral region of the body. The fluorescent yellowish green stripe faded. One distinct black stripe between anterior orbit and snout. Fins pale, with scattered black spots.

##### Sexual dimorphism.

No significant sexual dimorphism observed.

##### Distribution.

**Figure 27.**
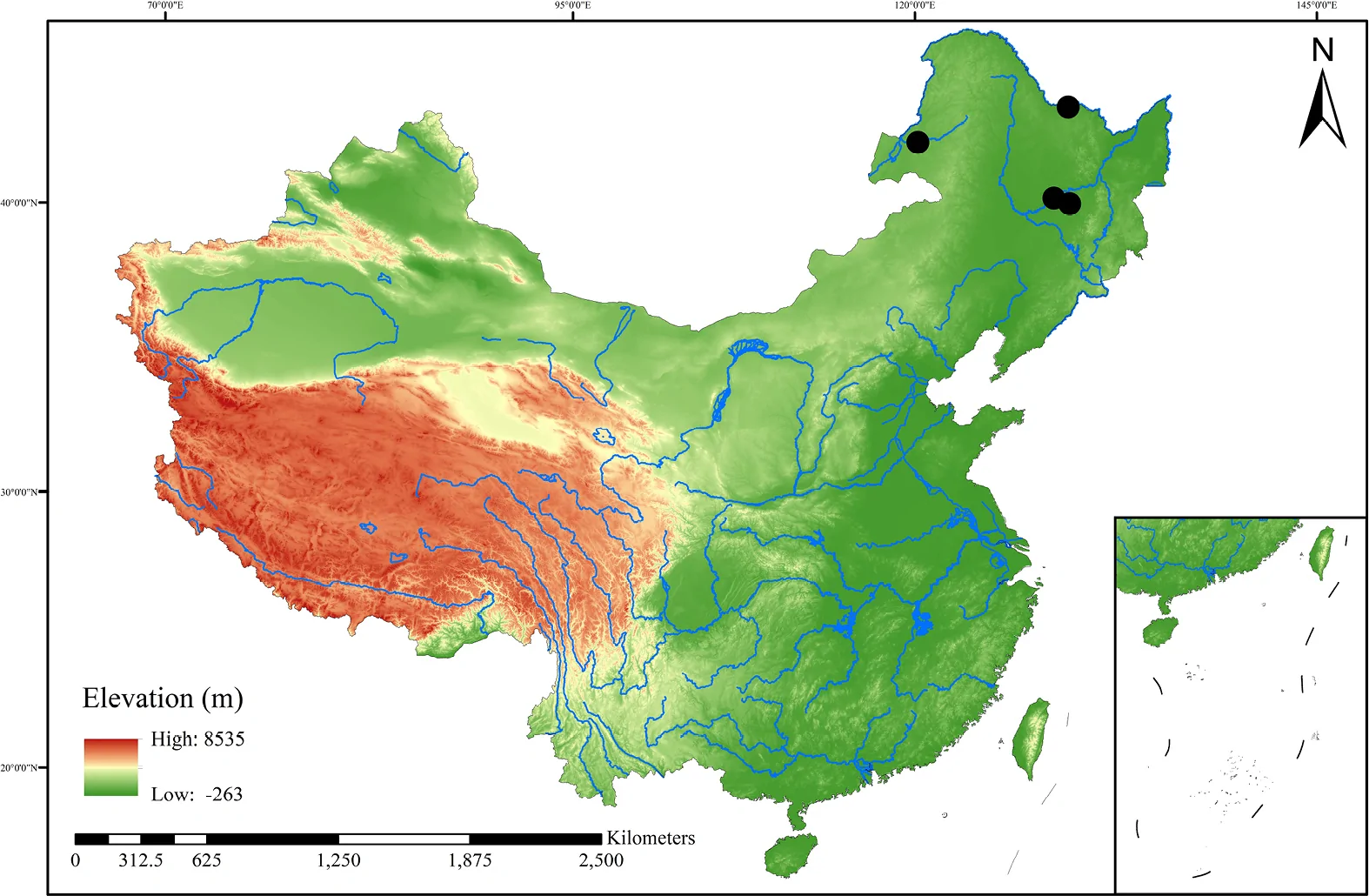
Geographic distribution of *Gobio cynocephalus*.

*Gobio cynocephalus* is distributed in the Amur River drainage. (Fig. 27).

##### Habitat.

*Gobio cynocephalus* usually appears in fast-flowing water with sandy bottoms mixed with gravel and pebbles.

##### Etymology.

The name cynocephalus is derived from Latin elements *cyno*- and -*cephalus*. The prefix *cyno*- means dog-like, and the suffix -*cephalus* means head. This name refers to the concave dorsal snout profile and elongated, pointed snout that give the head a dog-like appearance. The Chinese common name for this species is “犬首鮈” (Pinyin: quǎn shǒu jū).

#### Gobio acutipinnatus

Taxon classificationAnimaliaCypriniformesCyprinidae

Men’shikov, 1939

01619EDA-CB0E-5077-AE22-B153AD3AAE67

Fig. 28; Table 12

Gobio acutipinnatus Men’shikov, 1939: 119; Yue 1998: 294; Zhang et al. 2016: 71.
Gobio gobio cynocephalus : Li et al. 1966: 44.
Gobio gobio acutipinnatus : Luo et al. 1977: 499.
Gobio multipunctatus : Vasil’eva et al. 2023: 849.

##### Material examined.

ASIZB 241460–241462, • 2 specimens, 90.8–94.1 mm SL; Qinghe County, Altay Prefecture, Xinjiang Uygur Autonomous Region, from the Ulungur River (46.053°N, 90.149°E, ca 1080 m a.s.l.); 9 July 2023. ASIZB 241463, • 1 specimen, 100.2 mm SL; Fuyun County, Altay Prefecture, Xinjiang Uygur Autonomous Region, from the Ulungur River (46.482°N, 89.332°E, ca 790 m a.s.l.); 6 July 2023. ASIZB 241464, • 1 specimen, 73.3 mm SL; Fuyun County, Altay Prefecture, Xinjiang Uygur Autonomous Region, from the Ulungur River (46.482°N, 89.334°E, ca 790 m a.s.l.); 25 September 2023. ASIZB 241465, • 1 specimen, 82.8 mm SL; Fuyun County, Altay Prefecture, Xinjiang Uygur Autonomous Region, from the Ulungur River (46.553°N, 88.476°E, ca 660 m a.s.l.); 26 September 2023. ASIZB 241466–241467, • 2 specimens, 102.0–110.9 mm SL; Qinghe County, Altay Prefecture, Xinjiang Uygur Autonomous Region, from the Ulungur River (46.187°N, 90.803°E, ca 1110 m a.s.l.); 9 July 2023. ASIZB 241468–241474, • 7 specimens, 77.6–108.4 mm SL; Qinghe County, Altay Prefecture, Xinjiang Uygur Autonomous Region, from the Ulungur River (46.188°N, 90.802°E, ca 1100 m a.s.l.); 20 September 2023. ASIZB 241475–241476, • 2 specimens, 66.1–78.7 mm SL; Fuyun County, Altay Prefecture, Xinjiang Uygur Autonomous Region, from the Irtysh River (47.215°N, 89.761°E, ca 1140 m a.s.l.); 8 July 2023. ASIZB 241477–241479, • 3 specimens, 78.3–90.9 mm SL; Burqin County, Altay Prefecture, Xinjiang Uygur Autonomous Region, from the Burqinhe River (48.172°N, 87.143°E, ca 710 m a.s.l.); 3 October 2023. ASIZB 241480–241483, • 4 specimens, 66.3–81.1 mm SL; Burqin County, Altay Prefecture, Xinjiang Uygur Autonomous Region, from the Burqinhe River (48.120°N, 87.119°E, ca 660 m a.s.l.); 3 October 2023. ASIZB 241484–241485, • 2 specimens, 108.7–130.8 mm SL; Burqin County, Altay Prefecture, Xinjiang Uygur Autonomous Region, from the Burqinhe River (48.223°N, 87.234°E, ca 760 m a.s.l.); 7 July 2023. ASIZB 241486–241487, • 2 specimens, 62.3–71.9 mm SL; Altay City, Altay Prefecture, Xinjiang Uygur Autonomous Region, from the Irtysh River (47.372°N, 87.816°E, ca 500 m a.s.l.); 28 September 2023.

**Figure 28.**
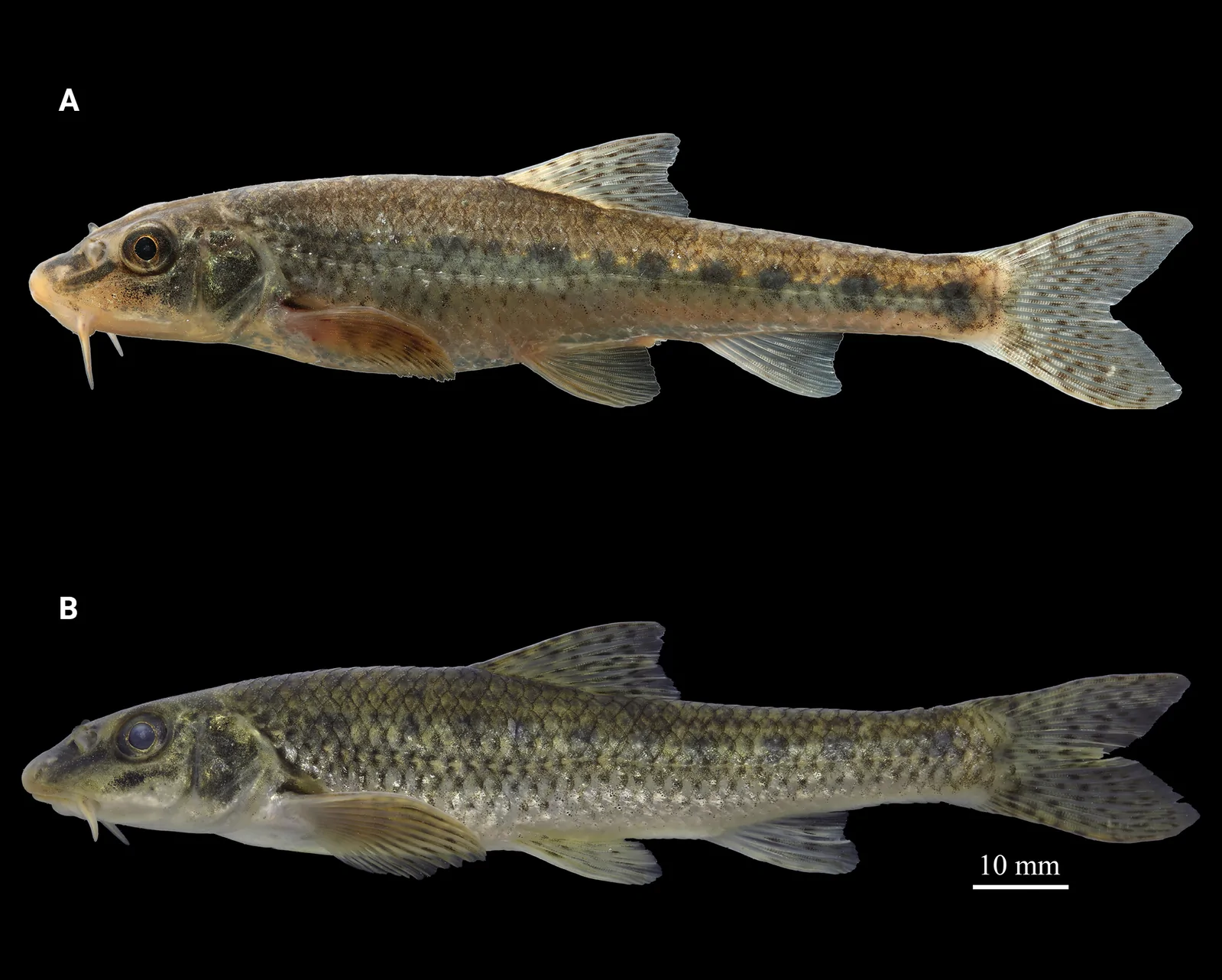
General view of *Gobio acutipinnatus*. **A**. Living individual, uncatalogued, Fuyun County, Altay Prefecture, Xinjiang Uygur Autonomous Region, from the Irtysh River; **B**. Preserved specimen, uncatalogued, same locality as the living individual, 100.3 mm SL.

**Table 12.** Morphometric measurements of *Gobio acutipinnatus*.

	***Gobio acutipinnatus* (*n* = 21)**		
	**Range**	**Mean**	**SD**
Dorsal-fin rays	7–8	7	
Anal-fin rays	6	6	
Pectoral-fin rays	12–15	13	
Pelvic-fin rays	6–7	7	
lateral line scales	40–43	41	
Scales above lateral line	5.5–6.5	6.5	
Scales below lateral line	4	4	
Circumpeduncular scales	12–16	14	
SL/mm	66.3–130.8		
**In percent of SL**			
Body depth	17.6–24.6	19.7	1.7
Head length	24.0–27.5	25.5	0.8
Dorsal-fin base length	11.9–15.1	13.2	0.8
Pectoral-fin base length	4.8–6.3	5.5	0.5
Pelvic-fin base length	3.5–4.8	4.1	0.3
Anal-fin base length	7.6–9.6	8.6	0.6
Pre-dorsal length	46.7–51.8	48.2	1.3
Pre-pectoral length	24.7–28.6	26.6	0.9
Pre-pelvic length	48.7–52.9	50.4	1.2
Pre-anal length	69.8–74.3	71.3	1.4
Post-dorsal length	53.5–57.3	55.1	1.0
Caudal peduncle length	19.7–22.6	21.3	0.8
Caudal peduncle depth	7.9–9.6	8.7	0.4
Head length/mm	16.8–31.4		
**In percent of HL**			
Head depth	51.7–62.8	56.5	2.7
Head width	50.4–66.8	55.6	3.8
Eye diameter	20.9–29.0	24.3	2.4
Interorbital width	21.3–26.1	23.6	1.4
Snout length	38.3–44.3	41.1	2.0
Barbel length	25.0–32.8	28.5	2.6

##### Diagnosis.

This species can be distinguished from other *Gobio* species in China by the following combination of characters: head relatively elongated, head length 24.0%–27.5% (mean 25.5%) of standard length (vs 26.0%–28.8%, mean 27.3% in *G. macrocephalus*); barbel relatively elongated, reaching vertical through the posterior of the orbit (vs reaching beyond posterior preopercle margin in *G. guentheri*, *G. tumenensis*, and *G. huanghensis*); scales above lateral-line 6.5 (vs 5.5 in *G. fushunensis*); ventral scaleless region only before pectoral-fin base (vs extending beyond pectoral-fin base in *G. guentheri*, *G. coriparoides*, *G. tenuicorpus*, and *G. rivuloides*); anus centrally located between the posterior end of pelvic-fin base and anal-fin origin (vs positioned at posterior one-third in *G. guentheri*, *G. coriparoides*, and *G. soldatovi*, and at anterior one-third in *G. tenuicorpus*); flank with 9–12 small black blotches (vs with 9–12 distinct large black blotches in *G. cynocephalus*).

##### Redescription.

Body elongated, dorsal body profile rising from nostrils to dorsal-fin origin, dropping along dorsal-fin base, then gradually sloping to caudal-fin base. Maximum body depth at dorsal-fin origin, body depth 17.6%–24.6% SL. Head elongated, length larger than body depth, HL 24.0%–27.5% SL; snout elongated, with moderate concavity on top before nostrils. Mouth horseshoe-shaped, inferior, with one pair of maxillary barbels rooted at extremity of upper lip, barbel relatively elongated, reaching vertical through the posterior of the orbit, barbel length 25.0%–32.8% HL. Upper lip thin, lower lip fused with throat, lips connected at mouth corners. Eyes positioned in dorsal half of head, eye diameter 20.9%–29.0% HL; interorbital region flattened, width smaller than eye diameter, interorbital width 21.3%–26.1% HL. Thoracic region flattened, abdomen rounded. Caudal peduncle robust, laterally compressed. Anus centrally located between the posterior end of pelvic-fin base and anal-fin origin.

Body covered with moderately large cycloid scales. Lateral line complete, almost straight in lateral center. Lateral line scales 40 (4 specimens), 41 (19), 42 (4), 43 (1); scales above lateral line 5.5 (5), 6 (6), 6.5 (17); scales below lateral line 4 (28); circumpeduncular scales 12 (7), 14 (19), 16 (2). Ventral scaleless region only before pectoral-fin base end.

Dorsal fin with three unbranched and seven (27), eight (1) branched rays; distal margin concave; origin closer to snout than caudal-fin base. Pectoral fin with one unbranched and 12 (6), 13 (20), 14 (1), 15 (1) branched rays; adpressed pectoral-fin tip not reaching anterior margin of pelvic-fin insertion. Pelvic fin with one unbranched and six (2), seven (26) branched rays; adpressed pelvic-fin tip reaching posterior to anus. Anal fin with three unbranched and six (28) branched rays; origin closer to pelvic-fin insertion than to caudal-fin base. Caudal fin deeply forked, with nine branched rays in upper lobe and eight branched rays in lower lobe, lobes pointed, upper lobe slightly more pointed than lower lobe.

Gill rakers rudimentary. Pharyngeal teeth “3, 5–5, 3". Swim bladder developed, two chambers; both chambers oblong, posterior larger than anterior; total length approximately equal to coelom.

##### Coloration in life.

Dorsal side of head and body greyish brown, mid-lateral side greyish yellow, and ventral side grayish white. Lateral side with 9–12 black blotches. Posterior margins of scales on dorsal and lateral sides pigmented black. A yellowish-green stripe with slight fluorescence present above the lateral line. One distinct black stripe between anterior orbit and snout. Fins translucent, with scattered black spots.

##### Coloration in preservation.

Dorsal side of head and body greyish yellow, mid-lateral side pale greyish yellow, and ventral side grayish white. Lateral side with 9–12 small black blotches. Posterior margins of scales on dorsal and lateral sides pigmented black. The fluorescent yellowish green stripe faded. One distinct black stripe between anterior orbit and snout. Fins pale, with scattered black spots.

##### Sexual dimorphism.

No significant sexual dimorphism observed.

##### Distribution.

**Figure 29.**
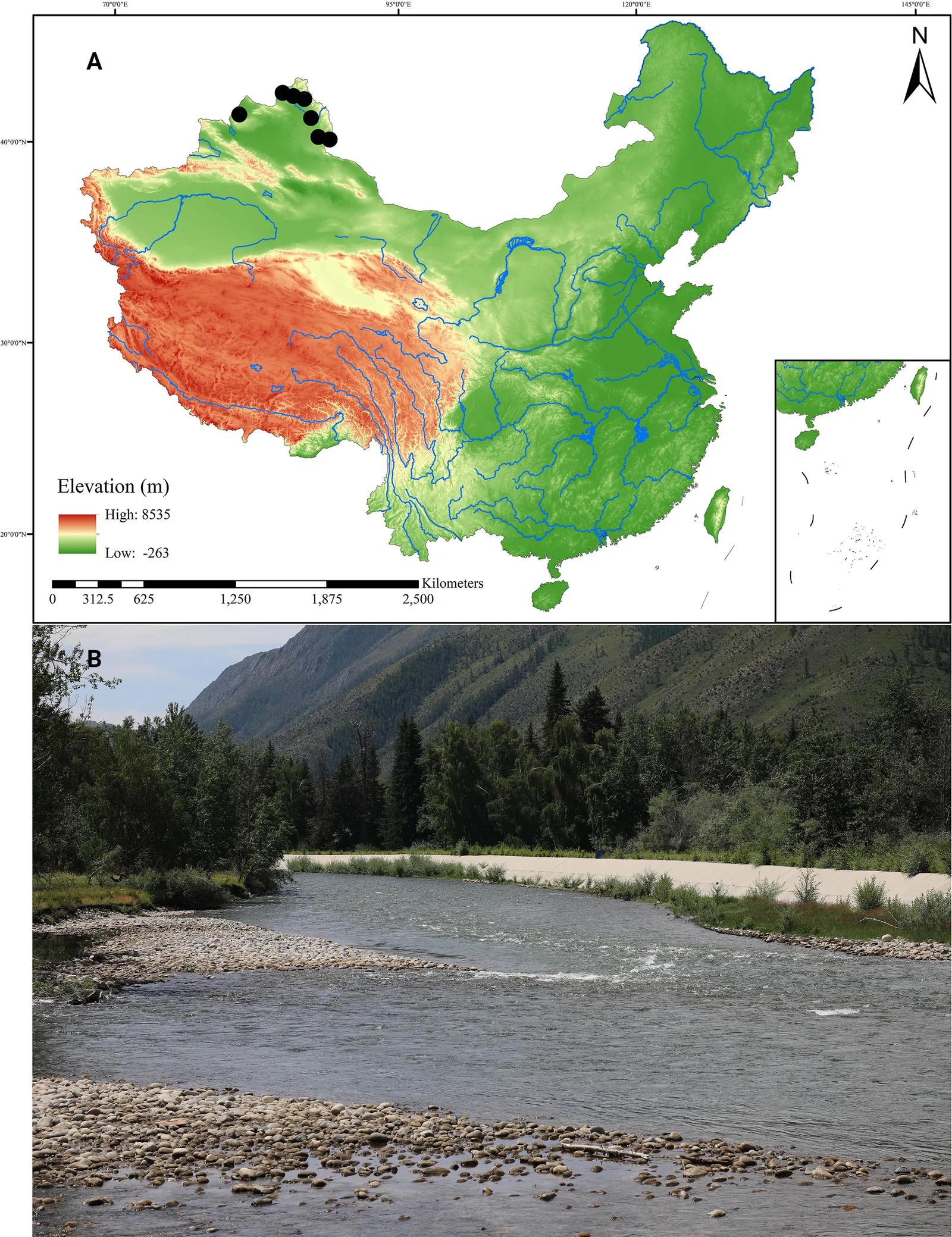
Distribution and habitat of *Gobio acutipinnatus*. **A**. Geographic distribution of *G. acutipinnatus*; **B**. The Irtysh River, habitat of *G. acutipinnatus*, photographed in Tiemaike Township, Fuyun County, Altay Prefecture, Xinjiang Uygur Autonomous Region.

*Gobio acutipinnatus* is distributed in the Irtysh River drainage and the Emin River drainage. (Fig. 29A).

##### Habitat.

*Gobio acutipinnatus* usually appears in fast-flowing water with sandy bottoms mixed with gravel and pebbles (Fig. 29B).

##### Etymology.

The name acutipinnatus is derived from Latin elements *acut*- and -*pinnatus*. The prefix *acut*- means pointed, and the suffix -*pinnatus* means fin. This name refers to the species having pointed fins. The Chinese common name for this species is “尖鳍鮈” (Pinyin: jiān qí jū).

##### Remarks.

Vasil’eva et al. (2023) described *Gobio multipunctatus* based on the specimens collected from the Emin (Emel) River, a tributary of Lake Alakol. This species differs from *G. acutipinnatus* by the following characters: elongated barbel, a greater number of predorsal scales, a lower caudal peduncle, and more spots along the body. We captured two specimens (ASIZB 248277–248278) from the Emin River in Yumin County, Tacheng Prefecture, Xinjiang Uygur Autonomous Region (46.472°N, 82.808°E, ca 390 m a.s.l.). Based on the locality and the original description, we identified these two specimens as *G. multipunctatus*.

We sequenced one of the specimens (ASIZB 248277) and constructed a phylogenetic tree (Fig. 1). The result shows *G. multipunctatus* is nested within *G. acutipinnatus*. Additionally, the genetic distance between *G. multipunctatus* and *G. acutipinnatus* is 0.0049, which is comparable to the intraspecific distance of *G. acutipinnatus* (0.0097) (Table. 5). To maintain the monophyly of *G. acutipinnatus*, we suggest that *G. multipunctatus* a junior synonym of *G. acutipinnatus*. However, as we do not have additional specimens from the Emin River, we are unable to provide morphological comparisons to support this conclusion.

## Discussion

*Mesogobio* was described by Bănărescu and Nalbant (1973). According to the original description, this genus is similar to *Gobio* but is characterized by papillose lips, a dark peritoneum, and a reduced, enclosed swim bladder. Chang (in Zheng et al. 1980) described *M. tumenensis* as the second species of the genus, noting that horny-edged jaws should be added as a diagnostic character to distinguish between *Gobio* and *Mesogobio*. Yang et al. (2006) studied the phylogenetic relationships within the Gobionidae and reported that *M. tumenensis* is nested within the genus *Gobio*. Tang et al. (2011) further corroborated this finding, revising *M. tumenensis* to *G. tumenensis* based on phylogenetic evidence. However, these studies were not able to include the sequence of the type species *M. lachneri*. The validity of the genus *Mesogobio* therefore remains uncertain. Our phylogenetic results (Fig. 1) support the validity of *Mesogobio* and recover it as the sister group of the monophyletic clade comprising *Gobio* (including *G. tumenensis*) and *Romanogobio*.

We dissected numerous specimens, including most of the species of genus *Gobio* from China and *Mesogobio lachneri*, to examine the internal morphological differences between these two genera. The general morphology of the swim bladders are shown in Fig. 13. The swim bladder of *M. lachneri* is reduced, with a total length of approximately half that of the coelom (vs well-developed, with a total length almost equal to that of the coelom in *Gobio*). The swim bladder of *G. tumenensis* is relatively well developed, with its total length approximately two-thirds that of the coelom. This morphological condition is consistent with its placement in *Gobio*. The morphology of the swim bladder appears to be the only reliable character for distinguishing *Mesogobio* from *Gobio*, whereas the other characters mentioned in the original description do not consistently distinguish the two genera. For instance, *Gobio huanghensis* also possesses tiny papillae on the lips, lateral lobes, and chin (Fang et al. 2025), whereas a dark peritoneum is also present in other *Gobio* species (Fig. 13A). Thus, these external and internal characters are not unique to *Mesogobio*.

The genus *Mesogobio* is morphologically very similar to *Gobio*, and the morphology of the swim bladder is currently the primary character used to distinguish the two genera. However, we sought to identify additional external morphological characters that could facilitate the identification of *Mesogobio* and *Gobio* in the field, without the need for dissection or examination of the swim bladder. We therefore examined several morphometric characters, including eye diameter and interorbital width. In *Mesogobio lachneri*, the eye diameter ranges from 28.2%–36.8% HL (mean 32.4%), whereas the interorbital width ranges from 15.3%–17.9% HL (mean 16.8%). However, neither character can reliably distinguish *M. lachneri* from *Gobio* species. For example, the eye diameter of *G. cynocephalus* ranges from 28.6%–34.0% HL (mean 31.5%), which largely overlaps with the range observed in *M. lachneri*. Similarly, the interorbital width of *G. fushunensis* ranges from 15.7%–23.2% HL (mean 20.4%), also overlapping with that of *M. lachneri*. We also observed that mature individuals of *M. lachneri* have distinctly concave distal margins of the dorsal and pectoral fins, whereas this condition is less pronounced in smaller individuals (Fig. 2B). Nevertheless, these characters show ontogenetic variation and overlap with those of *Gobio*, and are therefore not sufficiently consistent to serve as diagnostic characters for distinguishing the two genera. Accordingly, we retain the morphology of the swim bladder as the sole character in the diagnostic key for distinguishing *Mesogobio* from *Gobio*.

Although *Gobio tumenensis* shares several morphological characteristics with *Mesogobio*, including tiny lip papillae and horny-edged jaws, our phylogenetic analyses place it within *Gobio*, a placement further supported by morphological evidence from the swim bladder. These similarities are therefore unlikely to reflect a close phylogenetic relationship and may represent convergent evolution.

Tribe Gobionini is widely distributed across Eurasia. In China, species of this tribe are widely distributed in numerous northern rivers, with the southernmost limit of their distribution occurring in the Luohe River, a tributary of the Yellow River drainage in Shaanxi Province. Only *Gobio chipingi* has been recorded from the Yangtze River (Bănărescu and Nalbant 1964), but this is considered an erroneous locality record (Luo et al. 1977), as no Gobionini species have been reported from the Yangtze River drainage to date. The northernmost and easternmost limits of their distribution in China occur in the Amur River drainage, where *G. cynocephalus*, *G. soldatovi* and *G. tenuicorpus* inhabit. The westernmost limit of their distribution occurs in the Emin River drainage, representing a new distribution record for *G. acutipinnatus*.

## Supplementary Material

XML Treatment for Mesogobio

XML Treatment for Mesogobio lachneri

XML Treatment for Gobio

XML Treatment for Gobio guentheri

XML Treatment for Gobio coriparoides

XML Treatment for Gobio soldatovi

XML Treatment for Gobio tumenensis

XML Treatment for Gobio huanghensis

XML Treatment for Gobio macrocephalus

XML Treatment for Gobio tenuicorpus

XML Treatment for Gobio rivuloides

XML Treatment for Gobio fushunensis

XML Treatment for Gobio cynocephalus

XML Treatment for Gobio acutipinnatus

## Additional information

### Artificial Intelligence (AI) use

The authors accept full responsibility for the content of the manuscript, including the disclosure of any use of AI.

No AI tools were used in the preparation of this manuscript.

### Author ORCIDs

Y. Fang https://orcid.org/0009-0008-5347-9250

Z. Sun https://orcid.org/0000-0003-0565-2801

Y. Zhao https://orcid.org/0000-0002-4615-596X

### Data availability

All of the data that support the findings of this study are available in the main text.

## References

[B1] Antonov A, Barabanschikov E, Zolotukhin S, Mikheev I, Shapovalov M (2019) Fish of the Amur River. Apelsin Publishing House, Vladivostok, 317 pp.

[B2] Bănărescu P (1961) Weitere systematische Studien über die Gattung Gobio (Pisces, Cyprinidae), insbesondere im Donaubecken. Věstnik Československé Společnosti Zoologické 25: 318–346.

[B3] Bănărescu P, Nalbant T (1964) Nouveaux goujons des genres Gobio et Squalidus (Pisces, Cyprinidae). Bulletin du Muséum National d’Histoire Naturelle (Série 2): 457–468.

[B4] Bănărescu P, Nalbant T (1966) A new gudgeon of the genus Gobio from Shansi (Pisces, Cyprinidae). American Museum Novitates No. 2275: 1–9.

[B5] Bănărescu P, Nalbant T (1973) Pisces, Teleostei. Cyprinidae (Gobioninae). Walter de Gruyter, Berlin, 304 pp.

[B6] Berg L (1914) Faune de la Russie et des pays limitrophes. Poissons, Marsipobranchii et Pisces. Volume III. Imperatorskii Akademii Nauk, Petrograd, 337–704 pp.

[B7] Bogutskaya N, Naseka A (1997) Cyclostomata and Fishes of Khanka Lake Drainage Area (Amur River basin): An Annotated Check-List with Comments on Taxonomy and Zoogeography of the Region. St. Petersburg, 89 pp.

[B8] Chen JX, Xu QT, Wang XT, Fang SM, Song SL (1987) FIshes in Qin-ling Mountain Area. Science Press, Beijing, 260 pp.

[B9] Cuvier G (1816) Le Règne Animal Distribué D’après son Organisation Pour Servir de Base à L’histoire Naturelle des Animaux et D’introduction à L’anatomie Comparée (Vol. 2). Paris, 532 pp. 10.5962/bhl.title.130093

[B10] Dybowski B (1869) Vorläufige Mittheilungen über die Fischfauna des Ononflusses und des Ingoda in Transbaikalien. Verhandlungen der kaiserlich-königlichen zoologisch-botanischen Gesellschaft in Wien 19: 945–958.

[B11] Fang YT, Sun ZX, Zhao YH (2025) Taxonomic revision on the Gobio group in the Yellow River drainage, with discussion on the validity of the genus Acanthogobio (Cypriniformes, Gobionidae). Zoosystematics and Evolution 101: 661–679. 10.3897/zse.101.144134

[B12] Fricke R, Eschmeyer W, Van der Laan R (2026) Eschmeyer’s Catalog of Fishes: Genera, Species, Reference. http://researcharchive.calacademy.org/research/ichthyology/catalog/fishcatmain.asp [accessed 10 Aug 2026]

[B13] Hammer O, Harper D, Ryan P (2001) PAST: Paleontological Statistics Software Package for Education and Data Analysis. Palaeontologia Electronica 4: 1–9.

[B14] Herzenstein S (1892) Ichthyologische Bemerkungen aus dem Zoologischen Museum der Kaiserlichen Akademie der Wissenschaften. Mélanges Biologiques, tirés du Bulletin physico-mathématique de l’Académie Impériale des Sciences de St Pétersbourg 13: 219–235.

[B15] Hwang D, Lee W, Lee J (2014) Complete mitochondrial genome of the freshwater fish, Acanthorhodeus macropterus (Cypriniformes, Cyprinidae). Mitochondrial DNA 25(1): 11–12. 10.3109/19401736.2013.775265 23527611

[B16] Jelić D, Jelić M, Žutinić P, Šimunović I, Zupančič P, Naseka A (2018) Distribution of endangered Italian gudgeon Romanogobio benacensis (Cypriniformes, Cyprinidae, Gobioninae) with remarks on distinguishing morphological characters. ZooKeys 729: 103–127. 10.3897/zookeys.729.20615 PMC5806454 29430207

[B17] Kalyaanamoorthy S, Minh B, Wong T, Von Haeseler A, Jermiin L (2017) ModelFinder: Fast model selection for accurate phylogenetic estimates. Nature Methods 14: 587–589. 10.1038/nmeth.4285 PMC5453245 28481363

[B18] Kim K, Song H, Kim Y, Park J, Bang I (2021) Complete mitochondrial genome of the Korean endemic species Coreoleuciscus aeruginosus holotype from Korea (Cypriniformes, Cyprinidae). Mitochondrial DNA B Resources 6(3): 1214–1215. 10.1080/23802359.2021.1902415 PMC7995870 33796787

[B19] Kimura M (1980) A simple method for estimating evolutionary rates of base substitutions through comparative studies of nucleotide sequences. Journal of Molecular Evolution 16: 111–120. 10.1007/BF01731581 7463489

[B20] Kottelat M, Persat H (2005) The genus *Gobio* in France, with redescription of *G. gobio* and description of two new species (Teleostei: Cyprinidae). Cybium: International Journal of Ichthyology 29: 211–234. 10.26028/cybium/2005-293-001

[B21] Kumar S, Stecher G, Li M, Knyaz C, Tamura K (2018) MEGA X: Molecular Evolutionary Genetics Analysis across computing platforms. Molecular Biology and Evolution 35: 1547–1549. 10.1093/molbev/msy096 PMC5967553 29722887

[B22] Li SZ (2015) Fishes of the Yellow River. The Sueichan Press, Taiwan, 640 pp.

[B23] Li SZ, Dai DY, Zhang SY, Ma GZ, He ZW, Gao SD (1966) Notes on a collection of fishes from North Sinkiang, China. Acta Zoologica Sinica 18(1): 41–56.

[B24] Li YH, Cao K, Fu CZ (2018) Ten fish mitogenomes of the tribe Gobionini (Cypriniformes: Cyprinidae: Gobioninae). Mitochondrial DNA B Resources 3(2): 803–804. 10.1080/23802359.2018.1467236 PMC7800260 33490537

[B25] Luo YL, Yue PQ, Chen YY (1977) Gobioninae. In: Wu H-W (Ed.) The Cyprinid Fishes of China Vol2. Shanghai Science and Technology Press, Shanghai, 439–549.

[B26] Liu HZ, Yang JQ, Tang QY (2010) Estimated evolutionary tempo of East Asian gobionid fishes (Teleostei: Cyprinidae) from mitochondrial DNA sequence data. Chinese Science Bulletin 55: 1501–1510. 10.1007/s11434-010-3159-7

[B27] Men'shikov MI (1939) On the ichthyofauna of Lake Marka-Kul'. Uchenye zapiski Permskogo universiteta 3(2): 119–142. [In Russian, with English summary]

[B28] Mori T (1930) On the fresh water fishes from the Tumen River, Korea, with description of new species. Journal of the Chosen Natural History Society No. 11: 1–11.

[B29] Mori T (1934) The Fresh Water Fishes of Jehol. Report of the First Scientific Expedition to Manchoukuo. Tokyo, 61 pp.

[B30] Naseka A (1996) Comparative study on the vertebral column in the Gobioninae (Cyprinidae, Pisces) with special reference to its systematics. Publicaciones Especiales Instituto Español de Oceanografía: 149–167.

[B31] Ni XM, Chen Y, Deng GM, Fu CZ (2023) Pleistocene landscape dynamics drives lineage divergence of a temperate freshwater fish *Gobio rivuloides* in coastal drainages of Northern China. Genes 14: 2146. 10.3390/genes14122146 PMC10743038 38136969

[B32] Nichols J (1925) Some Chinese fresh-water fishes. American Museum Novitates No. 181: 1–8.

[B33] Perea S, Böhme M, Zupančič P, Freyhof J, Šanda R, Özuluğ M, Abdoli A, Doadrio I (2010) Phylogenetic relationships and biogeographical patterns in Circum-Mediterranean subfamily Leuciscinae (Teleostei, Cyprinidae) inferred from both mitochondrial and nuclear data. BMC Evolutionary Biology 10: 265. 10.1186/1471-2148-10-265 PMC2940817 20807419

[B34] Qi DL, Yang C (2009) Cloning of mitochondrial cytochrome b gene of Gobio huanghensis and its phylogenetic relationships in genus Gobio. Journal of Qinghai University (Nature Science) 27(3): 38–42.

[B35] Ronquist F, Teslenko M, Van Der Mark P, Ayres D, Darling A, Höhna S, Larget B, Liu L, Suchard M, Huelsenbeck J (2012) MrBayes 3.2: Efficient Bayesian phylogenetic inference and model choice across a large model space. Systematic biology 61: 539–542. 10.1093/sysbio/sys029 PMC3329765 22357727

[B36] Sabaj M (2020) Codes for Natural History Collections in Ichthyology and Herpetology. Copeia 108: 593–669. 10.1643/ASIHCODONS2020

[B37] Sun YT, Wang QL, Ren JF, Yang SB (2020) Identification and phylogenetic analysis of the mitochondrial genome of Hemibarbus labeo BML (Cypriniformes: Cyprinidae). Mitochondrial DNA B Resources 5(3): 2655–2657. 10.1080/23802359.2020.1778570 PMC7782797 33457895

[B38] Swindell S, Plasterer T (1997) SEQMAN. In: Swindell S (Ed.) Sequence Data Analysis Guidebook. Springer New York, Totowa, 75–89. 10.1385/0-89603-358-9:75

[B39] Tan M, Armbruster J (2018) Phylogenetic classification of extant genera of fishes of the order Cypriniformes (Teleostei: Ostariophysi). Zootaxa 4476: 6–39. 10.11646/zootaxa.4476.1.4 30313339

[B40] Tang K, Agnew M, Chen WJ, Raley M, Sado T, Schneider L, Yang L, Bart H, He SP, Liu HZ, Saitoh K, Simons A, Wood R, Mayden R (2011) Phylogeny of the gudgeons (Teleostei: Cyprinidae: Gobioninae). Molecular Phylogenetics and Evolution 61: 103–124. 10.1016/j.ympev.2011.05.022 21672635

[B41] Tchang TL, Shaw TH (1931) Preliminary notes on the cyprinoid fishes of Hopei Province. Bulletin of the Fan Memorial Institute of Biology (Zoological Series) 2: 283–296.

[B42] Vasil’eva E, Mamilov N, Sharakhmetov S (2023) Gudgeon from the Emel River and problems of the gudgeon taxonomy (Genus *Gobio*, Cyprinidae) in Kazakhstan and Siberia. Journal of Icthyology 63: 499–513. 10.31857/S0042875223050156

[B43] Xiao WH, Zhang YP, Liu HZ (2001) Molecular systematics of Xenocyprinae (Teleostei: Cyprinidae): Taxonomy, biogeography, and coevolution of a special group restricted in East Asia. Molecular Phylogenetics and Evolution 18: 163–173. 10.1006/mpev.2000.0879 11161753

[B44] Xie JM, Chen YR, Cai GJ, Cai RL, Hu Z, Wang H (2023) Tree Visualization By One Table (tvBOT): A web application for visualizing, modifying and annotating phylogenetic trees. Nucelic Acids Research 51: W587–W592. 10.1093/nar/gkad359 PMC10320113 37144476

[B45] Xie YH, Li WK, Xie H (2007) Freshwater Fishes in Northeast Region of China. Liaoning Science and Technology Press, Shenyang, 529 pp.

[B46] Yang JQ, He SP, Freyhof J, Witte K, Liu HZ (2006) The phylogenetic relationships of the Gobioninae (Teleostei: Cyprinidae) inferred from mitochondrial cytochrome b gene sequences. Hydrobiologia 553: 255–266. 10.1007/s10750-005-1301-3

[B47] Yue PQ (1998) Gobioninae. In: Chen YY, Chu XL, Luo YL, Chen YR, Liu HZ, He MG (Eds) Fauna Sinica. Osteichthyes. Cypriniformes II. Science Press, Beijing, 232–389. [In Chinese with English summary]

[B48] Zhang CG, Zhao YH, Xing YC, Zhou W, Tang WQ (2016) Species diversity and distribution of inland fishes in China. Science Press, Beijing, 284 pp.

[B49] Zhao YH, Sun ZX (2021) Standard of morphological measurements and description for Cyprinidae, based on an example of Gobionine fishes (Teleostei: Cypriniformes). Bio-101: e1010622. 10.21769/bioprotoc.1010622

[B50] Zheng BS, Huang HM, Zhang YL, Dai DY (1980) Fishes of the Tumen River. Jilin People’s Publishing House, Changchun, 111 pp.

